# The genomic landscape of malignant peripheral nerve sheath tumors: diverse drivers of Ras pathway activation

**DOI:** 10.1038/s41598-017-15183-1

**Published:** 2017-11-08

**Authors:** Andrew S. Brohl, Elliot Kahen, Sean J. Yoder, Jamie K. Teer, Damon R. Reed

**Affiliations:** 1 Sarcoma Department, H. Lee Moffitt Cancer Center and Research Institute, Tampa, FL USA; 2 Chemical Biology & Molecular Medicine Program, H. Lee Moffitt Cancer Center and Research Institute, Tampa, FL USA; 3 Sunshine Project Translational Research Laboratory, H. Lee Moffitt Cancer Center and Research Institute, Tampa, FL USA; 4 Molecular Genomics Core Facility, H. Lee Moffitt Cancer Center and Research Institute, Tampa, FL USA; 5 Department of Biostatistics and Bioinformatics, H. Lee Moffitt Cancer Center and Research Institute, Tampa, FL USA

## Abstract

Malignant peripheral nerve sheath tumor (MPNST) is an aggressive soft tissue sarcoma. To more fully characterize the genomic landscape of this tumor type, we performed next generation sequencing studies for mutational and copy number analysis. We analyzed whole exome sequencing data from 12 MPNST and SNP arrays for a subset of these. We additionally conducted a literature review of prior next generation sequencing studies in this disease and compared to the current study. We report recurrent mutations in *NF1*, *SUZ12*, *EED*, *TP53* and *CDKN2A* in our study cohort. Combined with prior studies, we calculate the disease specific incidence of mutation in these genes to be: *NF1* (56/64 = 87.5%). *SUZ12* (69/123 = 56.1%), *EED* (40/123 = 32.5%), *TP53* (29/72 = 40.3%), and *CDKN2A* (54/72 = 75.0%). Notably, we also identified frequent Ras pathway activating somatic mutations outside of these previously reported recurrently mutated genes. Five of the 12 MPNST in our cohort (42%) contained such a mutation. In conclusion, our study adds to the growing understanding of the genomic complexity of MPNST. We report a previously underappreciated frequency and variety of secondary or tertiary Ras pathway activating mutations, though not highly recurrent in a single gene.

## Introduction

Malignant peripheral nerve sheath tumor (MPNST) is an aggressive subtype of soft tissue sarcoma. Approximately half of MPNSTs are associated with neurofibromatosis type 1 (NF1), and this syndrome is associated with an approximate 10–15% lifetime risk of development of this cancer^1^. Due to this association, classic genetic studies of this malignancy have largely focused on loss of the *NF1* gene as the primary recurrent event. Secondary events in tumor suppressors have also been well described including mutations in *TP53* ^2^ and loss of *CDKN2A*/p16^3^. More recently, next-generation sequencing analysis has revealed recurrent inactivating mutations in *SUZ12* and *EED*, subunits of the Polycomb repressive complex 2 (PRC2), in the majority of cases^4–7^. In MPNST, loss of PRC2 function is believed to amplify Ras pathway activation via direct transcriptional regulatory effects^4,6^. Alternate mechanisms for Ras pathway activation in MPNST have also been described including *BRAF* V600E mutations in a low frequency of patients, perhaps more commonly in sporadic cases^8^.

To more fully characterize the genetic landscape of MPNST, we performed tumor-normal matched whole exome sequencing of six tumors from five patients for both mutational and copy number analysis. We additionally analyzed publically available sequencing data from MPNST from The Cancer Genome Atlas (TCGA) sarcoma project and reviewed and summarized next-generation sequencing data from the literature.

## Results

Whole-exome sequencing was performed on six tumors from five patients, including 5 MPNSTs and 1 atypical neurofibroma and matched germline samples. Four of the five MPNSTs occurred in patients with a clinical diagnosis of NF1 and the remaining case was sporadic. Sequencing was performed to a median depth of 141 million reads [interquartile range (IQR): 54–204 million]. After removal of poor quality and duplicated reads, median sequencing coverage of all samples was 74.2 [IQR: 34.8–101.8] of the target region, with a median of 94.8% [IQR: 90.1–97.3%] of target bases with ≥10x coverage. Seven additional cases of MPNST from TCGA were included in our analysis, including 6 from NF1 patients and 1 sporadic case.

In the twelve MPNST, we observed a median of 63 somatic coding variants per tumor (range 7–472). The somatic coding mutational burden of our in-house samples (range: 68–399, Table S1) was similar to that from the publically available TCGA sample data (range: 7–472). Trinucleotide context of somatic mutations was evaluated to evaluate for similarity to previously reported cancer mutational patterns^9^. In the majority of samples, we observed a type 1 A or 1B mutational signature with prominence of C > T substitutions at NpCpG trinucleotides (Figures S1 and S2). The sole exception was in the highest somatic mutational burden tumor (TCGA-QQ-A8VG), in which we observed a type 7 signature more typical of UV-induced radiation and typified by predominance of C > T substitution in the TpCp(A/C/T) context.

**Figure 1:**
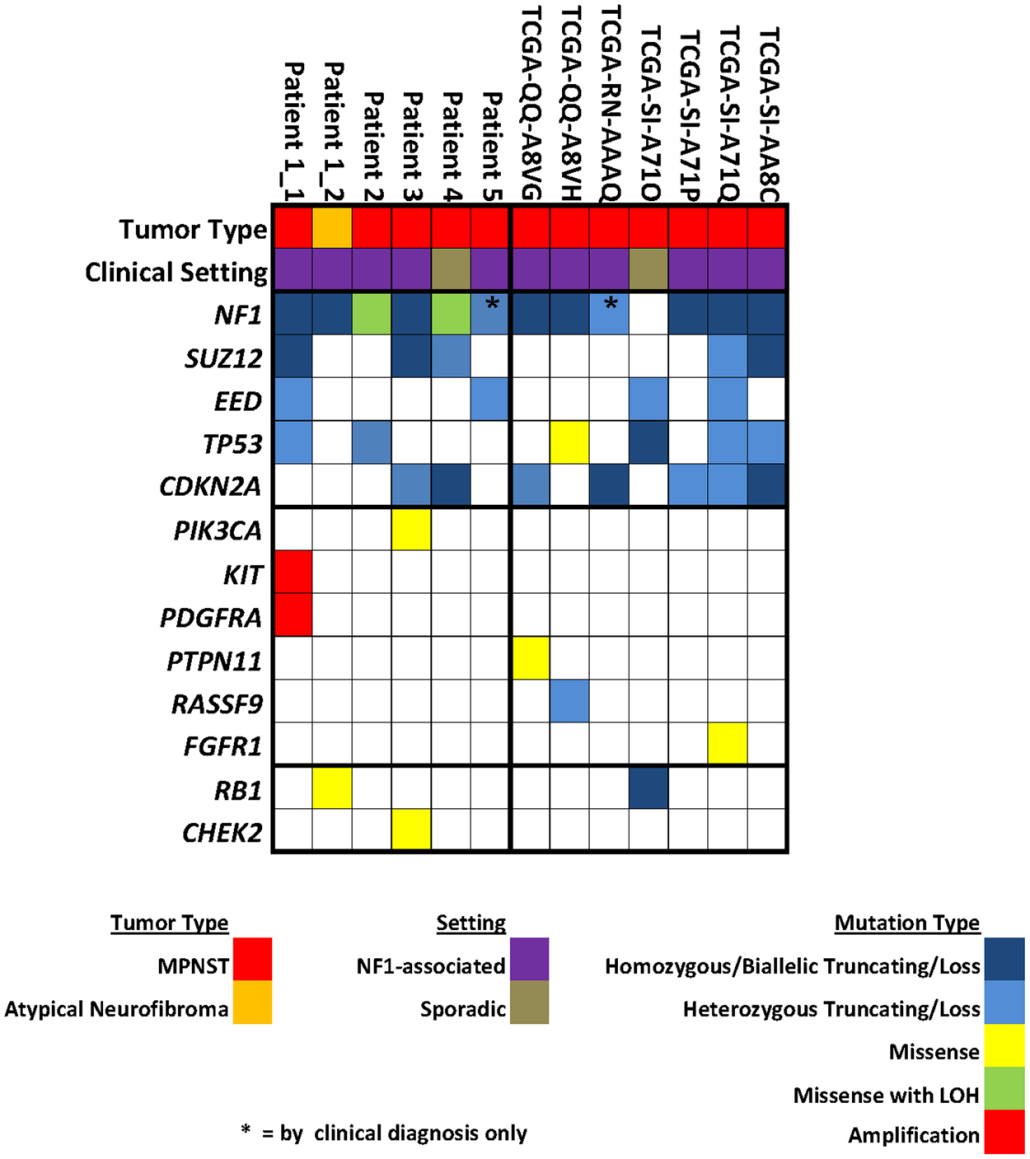
Summary of mutations in study cohort. Mutations listed include only those expected to have pathogenic effect, either due to presence in a mutational hotspot or by an obvious gain of function/loss of function mechanism. Where indicated, patients were considered to have *NF1* mutation based on clinical diagnosis alone (no clinical genetic testing data available). From top to bottom, grouping of findings includes: clinical information, mutation in previously reported recurrently altered genes in MPNST, additional Ras pathway activating mutations, and additional cell-cycle gene mutations.

In MPNST, we observed recurrent mutations in *NF1*, *SUZ12*, *EED*, *TP53* and *CDKN2A* in 92%, 42%, 33%, 50% and 58% respectively of our study population (Fig. 1, Tables S2 and S3). Somatic mutation in *NF1* was observed in 8 of 10 of MPNST from patients with neurofibromatosis and 1 of 2 sporadic cases. Mutation in at least one of the PRC2 components was found in 7 of 12 (58%) of the MPNST. In the one atypical neurofibroma tumor studied, we noted somatic mutation in *NF1* but in none of the other four recurrent genes.

Notably, we observed frequent additional Ras-pathway activating somatic mutations in our cohort, though not significantly recurrent in a single gene (Fig. 1). Affected genes include *PIK3CA*, *KIT*, *PDGFRA*, *PTPN11*, *FGFR1*, and *RASSF9* (Table 1). Five of the 12 MPNST in the study cohort (42%) contained such a mutation. All five cases in which an alternate Ras-pathway mutation was identified were neurofibromatosis-associated. Otherwise, there were no obvious differences between subgroups (PRC2/*TP53*/*CDKN2A* mutated vs. wild type) and frequency of additional Ras-pathway mutations, though numbers were too small for meaningful statistical comparison. Outside of *TP53* and *CDKN2A*, we additionally noted likely pathogenic (inactivating) cell cycle gene mutations in *RB1* in two tumors and *CHEK2* in one (Fig. 1). Sequencing coverage of the recurrently mutated genes, as well as these additional genes of interest, was reasonably high across the majority of the coding regions, limiting the potential for false negative results in these genes (Figure S3).

**Table 1:** Description of Ras activating mutations in non-recurrently mutated genes.

Sample ID	Gene	Alteration
Patient1_1	*KIT/PDGFRA*	focal amplification (CN = 9)
Patient3	*PIK3CA*	E545K
TCGA-QQ-A8VG	*PTPN11*	T52I
TCGA-QQ-A8VH	*RASSF9*	K274X
TCGA-SI-A71Q	*FGFR1*	N546K

In our literature review, we found four prior next-generation sequencing studies of MPNST that met our criteria^4–7^. Frequency of mutation of *NF1, SUZ12, EED, TP53* and *CDKN2A* were similar across studies as well as the current study with several minor exceptions (Table 2). As structural/copy number changes are a frequent source of inactivating mutation in these genes, not unexpectedly the one study that included only limited copy number analysis reported somewhat lower mutational frequencies as these mutation types were not evaluated^5^. Excluding then this one outlier study from a combined analysis, the overall mutational frequency across studies was: *SUZ12* (69/123 = 56.1%), *EED* (40/123 = 32.5%), *TP53* (29/72 = 40.3%), and *CDKN2A* (54/72 = 75.0%). Excluding further the studies that included only NF1 patients, the combined frequency of *NF1* mutation was 56/64 = 87.5%.

**Table 2:** Summary of next generation sequencing studies in MPNST.

Study	year	study size	molecular studies	*NF1*	*SUZ12*	*EED*	*TP53*	*CDKN2A*	Notes
De Raedt, *et al*.^4^	2014	51 MPNST	targeted sequencing, aCGH	NR (clinically 100%)	32/51 (63%)	15/51 (29%)	NR	NR	all NF1 patients
Zhang, *et al*.^5^	2014	50 MPNST	WGS (5), WES (3), targeted sequencing (42)	31/50 (62%)	16/50 (32%)	1/50 (2%)	1/8 (13%)	1/8 (13%)	limited copy number analysis
Lee, *et al*.^6^	2014	52 MPNST	WES, SNP array, and RNAseq (15); targeted sequencing (37)	45/52 (87%)	25/52 (48%)	19/52 (37%)	22/52 (42%)	42/52 (81%)	
Sohier, *et al*.^7^	2017	8 MPNST	WES, aCGH (7)	8/8 (100%)	7/8 (88%)	2/8 (25%)	1/8 (13%)	5/8 (63%)	all NF1 patients
current study	2017	12 MPNST	WES, SNP array (7)	11/12 (92%)	5/12 (42%)	4/12 (33%)	6/12 (50%)	7/12 (58%)	

To confirm the presence of additional alternate Ras-pathway mutations in MPNST, we reviewed somatic mutation calls from prior whole exome or whole genome sequencing (WGS) studies for which this information was provided. This mutational information was available from 23 additional MPNSTs from two studies, in both as part of the supplementary data^5,6^. In our review, we identified 5 mutations in 4 MPNST tumors (17%) that would be expected to be both pathogenic and activating of the Ras pathway, including hotspot mutations in *HRAS*, *PIK3CA*, and *FGFR1* and inactivating mutations in negative Ras regulators *RASA1* and *CNKSR2*.

## Discussion

To our knowledge this is the most comprehensive description of the genomic landscape of malignant peripheral nerve sheath tumor in the literature to date, combining analysis of a whole exome sequencing cohort, publically available data from TCGA, and a review of prior next generation sequencing studies in this disease. First, we recapitulate the previously reported finding of recurrent mutation in *NF1*, *SUZ12*, *EED*, *TP53* and *CDKN2A*. By combining our analysis with the previous literature on this topic, we provide a refined incidence rate of mutation in each of these genes.

Perhaps most notably, our study highlights a diverse range of Ras-activating mutations in MPNST outside of the aforementioned highly recurrently mutated genes, with nearly half to the MPNST in our study cohort having such a mutation. Of note, we purport that our analysis utilized stringent criteria for determination of both pathogenicity of a mutation as well as determination of Ras pathway association suggesting that our estimate of this occurrence is likely to be conservative. In contrast to the current study, prior next-generation sequencing studies in MPNST have not highlighted these alternate Ras-activating mutations^5–7^. We believe that this discrepancy is easily explained by relatively small sample sizes of whole exome/whole genome sequencing cohorts in these prior studies, a desire to focus the discussion on the more highly recurrent mutations, and the fact that these alternate Ras-activating mutations while highly recurrent on the pathway level are not highly recurrent in a single gene. Furthermore, in our review of supplemental findings from these prior studies, we were able to identify 5 instances of alternate Ras-activating mutations from 23 MPNSTs despite the fact that analysis was limited to small variants only.

Previous literature on potential alternate Ras-activating mutations in MPNST includes one prior report of recurrent *BRAF* V600 mutation, particularly in sporadic MPNST^8^. In contrast to this prior study, we detected no *BRAF* V600 mutations in our sequencing cohort or in our review of prior WES/WGS studies, suggesting that this is not a frequent occurrence in MPNST. Furthermore, given the substantial diagnostic overlap between sporadic MPNST and the spindle-cell variant of melanoma, as well as the high frequency of *BRAF* V600 mutation in melanoma, we believe that any “*BRAF* V600 mutant MPNST” should be rigorously evaluated to exclude a misdiagnosis of melanoma. At a minimum, loss of H3K27m3 expression should be evaluated in such patients^10,11^. In a research setting, next-generation sequencing ideally would be performed to evaluate for an ultraviolet (UV) radiation-induced mutational pattern that would be expected in a melanoma.

Our sequencing findings in MPNST raise a larger question in terms of mutual exclusivity of activating mutations within the same pathway. Classically, in many cancer types we tend to think of Ras pathway mutations as being mutually exclusive or at least uncommonly co-occurring. For example, in colorectal carcinomas Ras-MAPK pathway alteration are found in the majority of tumors, with a significant pattern of mutual exclusivity of alteration in *KRAS*, *NRAS* and *BRAF* ^12^. Similarly, receptor tyrosine kinase-Ras-Raf pathway activating mutations are significantly mutually exclusive in lung adenocarcinoma, and recurrent *NF1* losses are enriched in samples lacking an activated oncogene^13^. Cell models suggest that a mechanism for this mutual exclusivity is not merely the lack of a selective advantage of double mutation of a pathway but rather because this leads to senescence in early tumorgenesis^14^. Multiple hits within the same pathway, however, do exist and seem to be context dependent. As an example of this nuance, in melanoma the hot-spot V600 and K601 *BRAF* mutations are significantly exclusive from *NRAS* hot-spot mutations. In contrast, the less common but still recurrent *BRAF* mutations in alternate locations (exon 11, for example) frequently co-occur with a Ras (N/K/H) hot-spot or *NF1* inactivating mutation in this same disease^15^. Taken in whole, we believe that there must be a relative strength to Ras activating mutations that determines whether or not mutations co-occur. Strong drivers like *BRAF* V600 occur in isolation but more intermediate drivers like *BRAF* exon 11 occur typically with other Ras-modifying mutations. Given our findings in MPNST, we hypothesize that *NF1* loss falls into more of the latter category, requiring additional hits to fully activate the Ras pathway to oncogenic levels.

In summary, our study emphasizes that Ras activation in MPNST is complex. In most MPNST, activation of this pathway takes multiple hits, can be activated by a variety of pathways (ex: PRC2/epigenetic) and is activated at multiple positions within the Ras-axis. Future study is warranted to further expand upon potential additional mechanisms of Ras-activation including molecular changes affecting non-coding or regulatory elements of the genome. From a translational standpoint, given the complexity by which Ras is activated in MPNST, we hypothesize that targeting this pathway is unlikely to be successful with inhibition at a single point in the Ras-axis. Importantly, this situation differs on the genomic level from plexiform neurofibroma, in which *NF1* is considered to be a more isolated driver, and where MEK-inhibition has shown promise as a single agent^16,17^.

## Methods

Patients with a diagnosis of MPNST were identified by the treating physician. Samples utilized for sequencing were collected as part of routine clinical care. All subjects gave written institutional review board (IRB)-approved informed consent for their clinical samples to be utilized for research. All methods were carried out in accordance with relevant guidelines and regulations and all experimental protocols were approved by both the local scientific review committee and IRB at the Moffitt Cancer Center.

Whole-exome sequencing (WES) was performed by the Molecular Genomics Core Facility at the Moffitt Cancer Center. The genomic DNA recovered from FFPE blocks was used to generate a sequencing library using the NuGEN Ovation Ultralow Library System (NuGEN, Inc., San Carlos, CA). Whole-exome enrichment utilized the Roche NimbleGen SeqCap EZ Exome Library v2.0 kit, which targets 64 Mb of genomic DNA sequence regions (Roche NimbleGen, Inc., Madison WI). Sequencing was performed on an Illumina HiScan SQ sequencer to generate 75 million 100-base paired-end reads for a target coverage depth of 30–50X. The raw sequence data was demultiplexed using the Illumina CASAVA 1.8.2 software (Illumina, Inc., San Diego, CA). Sequence reads were aligned to the reference human genome (hg19) with the Burrows-Wheeler Alignment Tool^18^, and duplicate identification, insertion/deletion realignment, quality score recalibration, and variant identification were performed with the Picard toolkit and Genome Analysis ToolKit^19^. Somatic mutations were identified by comparing matched tumor and normal samples using Strelka^20^. Sequence variants were annotated to determine genic context using ANNOVAR^21^. Additional contextual information was incorporated, including allele frequency in other studies such as 1000 Genomes, the NHLBI Exome Sequence Project, in silico functional impact predictions, and observed impacts from databases like ClinVar (http://www.ncbi.nlm.nih.gov/clinvar/) and the Collection Of Somatic Mutations In Cancer (COSMIC) (http://cancer.sanger.ac.uk/cosmic).

Processed (Tier 3) data from The Cancer Genome Atlas sarcoma project was obtained from the Genomic Data Commons Data Portal (https://portal.gdc.cancer.gov/) including annotated mutation calls from whole exome sequencing, segmented CNV from SNP arrays, and clinical data. Criteria for inclusion in our analysis included have a listed diagnosis of MPNST with accompanying clinical data to determine syndromic or sporadic and having available processed data from both exome sequencing and SNP array.

Variant analysis from whole exome sequencing from both in-house and TCGA sequencing data was performed by filtering variants to include only those that were rare in population databases (VAF < 0.01) and had compelling evidence for oncogenicity either by presence in a hotspot position in COSMIC, have been previously reported to be oncogenic at the position level, or are a loss of function mutation type (nonsense, frameshift, splice site) in a putative tumor suppressor gene. To screen for mutations that involve the Ras-pathway, we utilized the DAVID tool (https://david.ncifcrf.gov/) for pathway mapping.

Copy number analysis from in-house whole exome sequencing data was performed via paired tumor-normal analysis using EXCAVATOR^22^. Results were further filtered to include only segments with ProbCall >0.9 for novel findings or >0.5 for previously described recurrent CNV in this disease. To further focus on likely pathogenic changes, copy number variation from both WES (in-house) and SNP arrays (TCGA) were further filtered to include for reporting to only segments of predicted high level amplification (CN ≥ 8) or complete deletion (CN = 0) in regions that included an oncogene or tumor suppressor gene, respectively. In addition, we evaluated the genomic regions of the five previously reported recurrently affected tumor suppressors in MPNST (*NF1, SUZ12, EED, TP53*, and *CDKN2A*) for heterozygous loss (CN = 1).

For literature review, a pubmed search was conducted 05/01/2017 using terms “MPNST and sequencing,” “MPNST and genomics”, and “MPNST and PRC2” and the results were manually reviewed. Studies were included for review/analysis if next generation sequencing was performed on at least 5 MPNST.

## Electronic supplementary material

Supplementary Figures and Tables
