# Supplementary material for: The genomic landscape of malignant peripheral nerve sheath tumors: diverse drivers of Ras pathway activation
Source: Sci Rep. 2017 Nov 8;7:14992. doi: 10.1038/s41598-017-15183-1 (PMC5678116; doi:10.1038/s41598-017-15183-1)
Supplement: Supplementary file 1 — Supplementary Figures and Tables [file 41598_2017_15183_MOESM1_ESM.pdf]

The genomic landscape of malignant peripheral nerve sheath tumors: diverse drivers of Ras pathway activation

*Andrew S Brohl[1,2], Elliot Kahen[3], Sean J Yoder[4], Jamie K Teer[5], and Damon R Reed[1,2,3]

[1]Sarcoma Department, [2]Chemical Biology & Molecular Medicine Program, [3]Sunshine Project Translational Research Laboratory, [4]Molecular Genomics Core Facility, and [5]Department of Biostatistics and Bioinformatics, H. Lee Moffitt Cancer Center and Research Institute, Tampa, FL.

*corresponding author:
Andrew S. Brohl, MD
12902 Magnolia Drive
Tampa, FL 33612-9416
813-745-3242 (o)
813-745-8337 (f)
andrew.brohl@moffitt.org

Figure S1: Mutation signatures: in-house samples

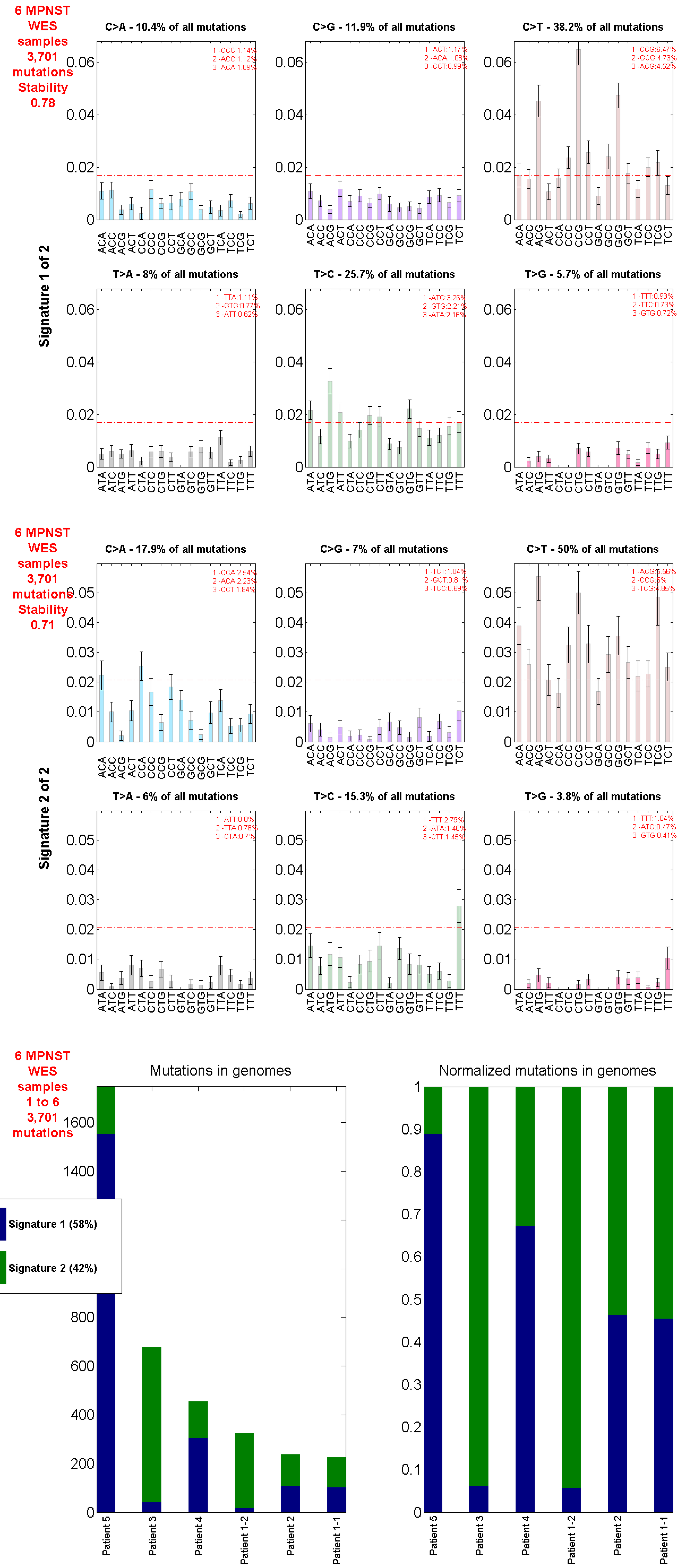

Figure S2: Mutation signatures: TCGA samples

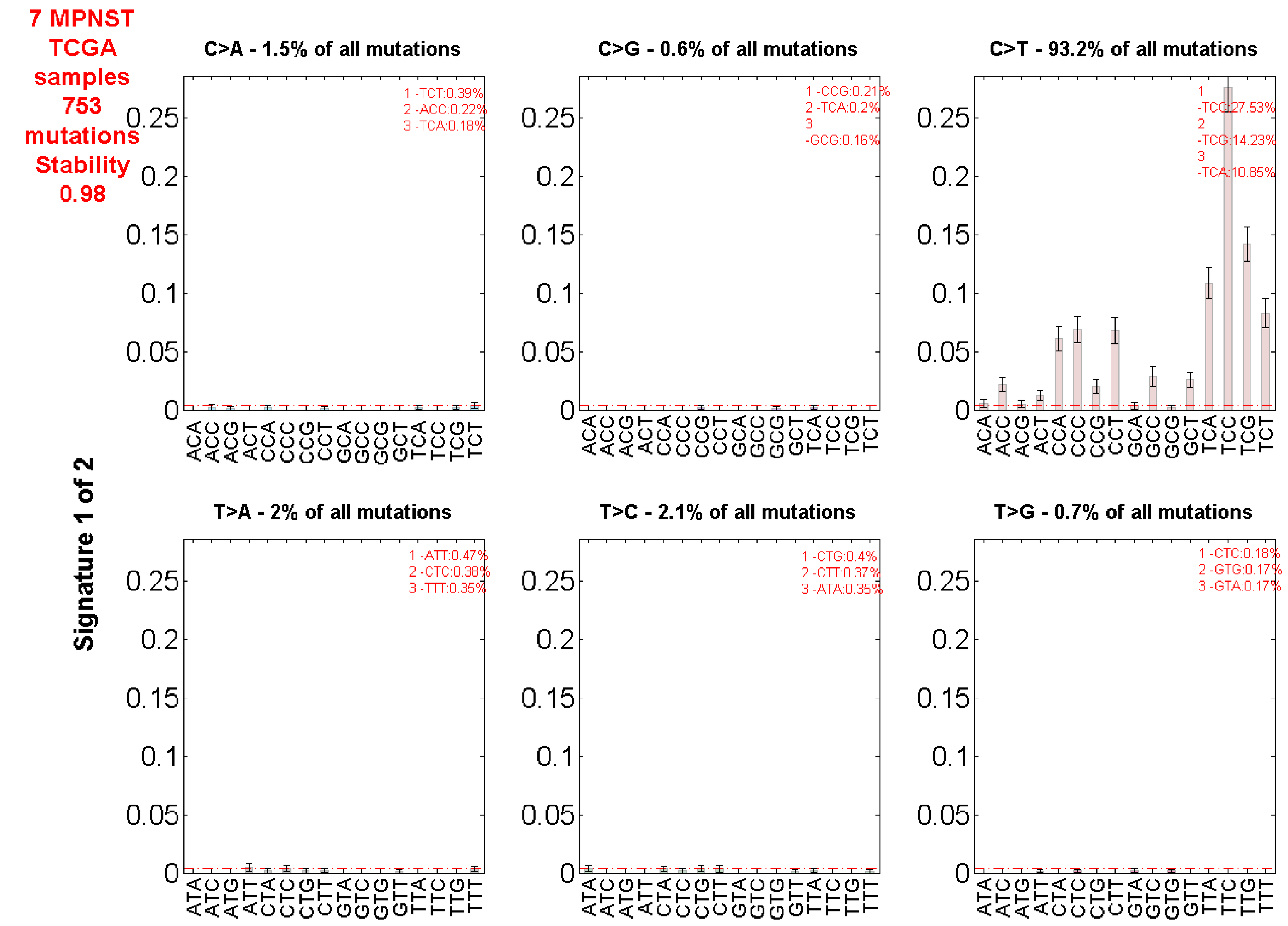

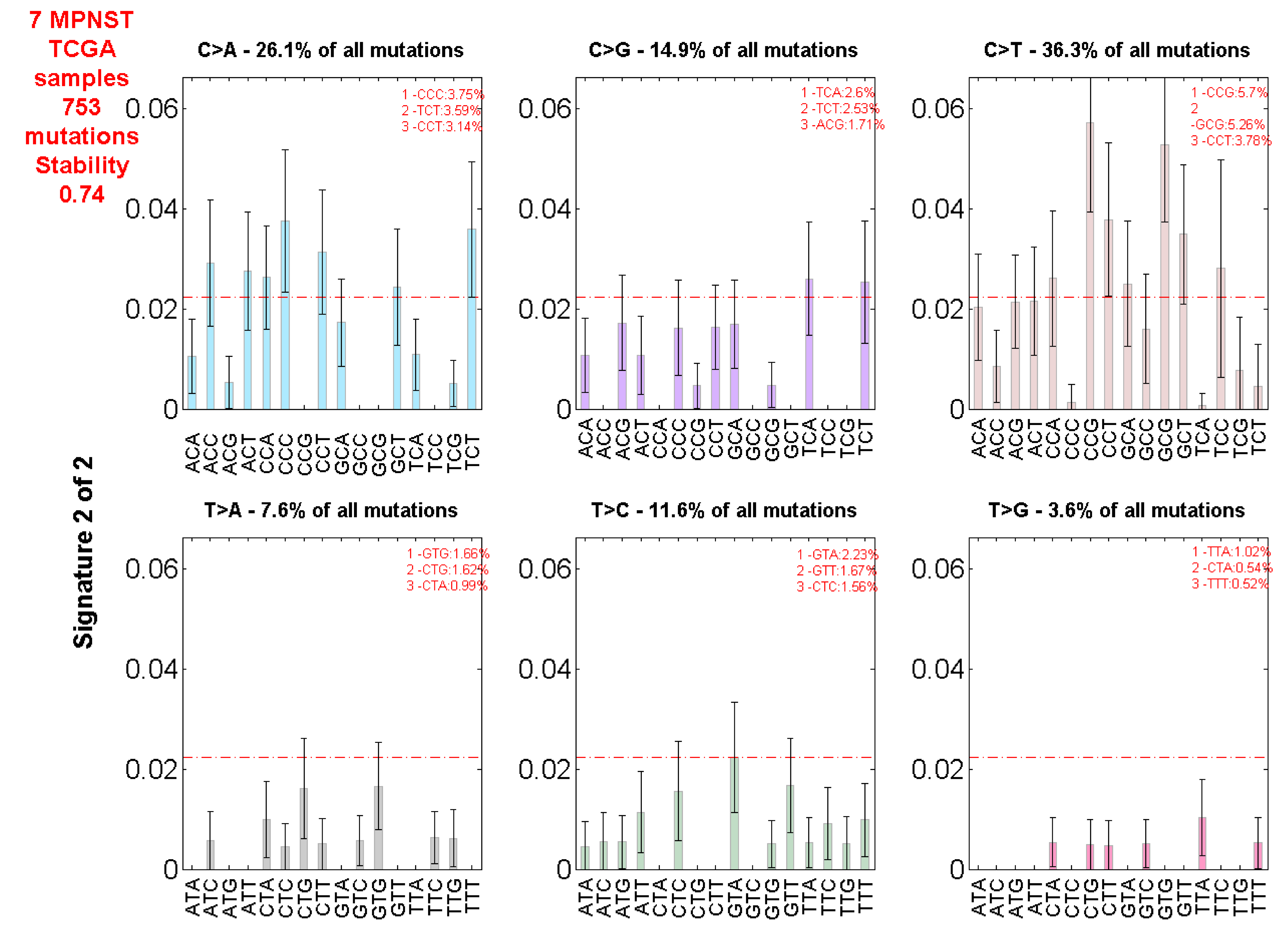

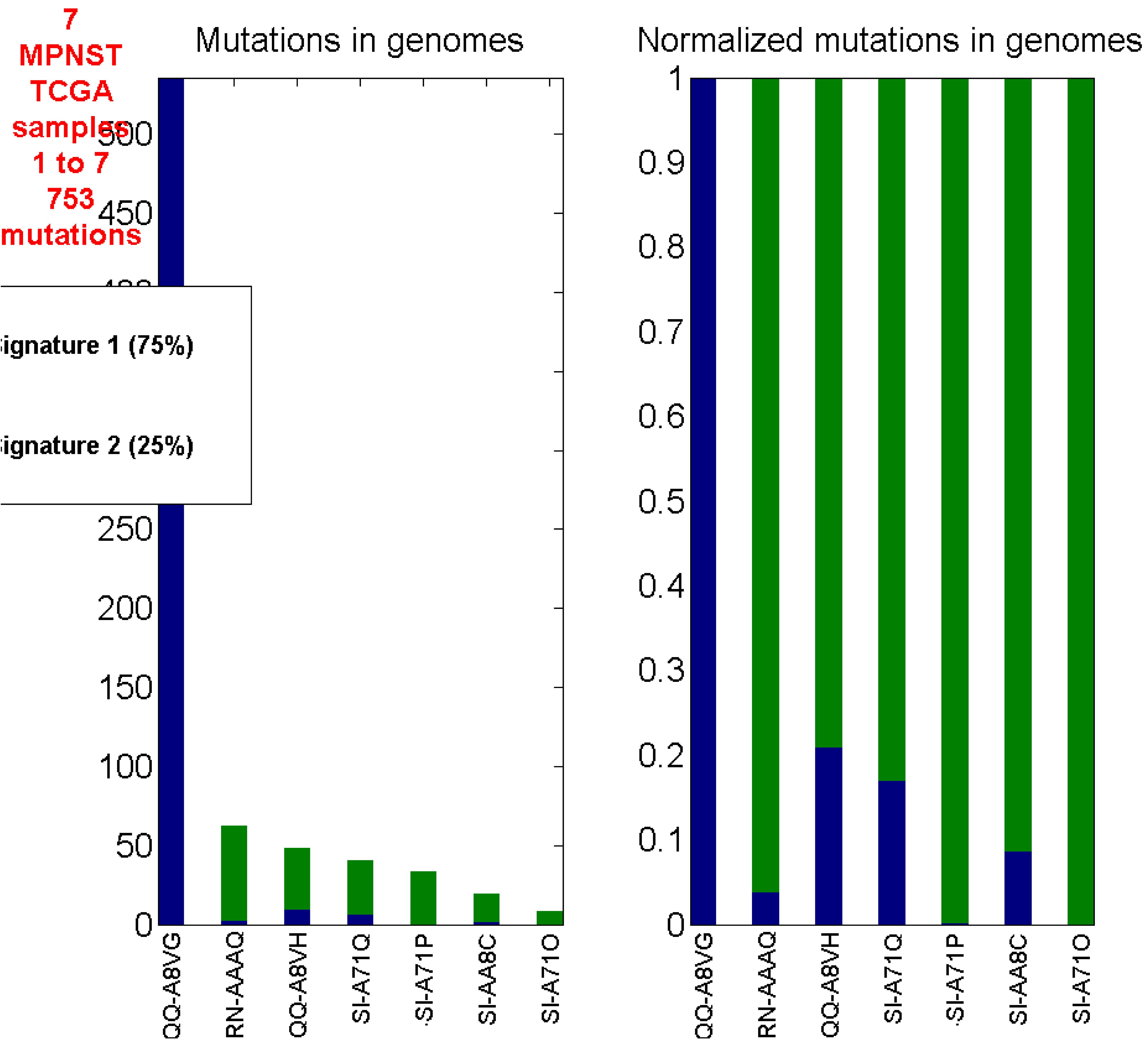

Figure S3: percent of bases with >=10X coverage in key genes

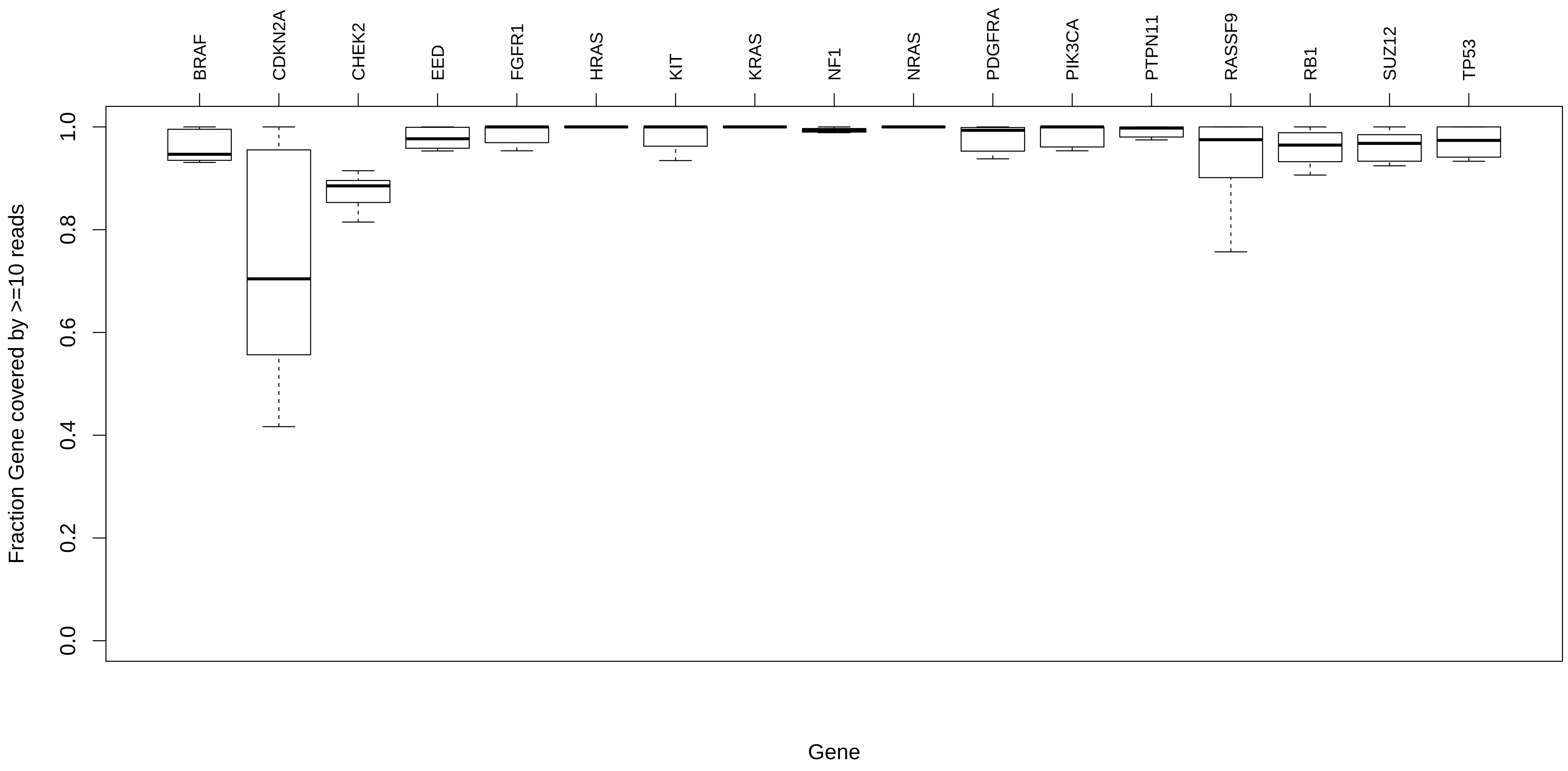

Table S1: Somatic coding variants from in-house samples

Hugo_Symbol	NCBI_Build	Chromosome	Start_Position	End_Position	Variant_Classification	Variant_Type	Reference_Allele	Tumor_Seq_Allele1	Tumor_Seq_Allele2	Tumor_Barcode	Sequence_Source	Sequencer	Annotation_Transcript	Transcript_Exon	cDNA_Change	Protein_Change	Sequencing Depth	Alt_allele_frequency
SPAG17	hs37d5	1	118539001	118539001	nonsynonymous_SNV	SNP	T	T	C	patient 1_1	WXS	Illumina	NM_206996	exon34	c.A5045G	p.E1682G	81	0.4815
FCGR1B	hs37d5	1	120930068	120930068	nonsynonymous_SNV	SNP	G	G	A	patient 1_1	WXS	Illumina	NM_001017986	exon4	c.C533T	p.S178L	183	0.2022
NBPF10	hs37d5	1	145323656	145323656	nonsynonymous_SNV	SNP	A	A	T	patient 1_1	WXS	Illumina	NM_001039703	exon27	c.A3493T	p.I1165F	493	0.0365
NBPF10	hs37d5	1	145323666	145323666	nonsynonymous_SNV	SNP	A	A	G	patient 1_1	WXS	Illumina	NM_001039703	exon27	c.A3503G	p.D1168G	529	0.0737
LMNA	hs37d5	1	156106977	156106977	nonsynonymous_SNV	SNP	G	G	T	patient 1_1	WXS	Illumina	NM_170707	exon9	c.G1562T	p.G521V	103	0.3204
AAK1	hs37d5	2	69734598	69734598	nonsynonymous_SNV	SNP	T	T	C	patient 1_1	WXS	Illumina	NM_014911	exon15	c.A2119G	p.T707A	267	0.206
ZRANB3	hs37d5	2	136107601	136107601	stopgain_SNV	SNP	G	G	A	patient 1_1	WXS	Illumina	NM_032143	exon5	c.C544T	p.R182X	153	0.4575
HSPD1	hs37d5	2	198363406	198363406	nonsynonymous_SNV	SNP	C	C	T	patient 1_1	WXS	Illumina	NM_002156	exon2	c.G167A	p.G56E	197	0.0457
RUFY4	hs37d5	2	218953980	218953980	nonsynonymous_SNV	SNP	A	A	T	patient 1_1	WXS	Illumina	NM_198483	exon12	c.A1508T	p.K503M	72	0.1944
PDIA5	hs37d5	3	122880186	122880186	nonsynonymous_SNV	SNP	G	G	C	patient 1_1	WXS	Illumina	NM_006810	exon16	c.G1363C	p.D455H	106	0.2075
ACPL2	hs37d5	3	141011676	141011676	nonsynonymous_SNV	SNP	G	G	A	patient 1_1	WXS	Illumina	NM_152282	exon8	c.G1072A	p.G358S	126	0.2222
SI	hs37d5	3	164754229	164754229	synonymous_SNV	SNP	A	A	G	patient 1_1	WXS	Illumina	NM_001041	exon22	c.T2463C	p.G821G	252	0.2063
ECE2	hs37d5	3	183975379	183975379	synonymous_SNV	SNP	G	G	A	patient 1_1	WXS	Illumina	NM_014693	exon2	c.G315A	p.P105P	78	0.5385
KCTD8	hs37d5	4	44449779	44449779	synonymous_SNV	SNP	C	C	T	patient 1_1	WXS	Illumina	NM_198353	exon1	c.G762A	p.E254E	204	0.098
LAMTOR3	hs37d5	4	100803220	100803220	synonymous_SNV	SNP	T	T	C	patient 1_1	WXS	Illumina	NM_021970	exon7	c.A306G	p.L102L	95	0.5053
RPS3A	hs37d5	4	152024138	152024138	nonsynonymous_SNV	SNP	A	A	C	patient 1_1	WXS	Illumina	NM_001006	exon4	c.A470C	p.Q157P	70	0.1429
SDHA	hs37d5	5	236696	236696	nonsynonymous_SNV	SNP	G	G	A	patient 1_1	WXS	Illumina	NM_004168	exon10	c.G1414A	p.E472K	211	0.0616
GSTA1	hs37d5	6	52664007	52664007	nonsynonymous_SNV	SNP	C	C	T	patient 1_1	WXS	Illumina	NM_145740	exon2	c.G59A	p.R20Q	169	0.6391
PDE7B	hs37d5	6	136429878	136429878	nonsynonymous_SNV	SNP	G	G	C	patient 1_1	WXS	Illumina	NM_018945	exon3	c.G92C	p.R31P	192	0.2604
MOSPD3	hs37d5	7	100212540	100212540	nonsynonymous_SNV	SNP	G	G	A	patient 1_1	WXS	Illumina	NM_023948	exon4	c.G562A	p.G188R	168	0.4286
MET	hs37d5	7	116415136	116415136	nonsynonymous_SNV	SNP	T	T	C	patient 1_1	WXS	Illumina	NM_001127500	exon15	c.T3284C	p.I1095T	267	0.4607
SHH	hs37d5	7	155604640	155604640	nonsynonymous_SNV	SNP	G	G	C	patient 1_1	WXS	Illumina	NM_000193	exon1	c.C177G	p.S59R	210	0.2143
ATP6V1B2	hs37d5	8	20055020	20055020	nonsynonymous_SNV	SNP	G	G	C	patient 1_1	WXS	Illumina	NM_001693	exon1	c.G103C	p.V35L	105	0.4
TEX15	hs37d5	8	30694400	30694400	nonsynonymous_SNV	SNP	C	C	G	patient 1_1	WXS	Illumina	NM_031271	exon3	c.G8251C	p.A2751P	88	0.3636
PDE7A	hs37d5	8	66647090	66647090	nonsynonymous_SNV	SNP	T	T	C	patient 1_1	WXS	Illumina	NM_001242318	exon7	c.A635G	p.H212R	113	0.292
RIMS2	hs37d5	8	105026745	105026745	nonsynonymous_SNV	SNP	G	G	A	patient 1_1	WXS	Illumina	NM_001100117	exon19	c.G3236A	p.R1079H	339	0.2124
PKHD1L1	hs37d5	8	110451270	110451270	nonsynonymous_SNV	SNP	G	G	A	patient 1_1	WXS	Illumina	NM_177531	exon32	c.G3905A	p.G1302E	291	0.488
ADARB2	hs37d5	10	1262895	1262895	nonsynonymous_SNV	SNP	C	C	T	patient 1_1	WXS	Illumina	NM_018702	exon7	c.G1678A	p.A560T	139	0.0504
MUC5B	hs37d5	11	1262615	1262615	nonsynonymous_SNV	SNP	C	C	A	patient 1_1	WXS	Illumina	NM_002458	exon31	c.C4505A	p.T1502N	84	0.1786
OSBPL5	hs37d5	11	3114887	3114887	nonsynonymous_SNV	SNP	C	C	G	patient 1_1	WXS	Illumina	NM_020896	exon17	c.G1816C	p.D606H	93	0.1828
CNGA4	hs37d5	11	6265203	6265203	nonsynonymous_SNV	SNP	C	C	T	patient 1_1	WXS	Illumina	NM_001037329	exon6	c.C1292T	p.T431I	110	0.1818
OR5T1	hs37d5	11	56043638	56043638	nonsynonymous_SNV	SNP	C	C	T	patient 1_1	WXS	Illumina	NM_001004745	exon1	c.C524T	p.T175I	240	0.2292
RAB3IL1	hs37d5	11	61672115	61672115	nonsynonymous_SNV	SNP	G	G	A	patient 1_1	WXS	Illumina	NM_013401	exon7	c.C802T	p.R268W	69	0.0725
SLC25A45	hs37d5	11	65144314	65144314	synonymous_SNV	SNP	C	C	T	patient 1_1	WXS	Illumina	NM_182556	exon6	c.G573A	p.Q191Q	61	0.082
RACGAP1	hs37d5	12	50385793	50385793	nonsynonymous_SNV	SNP	G	G	A	patient 1_1	WXS	Illumina	NM_001126104	exon15	c.C1702T	p.P568S	87	0.069
RACGAP1	hs37d5	12	50385814	50385814	nonsynonymous_SNV	SNP	C	C	T	patient 1_1	WXS	Illumina	NM_001126104	exon15	c.G1681A	p.A561T	89	0.0674
RACGAP1	hs37d5	12	50385815	50385815	synonymous_SNV	SNP	A	A	G	patient 1_1	WXS	Illumina	NM_001126104	exon15	c.T1680C	p.N560N	89	0.0674
RACGAP1	hs37d5	12	50385837	50385837	nonsynonymous_SNV	SNP	A	A	T	patient 1_1	WXS	Illumina	NM_001126104	exon15	c.T1658A	p.L553Q	92	0.0652
RACGAP1	hs37d5	12	50385838	50385838	nonsynonymous_SNV	SNP	G	G	T	patient 1_1	WXS	Illumina	NM_001126104	exon15	c.C1657A	p.L553I	93	0.0645
RACGAP1	hs37d5	12	50385851	50385851	synonymous_SNV	SNP	C	C	T	patient 1_1	WXS	Illumina	NM_001126104	exon15	c.G1644A	p.E548E	88	0.0682
SCYL2	hs37d5	12	100732902	100732902	nonsynonymous_SNV	SNP	G	G	A	patient 1_1	WXS	Illumina	NM_017988	exon18	c.G2742A	p.M914I	195	0.4513
CLIP1	hs37d5	12	122825575	122825575	nonsynonymous_SNV	SNP	C	C	G	patient 1_1	WXS	Illumina	NM_001247997	exon11	c.G2176C	p.V726L	156	0.2564
ACIN1	hs37d5	14	23532672	23532672	nonsynonymous_SNV	SNP	G	G	T	patient 1_1	WXS	Illumina	NM_014977	exon13	c.C2884A	p.P962T	103	0.0583
SCFD1	hs37d5	14	31103210	31103210	nonsynonymous_SNV	SNP	G	G	C	patient 1_1	WXS	Illumina	NM_016106	exon4	c.G279C	p.M93I	131	0.2901
GPR176	hs37d5	15	40099328	40099328	nonsynonymous_SNV	SNP	T	T	C	patient 1_1	WXS	Illumina	NM_007223	exon2	c.A304G	p.S102G	162	0.1852
EHD4	hs37d5	15	42193240	42193240	nonsynonymous_SNV	SNP	T	T	C	patient 1_1	WXS	Illumina	NM_139265	exon6	c.A1229G	p.Q410R	51	0.1569
RFX7	hs37d5	15	56387061	56387067	nonframeshift_deletion	DEL	AGTGGGT	AGTGGGT	A	patient 1_1	WXS	Illumina	NM_022841	exon9	c.2859_2864del	p.953_955del	288	0.0174
CORO2B	hs37d5	15	69007559	69007559	synonymous_SNV	SNP	T	T	C	patient 1_1	WXS	Illumina	NM_006091	exon8	c.T876C	p.D292D	100	0.18
TPSAB1	hs37d5	16	1291621	1291621	synonymous_SNV	SNP	C	C	T	patient 1_1	WXS	Illumina	NM_003294	exon4	c.C420T	p.V140V	74	0.2162
PMFBP1	hs37d5	16	72163056	72163056	nonsynonymous_SNV	SNP	C	C	T	patient 1_1	WXS	Illumina	NM_031293	exon13	c.G1859A	p.R620Q	139	0.0504
SUZ12	hs37d5	17	30323850	30323850	stopgain_SNV	SNP	G	G	T	patient 1_1	WXS	Illumina	NM_015355	exon15	c.G1828T	p.E610X	196	0.9388
FAM171A2	hs37d5	17	42437404	42437404	nonsynonymous_SNV	SNP	G	G	A	patient 1_1	WXS	Illumina	NM_198475	exon2	c.C215T	p.A72V	82	0.3049
GAA	hs37d5	17	78087081	78087081	nonsynonymous_SNV	SNP	G	G	C	patient 1_1	WXS	Illumina	NM_000152	exon15	c.G2105C	p.R702P	232	0.1078
EPB41L3	hs37d5	18	5489039	5489039	synonymous_SNV	SNP	G	G	A	patient 1_1	WXS	Illumina	NM_012307	exon2	c.C144T	p.F48F	59	0.2542
C18orf1	hs37d5	18	13621231	13621231	synonymous_SNV	SNP	C	C	T	patient 1_1	WXS	Illumina	NM_181481	exon5	c.C297T	p.R99R	58	0.2586
ZFP36	hs37d5	19	39898474	39898474	nonsynonymous_SNV	SNP	G	G	T	patient 1_1	WXS	Illumina	NM_003407	exon2	c.G116T	p.S39I	178	0.1236
PLA2G4C	hs37d5	19	48578097	48578097	synonymous_SNV	SNP	A	A	T	patient 1_1	WXS	Illumina	NM_001159322	exon12	c.T963A	p.T321T	11	0.6364
PLA2G4C	hs37d5	19	48578098	48578098	nonsynonymous_SNV	SNP	G	G	C	patient 1_1	WXS	Illumina	NM_001159322	exon12	c.C962G	p.T321S	8	0.5
GNRH2	hs37d5	20	3026345	3026350	frameshift_deletion	DEL	AGCCCC	AGCCCC	A	patient 1_1	WXS	Illumina	NM_001501	exon4	c.327_331del	p.109_111del	233	0.0215
NAA20	hs37d5	20	20006379	20006379	nonsynonymous_SNV	SNP	C	C	T	patient 1_1	WXS	Illumina	NM_016100	exon3	c.C137T	p.A46V	169	0.4379
NAA20	hs37d5	20	20007547	20007547	nonsynonymous_SNV	SNP	G	G	C	patient 1_1	WXS	Illumina	NM_016100	exon4	c.G289C	p.E97Q	330	0.697
MICALL1	hs37d5	22	38327834	38327834	nonsynonymous_SNV	SNP	G	G	A	patient 1_1	WXS	Illumina	NM_033386	exon10	c.G1910A	p.R637Q	93	0.0538
ARHGAP6	hs37d5	X	11204551	11204551	splicing	SNP	C	C	A	patient 1_1	WXS	Illumina	0	0	0	0	51	0.2549
PRPS1	hs37d5	X	106890893	106890893	synonymous_SNV	SNP	C	C	T	patient 1_1	WXS	Illumina	NM_002764	exon6	c.C762T	p.S254S	219	0.1689
ABCD1	hs37d5	X	152991103	152991103	nonsynonymous_SNV	SNP	C	C	T	patient 1_1	WXS	Illumina	NM_000033	exon1	c.C382T	p.R128W	295	0.1051
WDTC1	hs37d5	1	27622852	27622852	synonymous_SNV	SNP	G	G	A	patient 1_2	WXS	Illumina	NM_015023	exon10	c.G909A	p.P303P	98	0.0612
AK2	hs37d5	1	33502343	33502343	synonymous_SNV	SNP	C	C	G	patient 1_2	WXS	Illumina	NM_001625	exon1	c.G87C	p.G29G	68	0.0882
RHBDL2	hs37d5	1	39384769	39384769	nonsynonymous_SNV	SNP	C	C	T	patient 1_2	WXS	Illumina	NM_017821	exon2	c.G116A	p.R39Q	46	0.1087
C1orf50	hs37d5	1	43239306	43239306	nonsynonymous_SNV	SNP	G	G	A	patient 1_2	WXS	Illumina	NM_024097	exon3	c.G268A	p.E90K	96	0.0625
TSHB	hs37d5	1	115576805	115576805	nonsynonymous_SNV	SNP	G	G	T	patient 1_2	WXS	Illumina	NM_000549	exon3	c.G374T	p.C125F	84	0.0714
NBPF10	hs37d5	1	145323656	145323656	nonsynonymous_SNV	SNP	A	A	T	patient 1_2	WXS	Illumina	NM_001039703	exon27	c.A3493T	p.I1165F	153	0.0915
NBPF10	hs37d5	1	145323666	145323666	nonsynonymous_SNV	SNP	A	A	G	patient 1_2	WXS	Illumina	NM_001039703	exon27	c.A3503G	p.D1168G	166	0.0663
NUF2	hs37d5	1	163310178	163310178	nonsynonymous_SNV	SNP	C	C	A	patient 1_2	WXS	Illumina	NM_031423	exon9	c.C631A	p.Q211K	113	0.0531
POLR1A	hs37d5	2	86258692	86258692	nonsynonymous_SNV	SNP	C	C	T	patient 1_2	WXS	Illumina	NM_015425	exon30	c.G4339A	p.E1447K	116	0.0517
RGPD3	hs37d5	2	107049414	107049414	nonsynonymous_SNV	SNP	G	G	A	patient 1_2	WXS	Illumina	NM_001144013	exon17	c.C2446T	p.R816C	74	0.1081
RGPD3	hs37d5	2	107049425	107049425	nonsynonymous_SNV	SNP	A	A	C	patient 1_2	WXS	Illumina	NM_001144013	exon17	c.T2435G	p.L812R	87	0.1264
TUBA3E	hs37d5	2	130951857	130951857	synonymous_SNV	SNP	G	G	A	patient 1_2	WXS	Illumina	NM_207312	exon4	c.C558T	p.N186N	59	0.0847
TANC1	hs37d5	2	160005664	160005664	nonsynonymous_SNV	SNP	G	G	A	patient 1_2	WXS	Illumina	NM_033394	exon6	c.G376A	p.D126N	62	0.0806
DYNC1I2	hs37d5	2	172585298	172585298	synonymous_SNV	SNP	T	T	C	patient 1_2	WXS	Illumina	NM_001378	exon14	c.T1329C	p.D443D	113	0.0708
HSPD1	hs37d5	2	198363406	198363406	nonsynonymous_SNV	SNP	C	C	T	patient 1_2	WXS	Illumina	NM_002156	exon2	c.G167A	p.G56E	92	0.0978
ICA1L	hs37d5	2	203679493	203679493	synonymous_SNV	SNP	C	C	T	patient 1_2	WXS	Illumina	NM_138468	exon9	c.G798A	p.T266T	169	0.0414
CXXC11	hs37d5	2	242812041	242812041	stopgain_SNV	SNP	C	C	T	patient 1_2	WXS	Illumina	NM_173821	exon1	c.C133T	p.Q45X	67	0.0896
LRRN1	hs37d5	3	3886416	3886416	stopgain_SNV	SNP	G	G	T	patient 1_2	WXS	Illumina	NM_020873	exon2	c.G91T	p.E31X	44	0.1136
NEK10	hs37d5	3	27346306	27346306	synonymous_SNV	SNP	C	C	T	patient 1_2	WXS	Illumina	NM_199347	exon13	c.G960A	p.E320E	26	0.1538
KBTBD5	hs37d5	3	42730422	42730422	nonsynonymous_SNV	SNP	C	C	T	patient 1_2	WXS	Illumina	NM_152393	exon4	c.C1483T	p.P495S	17	0.2353
PLXND1	hs37d5	3	129291727	129291727	synonymous_SNV	SNP	G	G	A	patient 1_2	WXS	Illumina	NM_015103	exon14	c.C2895T	p.N965N	15	0.2667
MAP3K13	hs37d5	3	185167722	185167722	nonsynonymous_SNV	SNP	G	G	A	patient 1_2	WXS	Illumina	NM_004721	exon6	c.G1045A	p.G349R	124	0.0484
TMEM44	hs37d5	3	194344368	194344368	synonymous_SNV	SNP	G	G	A	patient 1_2	WXS	Illumina	NM_001166305	exon4	c.C415T	p.L139L	23	0.1739
KLHL2	hs37d5	4	166231773	166231773	nonsynonymous_SNV	SNP	G	G	A	patient 1_2	WXS	Illumina	NM_001161521	exon10	c.G1120A	p.V374I	106	0.0566
CCT5	hs37d5	5	10250521	10250521	synonymous_SNV	SNP	C	C	T	patient 1_2	WXS	Illumina	NM_012073	exon1	c.C69T	p.D23D	45	0.1111
FAM169A	hs37d5	5	74077349	74077349	synonymous_SNV	SNP	C	C	T	patient 1_2	WXS	Illumina	NM_015566	exon13	c.A1949A	p.K650K	87	0.069
OPN5	hs37d5	6	47775895	47775895	synonymous_SNV	SNP	G	G	A	patient 1_2	WXS	Illumina	NM_181744	exon5	c.G762A	p.A254A	92	0.0652
PKHD1	hs37d5	6	51875132	51875132	nonsynonymous_SNV	SNP	C	C	T	patient 1_2	WXS	Illumina	NM_138694	exon35	c.G5726A	p.R1909Q	115	0.0522
BCLAF1	hs37d5	6	136600951	136600978	nonframeshift_deletion	DEL	CTGTGATCTAGACTTTGACCTTGAAGAA	CTGTGATCTAGACTTTGACCTTGAAGAA	C	patient 1_2	WXS	Illumina	NM_014739	exon3	c.27_53del	p.9_18del	131	0.0305
PNLDC1	hs37d5	6	160237603	160237603	synonymous_SNV	SNP	G	G	A	patient 1_2	WXS	Illumina	NM_173516	exon14	c.G1056A	p.A352A	92	0.0652
DACT2	hs37d5	6	168711854	168711854	synonymous_SNV	SNP	G	G	A	patient 1_2	WXS	Illumina	NM_214462	exon2	c.C357T	p.D119D	18	0.2222
CNTNAP2	hs37d5	7	148080871	148080871	synonymous_SNV	SNP	C	C	T	patient 1_2	WXS	Illumina	NM_014141	exon22	c.C3606T	p.H1202H	20	0.2
ASAH1	hs37d5	8	17928835	17928835	nonsynonymous_SNV	SNP	C	C	T	patient 1_2	WXS	Illumina	NM_004315	exon3	c.G238A	p.E80K	107	0.0561
RNF19A	hs37d5	8	101273882	101273882	stopgain_SNV	SNP	G	G	A	patient 1_2	WXS	Illumina	NM_015435	exon9	c.C1570T	p.R524X	97	0.0619
CRB2	hs37d5	9	126132557	126132557	nonsynonymous_SNV	SNP	G	G	A	patient 1_2	WXS	Illumina	NM_173689	exon7	c.G1225A	p.A409T	36	0.1389
TUBB4B	hs37d5	9	140136190	140136190	synonymous_SNV	SNP	C	C	T	patient 1_2	WXS	Illumina	NM_006088	exon2	c.C75T	p.S25S	45	0.1111
PDSS1	hs37d5	10	27009231	27009231	nonsynonymous_SNV	SNP	C	C	A	patient 1_2	WXS	Illumina	NM_014317	exon6	c.C552A	p.D184E	75	0.08
RBP3	hs37d5	10	48389275	48389275	nonsynonymous_SNV	SNP	G	G	A	patient 1_2	WXS	Illumina	NM_002900	exon1	c.C1603T	p.R535C	16	0.25
MAT1A	hs37d5	10	82034850	82034850	nonsynonymous_SNV	SNP	G	G	A	patient 1_2	WXS	Illumina	NM_000429	exon7	c.C874T	p.R292C	28	0.1429
PLCE1	hs37d5	10	96018679	96018679	splicing	SNP	G	G	A	patient 1_2	WXS	Illumina	0	0	0	0	97	0.1031
NSMCE4A	hs37d5	10	123719016	123719016	splicing	SNP	C	C	T	patient 1_2	WXS	Illumina	0	0	0	0	84	0.0714
STX3	hs37d5	11	59560589	59560589	nonsynonymous_SNV	SNP	G	G	A	patient 1_2	WXS	Illumina	NM_004177	exon7	c.G484A	p.D162N	71	0.0845
PLCB3	hs37d5	11	64031040	64031040	nonsynonymous_SNV	SNP	G	G	A	patient 1_2	WXS	Illumina	NM_000932	exon20	c.G2426A	p.R809Q	57	0.0877
MAML2	hs37d5	11	95825374	95825374	synonymous_SNV	SNP	T	T	C	patient 1_2	WXS	Illumina	NM_032427	exon2	c.A1821G	p.Q607Q	133	0.0451
DSCAML1	hs37d5	11	117299107	117299107	synonymous_SNV	SNP	C	C	T	patient 1_2	WXS	Illumina	NM_020693	exon33	c.G6279A	p.S2093S	70	0.0714
ERC1	hs37d5	12	1292505	1292505	nonsynonymous_SNV	SNP	A	A	G	patient 1_2	WXS	Illumina	NM_178040	exon11	c.A2075G	p.K692R	114	0.0439
PFKM	hs37d5	12	48535545	48535545	stopgain_SNV	SNP	G	G	T	patient 1_2	WXS	Illumina	NM_001166686	exon18	c.G1648T	p.E550X	59	0.1017
DGKA	hs37d5	12	56345866	56345866	synonymous_SNV	SNP	G	G	A	patient 1_2	WXS	Illumina	NM_201444	exon19	c.G1635A	p.P545P	16	0.25
RB1	hs37d5	13	48954342	48954342	nonsynonymous_SNV	SNP	C	C	T	patient 1_2	WXS	Illumina	NM_000321	exon16	c.C1463T	p.A488V	179	0.0615
TXNDC16	hs37d5	14	52937265	52937265	nonsynonymous_SNV	SNP	C	C	A	patient 1_2	WXS	Illumina	NM_020784	exon15	c.G1446T	p.M482I	74	0.0811
AP5M1	hs37d5	14	57746961	57746961	nonsynonymous_SNV	SNP	C	C	A	patient 1_2	WXS	Illumina	NM_018229	exon3	c.C769A	p.P257T	62	0.0968
UBR7	hs37d5	14	93685641	93685641	nonsynonymous_SNV	SNP	G	G	T	patient 1_2	WXS	Illumina	NM_175748	exon8	c.G894T	p.K298N	62	0.0968
DYNC1H1	hs37d5	14	102483207	102483207	synonymous_SNV	SNP	C	C	T	patient 1_2	WXS	Illumina	NM_001376	exon38	c.C7719T	p.D2573D	12	0.3333
FAM189A1	hs37d5	15	29488674	29488674	nonsynonymous_SNV	SNP	G	G	A	patient 1_2	WXS	Illumina	NM_015307	exon4	c.C482T	p.P161L	10	0.4
GJD2	hs37d5	15	35046684	35046684	synonymous_SNV	SNP	C	C	T	patient 1_2	WXS	Illumina	NM_020660	exon1	c.G6A	p.G2G	27	0.1481
LCTL	hs37d5	15	66855942	66855942	nonsynonymous_SNV	SNP	C	C	T	patient 1_2	WXS	Illumina	NM_207338	exon4	c.G392A	p.G131E	54	0.1111
CALML4	hs37d5	15	68497711	68497711	nonsynonymous_SNV	SNP	C	C	T	patient 1_2	WXS	Illumina	NM_033429	exon1	c.G4A	p.A2T	36	0.1389
KRT15	hs37d5	17	39671797	39671797	synonymous_SNV	SNP	G	G	A	patient 1_2	WXS	Illumina	NM_002275	exon6	c.C1174T	p.L392L	20	0.2
TMEM106A	hs37d5	17	41368528	41368528	nonsynonymous_SNV	SNP	G	G	A	patient 1_2	WXS	Illumina	NM_145041	exon6	c.G490A	p.E164K	71	0.0704
TTLL6	hs37d5	17	46874447	46874447	synonymous_SNV	SNP	G	G	A	patient 1_2	WXS	Illumina	NM_001130918	exon7	c.C888T	p.Y296Y	95	0.0632
QRICH2	hs37d5	17	74288292	74288292	nonsynonymous_SNV	SNP	C	C	T	patient 1_2	WXS	Illumina	NM_032134	exon4	c.G2018A	p.R673H	47	0.1277
RGL3	hs37d5	19	11516069	11516069	nonsynonymous_SNV	SNP	G	G	A	patient 1_2	WXS	Illumina	NM_001161616	exon8	c.C1030T	p.R344C	53	0.0943
ZNF563	hs37d5	19	12429792	12429792	synonymous_SNV	SNP	G	G	T	patient 1_2	WXS	Illumina	NM_145276	exon4	c.C1047A	p.P349P	91	0.0659
GLT25D1	hs37d5	19	17692154	17692154	synonymous_SNV	SNP	G	G	A	patient 1_2	WXS	Illumina	NM_024656	exon12	c.G1770A	p.K590K	17	0.2353
ZNF493	hs37d5	19	21607133	21607133	nonsynonymous_SNV	SNP	T	T	C	patient 1_2	WXS	Illumina	NM_001076678	exon4	c.T1672C	p.S558P	89	0.0674
ZNF229	hs37d5	19	44933863	44933863	nonsynonymous_SNV	SNP	G	G	C	patient 1_2	WXS	Illumina	NM_014518	exon6	c.C1093G	p.L365V	21	0.1905
CKM	hs37d5	19	45818776	45818776	nonsynonymous_SNV	SNP	G	G	A	patient 1_2	WXS	Illumina	NM_001824	exon4	c.C428T	p.P143L	23	0.1739
ZNF114	hs37d5	19	48789694	48789694	synonymous_SNV	SNP	C	C	T	patient 1_2	WXS	Illumina	NM_153608	exon5	c.C813T	p.T271T	14	0.2857
RNF24	hs37d5	20	3944656	3944656	synonymous_SNV	SNP	C	C	T	patient 1_2	WXS	Illumina	NM_001134338	exon3	c.G72A	p.S24S	59	0.0847
NINL	hs37d5	20	25478895	25478895	nonsynonymous_SNV	SNP	C	C	T	patient 1_2	WXS	Illumina	NM_025176	exon9	c.G1120A	p.V374I	28	0.1429
RIMS4	hs37d5	20	43385568	43385568	nonsynonymous_SNV	SNP	G	G	A	patient 1_2	WXS	Illumina	NM_001205317	exon5	c.C565T	p.P189S	53	0.1132
NFATC2	hs37d5	20	50049027	50049027	stopgain_SNV	SNP	G	G	A	patient 1_2	WXS	Illumina	NM_173091	exon9	c.C2299T	p.Q767X	64	0.0938
SLC17A9	hs37d5	20	61597000	61597000	synonymous_SNV	SNP	C	C	T	patient 1_2	WXS	Illumina	NM_022082	exon10	c.C984T	p.C328C	19	0.2105
COL20A1	hs37d5	20	61961003	61961003	nonsynonymous_SNV	SNP	G	G	T	patient 1_2	WXS	Illumina	NM_020882	exon35	c.G3848T	p.W1283L	16	0.25
CBS	hs37d5	21	44478946	44478946	synonymous_SNV	SNP	C	C	T	patient 1_2	WXS	Illumina	NM_000071	exon14	c.G1356A	p.A452A	15	0.2667
CLTCL1	hs37d5	22	19263320	19263320	stopgain_SNV	SNP	C	C	A	patient 1_2	WXS	Illumina	NM_007098	exon2	c.G76T	p.G26X	58	0.0862
IL3RA	hs37d5	X	1464262	1464262	nonsynonymous_SNV	SNP	A	A	G	patient 1_2	WXS	Illumina	NM_002183	exon3	c.A118G	p.T40A	152	0.0592
IL3RA	hs37d5	X	1464276	1464276	nonsynonymous_SNV	SNP	C	C	A	patient 1_2	WXS	Illumina	NM_002183	exon3	c.C132A	p.N44K	166	0.0542
NLGN4X	hs37d5	X	5821136	5821136	nonsynonymous_SNV	SNP	G	G	A	patient 1_2	WXS	Illumina	NM_181332	exon5	c.C1583T	p.T528M	94	0.0745
PORCN	hs37d5	X	48370301	48370301	synonymous_SNV	SNP	C	C	T	patient 1_2	WXS	Illumina	NM_203475	exon3	c.C351T	p.T117T	24	0.1667
TARDBP	hs37d5	1	11078880	11078880	stopgain_SNV	SNP	C	C	T	patient 2	WXS	Illumina	NM_007375	exon4	c.C493T	p.R165X	17	0.2353
PRAMEF1	hs37d5	1	12854474	12854474	nonsynonymous_SNV	SNP	A	A	C	patient 2	WXS	Illumina	NM_023013	exon3	c.A698C	p.N233T	69	0.2464
PRAMEF1	hs37d5	1	12854479	12854479	nonsynonymous_SNV	SNP	C	C	G	patient 2	WXS	Illumina	NM_023013	exon3	c.C703G	p.R235G	68	0.2353
SSBP3	hs37d5	1	54870262	54870262	nonsynonymous_SNV	SNP	G	G	A	patient 2	WXS	Illumina	NM_145716	exon3	c.C178T	p.H60Y	99	0.0606
LOR	hs37d5	1	153233503	153233503	synonymous_SNV	SNP	C	C	T	patient 2	WXS	Illumina	NM_000427	exon2	c.C78T	p.G26G	22	0.1818
RBBP5	hs37d5	1	205065819	205065819	nonsynonymous_SNV	SNP	G	G	T	patient 2	WXS	Illumina	NM_005057	exon12	c.C1387A	p.P463T	119	0.0504
OR2M5	hs37d5	1	248309020	248309020	nonsynonymous_SNV	SNP	G	G	A	patient 2	WXS	Illumina	NM_001004690	exon1	c.G571A	p.D191N	130	0.0615
PXDN	hs37d5	2	1687445	1687445	nonsynonymous_SNV	SNP	C	C	A	patient 2	WXS	Illumina	NM_012293	exon6	c.G543T	p.L181F	141	0.0426
THADA	hs37d5	2	43459878	43459878	nonsynonymous_SNV	SNP	C	C	T	patient 2	WXS	Illumina	NM_022065	exon37	c.G5440A	p.V1814M	58	0.431
SNRNP200	hs37d5	2	96944703	96944703	nonsynonymous_SNV	SNP	G	G	A	patient 2	WXS	Illumina	NM_014014	exon37	c.C5167T	p.P1723S	106	0.0566
FOXD4L1	hs37d5	2	114257590	114257590	nonsynonymous_SNV	SNP	G	G	C	patient 2	WXS	Illumina	NM_012184	exon1	c.G757C	p.A253P	105	0.1048
FOXD4L1	hs37d5	2	114257595	114257595	synonymous_SNV	SNP	C	C	G	patient 2	WXS	Illumina	NM_012184	exon1	c.C762G	p.P254P	104	0.0962
LY75-CD302	hs37d5	2	160698768	160698768	stopgain_SNV	SNP	G	G	A	patient 2	WXS	Illumina	NM_001198759	exon24	c.C3268T	p.Q1090X	164	0.0793
HSPD1	hs37d5	2	198363501	198363501	synonymous_SNV	SNP	C	C	T	patient 2	WXS	Illumina	NM_002156	exon2	c.G72A	p.R24R	190	0.0789
STK36	hs37d5	2	219543926	219543926	synonymous_SNV	SNP	G	G	A	patient 2	WXS	Illumina	NM_015690	exon7	c.G720A	p.R240R	140	0.2
CLSTN2	hs37d5	3	140140115	140140115	splicing	SNP	A	A	G	patient 2	WXS	Illumina	0	0	0	0	59	0.0678
MUC20	hs37d5	3	195453249	195453249	nonsynonymous_SNV	SNP	T	T	C	patient 2	WXS	Illumina	NM_152673	exon3	c.T1262C	p.M421T	57	0.1754
SKIV2L2	hs37d5	5	54654496	54654496	nonsynonymous_SNV	SNP	G	G	A	patient 2	WXS	Illumina	NM_015360	exon15	c.G1629A	p.M543I	64	0.0781
DMXL1	hs37d5	5	118469289	118469289	stopgain_SNV	SNP	T	T	A	patient 2	WXS	Illumina	NM_005509	exon12	c.T1670A	p.L557X	83	0.3253
MUC21	hs37d5	6	30954395	30954395	nonsynonymous_SNV	SNP	G	G	A	patient 2	WXS	Illumina	NM_001010909	exon2	c.G443A	p.S148N	139	0.1151
HLA-DQA2	hs37d5	6	32713771	32713771	nonsynonymous_SNV	SNP	T	T	C	patient 2	WXS	Illumina	NM_020056	exon3	c.T535C	p.F179L	186	0.1882
HLA-DQA2	hs37d5	6	32713788	32713788	nonsynonymous_SNV	SNP	T	T	G	patient 2	WXS	Illumina	NM_020056	exon3	c.T552G	p.D184E	195	0.1385
HLA-DQA2	hs37d5	6	32713793	32713793	nonsynonymous_SNV	SNP	T	T	G	patient 2	WXS	Illumina	NM_020056	exon3	c.T557G	p.I186S	201	0.1443
HLA-DQA2	hs37d5	6	32713821	32713821	synonymous_SNV	SNP	C	C	A	patient 2	WXS	Illumina	NM_020056	exon3	c.C585A	p.G195G	210	0.1333
HLA-DQA2	hs37d5	6	32714091	32714091	nonsynonymous_SNV	SNP	A	A	G	patient 2	WXS	Illumina	NM_020056	exon4	c.A688G	p.M230V	168	0.0774
TBP	hs37d5	6	170871073	170871073	synonymous_SNV	SNP	G	G	A	patient 2	WXS	Illumina	NM_003194	exon3	c.G249A	p.Q83Q	81	0.0741
MUC17	hs37d5	7	100677338	100677338	nonsynonymous_SNV	SNP	G	G	C	patient 2	WXS	Illumina	NM_001040105	exon3	c.G2641C	p.A881P	193	0.3316
ALKBH4	hs37d5	7	102100207	102100207	synonymous_SNV	SNP	G	G	A	patient 2	WXS	Illumina	NM_017621	exon2	c.C165T	p.A55A	78	0.0641
CFTR	hs37d5	7	117188763	117188763	synonymous_SNV	SNP	C	C	T	patient 2	WXS	Illumina	NM_000492	exon10	c.C1278T	p.D426D	206	0.0437
PABPC1	hs37d5	8	101727750	101727750	nonsynonymous_SNV	SNP	T	T	C	patient 2	WXS	Illumina	NM_002568	exon4	c.A583G	p.I195V	193	0.0725
PABPC1	hs37d5	8	101727766	101727766	synonymous_SNV	SNP	T	T	C	patient 2	WXS	Illumina	NM_002568	exon4	c.A567G	p.E189E	209	0.0622
PRRX2	hs37d5	9	132481604	132481604	synonymous_SNV	SNP	G	G	A	patient 2	WXS	Illumina	NM_016307	exon2	c.G354A	p.A118A	98	0.0612
ANO3	hs37d5	11	26529783	26529783	nonsynonymous_SNV	SNP	G	G	C	patient 2	WXS	Illumina	NM_031418	exon5	c.G565C	p.A189P	46	0.7826
LOC283116	hs37d5	11	49053424	49053424	synonymous_SNV	SNP	A	A	G	patient 2	WXS	Illumina	NM_001206626	exon1	c.A273G	p.Q91Q	190	0.1368
LOC283116	hs37d5	11	49053488	49053488	synonymous_SNV	SNP	T	T	C	patient 2	WXS	Illumina	NM_001206626	exon1	c.T337C	p.L113L	172	0.1279
TRIM64C	hs37d5	11	49075409	49075409	synonymous_SNV	SNP	G	G	A	patient 2	WXS	Illumina	NM_001206631	exon6	c.C1210T	p.L404L	166	0.8072
OR4A5	hs37d5	11	51411879	51411879	nonsynonymous_SNV	SNP	G	G	T	patient 2	WXS	Illumina	NM_001005272	exon1	c.C517A	p.H173N	105	0.6571
ARHGEF17	hs37d5	11	73071488	73071488	nonsynonymous_SNV	SNP	G	G	A	patient 2	WXS	Illumina	NM_014786	exon11	c.G4330A	p.E1444K	30	0.1333
WNK1	hs37d5	12	936244	936244	synonymous_SNV	SNP	T	T	A	patient 2	WXS	Illumina	NM_001184985	exon3	c.T969A	p.V323V	67	0.0746
WNK1	hs37d5	12	936245	936245	synonymous_SNV	SNP	C	C	T	patient 2	WXS	Illumina	NM_001184985	exon3	c.C970T	p.L324L	71	0.0704
TNFRSF1A	hs37d5	12	6443003	6443003	synonymous_SNV	SNP	G	G	A	patient 2	WXS	Illumina	NM_001065	exon3	c.C222T	p.G74G	27	0.1481
FAM186A	hs37d5	12	50745677	50745677	synonymous_SNV	SNP	T	T	A	patient 2	WXS	Illumina	NM_001145475	exon4	c.A4938T	p.A1646A	95	0.0737
ESYT1	hs37d5	12	56526281	56526281	nonsynonymous_SNV	SNP	G	G	A	patient 2	WXS	Illumina	NM_001184796	exon9	c.G1061A	p.G354D	26	0.1538
LRP1	hs37d5	12	57593003	57593003	stopgain_SNV	SNP	C	C	T	patient 2	WXS	Illumina	NM_002332	exon61	c.C9685T	p.Q3229X	19	0.5263
TPTE2	hs37d5	13	19999116	19999116	nonsynonymous_SNV	SNP	C	C	A	patient 2	WXS	Illumina	NM_199254	exon20	c.G1437T	p.W479C	152	0.4474
SERPINA9	hs37d5	14	94929473	94929473	nonsynonymous_SNV	SNP	C	C	T	patient 2	WXS	Illumina	NM_175739	exon5	c.G1265A	p.G422D	117	0.1197
GLCE	hs37d5	15	69548187	69548187	synonymous_SNV	SNP	T	T	C	patient 2	WXS	Illumina	NM_015554	exon3	c.T42C	p.I14I	88	0.2045
BAIAP3	hs37d5	16	1392727	1392727	nonsynonymous_SNV	SNP	C	C	T	patient 2	WXS	Illumina	NM_003933	exon13	c.C1178T	p.T393M	38	0.1053
C16orf52	hs37d5	16	22019669	22019669	nonsynonymous_SNV	SNP	A	A	G	patient 2	WXS	Illumina	NM_001164579	exon1	c.A47G	p.D16G	178	0.3876
PDPR	hs37d5	16	70164399	70164399	synonymous_SNV	SNP	C	C	T	patient 2	WXS	Illumina	NM_017990	exon7	c.C681T	p.T227T	46	0.1087
ZNF469	hs37d5	16	88505334	88505334	nonsynonymous_SNV	SNP	A	A	G	patient 2	WXS	Illumina	NM_001127464	exon2	c.A11372G	p.K3791R	41	0.0976
EFCAB5	hs37d5	17	28320330	28320330	synonymous_SNV	SNP	T	T	A	patient 2	WXS	Illumina	NM_198529	exon5	c.T915A	p.P305P	93	0.0753
ARHGAP23	hs37d5	17	36636008	36636008	nonsynonymous_SNV	SNP	C	C	T	patient 2	WXS	Illumina	NM_001199417	exon14	c.C2513T	p.P838L	33	0.1818
KRTAP1-3	hs37d5	17	39190819	39190819	stopgain_SNV	SNP	A	A	T	patient 2	WXS	Illumina	NM_030966	exon1	c.T255A	p.C85X	143	0.3986
KRTAP4-7	hs37d5	17	39240627	39240627	nonsynonymous_SNV	SNP	T	T	C	patient 2	WXS	Illumina	NM_033061	exon1	c.T169C	p.S57P	221	0.0814
REEP6	hs37d5	19	1491356	1491356	nonsynonymous_SNV	SNP	G	G	A	patient 2	WXS	Illumina	NM_138393	exon1	c.G88A	p.G30R	21	0.1905

TCF3	hs37d5	19	1611721	1611721	synonymous_SNV	SNP	G	G	A	patient 2	WXS	Illumina	NM_003200	exon19	c.C1950T	p.P650P	76	0.0789
KDM4B	hs37d5	19	5077409	5077409	synonymous_SNV	SNP	C	C	T	patient 2	WXS	Illumina	NM_015015	exon8	c.C708T	p.D236D	52	0.4231
KEAP1	hs37d5	19	10602588	10602588	synonymous_SNV	SNP	G	G	A	patient 2	WXS	Illumina	NM_012289	exon3	c.C990T	p.T330T	55	0.4545
FAM129C	hs37d5	19	17648324	17648324	synonymous_SNV	SNP	G	G	T	patient 2	WXS	Illumina	NM_173544	exon6	c.G660T	p.L220L	19	0.7368
CCDC124	hs37d5	19	18054434	18054434	synonymous_SNV	SNP	G	G	A	patient 2	WXS	Illumina	NM_138442	exon5	c.G582A	p.S194S	57	0.1228
RHPN2	hs37d5	19	33490566	33490566	nonsynonymous_SNV	SNP	T	T	C	patient 2	WXS	Illumina	NM_033103	exon10	c.A1151G	p.Q384R	113	0.2301
UBA2	hs37d5	19	34949785	34949785	nonsynonymous_SNV	SNP	C	C	T	patient 2	WXS	Illumina	NM_005499	exon13	c.C1357T	p.R453W	84	0.0595
ZNF28	hs37d5	19	53304627	53304627	synonymous_SNV	SNP	T	T	C	patient 2	WXS	Illumina	NM_006969	exon4	c.A471G	p.E157E	177	0.0508
ZNF28	hs37d5	19	53304632	53304632	nonsynonymous_SNV	SNP	G	G	A	patient 2	WXS	Illumina	NM_006969	exon4	c.C466T	p.P156S	177	0.0565
ZNF28	hs37d5	19	53304673	53304673	nonsynonymous_SNV	SNP	A	A	G	patient 2	WXS	Illumina	NM_006969	exon4	c.T425C	p.L142S	179	0.0335
SALL4	hs37d5	20	50407254	50407254	nonsynonymous_SNV	SNP	C	C	T	patient 2	WXS	Illumina	NM_020436	exon2	c.G1768A	p.G590R	41	0.8537
ZDHHC8	hs37d5	22	20131071	20131071	nonsynonymous_SNV	SNP	C	C	T	patient 2	WXS	Illumina	NM_001185024	exon10	c.C1918T	p.R640W	31	0.129
SLC9A7	hs37d5	X	46502776	46502776	frameshift_insertion	INS	G	G	GATGCCGT	patient 2	WXS	Illumina	NM_032591	exon12	.1508_1509insACGGC	p.S503fs	30	0.7333
ITIH6	hs37d5	X	54777561	54777561	nonsynonymous_SNV	SNP	C	C	T	patient 2	WXS	Illumina	NM_198510	exon12	c.G3605A	p.G1202E	59	0.4746
AGRN	hs37d5	1	981213	981213	synonymous_SNV	SNP	G	G	A	patient 3	WXS	Illumina	NM_198576	exon15	c.G2637A	p.Q879Q	87	0.2069
PPIAL4G	hs37d5	1	143767495	143767495	synonymous_SNV	SNP	C	C	T	patient 3	WXS	Illumina	NM_001123068	exon1	c.G354A	p.K118K	597	0.0737
CHIT1	hs37d5	1	203192647	203192647	nonsynonymous_SNV	SNP	C	C	A	patient 3	WXS	Illumina	NM_003465	exon5	c.G456T	p.K152N	117	0.1197
RPS6KC1	hs37d5	1	213303056	213303056	nonsynonymous_SNV	SNP	G	G	A	patient 3	WXS	Illumina	NM_012424	exon6	c.G659A	p.R220Q	135	0.1778
PCNXL2	hs37d5	1	233152844	233152844	synonymous_SNV	SNP	T	T	C	patient 3	WXS	Illumina	NM_014801	exon27	c.A4662G	p.E1554E	300	0.2767
SNTG2	hs37d5	2	1133331	1133331	nonsynonymous_SNV	SNP	G	G	A	patient 3	WXS	Illumina	NM_018968	exon5	c.G349A	p.V117I	118	0.0508
KLHL29	hs37d5	2	23916221	23916221	synonymous_SNV	SNP	C	C	T	patient 3	WXS	Illumina	NM_052920	exon8	c.C1365T	p.A455A	31	0.1613
VIT	hs37d5	2	36986167	36986167	synonymous_SNV	SNP	G	G	A	patient 3	WXS	Illumina	NM_053276	exon6	c.G465A	p.S155S	72	0.0694
PCBP1	hs37d5	2	70315397	70315397	synonymous_SNV	SNP	G	G	A	patient 3	WXS	Illumina	NM_006196	exon1	c.G522A	p.P174P	98	0.0612
GPAT2	hs37d5	2	96690344	96690344	synonymous_SNV	SNP	C	C	T	patient 3	WXS	Illumina	NM_207328	exon15	c.G1500A	p.Q500Q	44	0.1136
FER1L5	hs37d5	2	97335887	97335887	nonsynonymous_SNV	SNP	C	C	T	patient 3	WXS	Illumina	NM_001113382	exon17	c.C1379T	p.A460V	139	0.0504
ARHGAP15	hs37d5	2	144194568	144194568	synonymous_SNV	SNP	C	C	T	patient 3	WXS	Illumina	NM_018460	exon8	c.C660T	p.D220D	101	0.0594
NEB	hs37d5	2	152496901	152496901	nonsynonymous_SNV	SNP	G	G	T	patient 3	WXS	Illumina	NM_001164508	exon61	c.C8653A	p.H2885N	87	0.069
IFIH1	hs37d5	2	163163282	163163282	nonsynonymous_SNV	SNP	C	C	T	patient 3	WXS	Illumina	NM_022168	exon3	c.G706A	p.E236K	165	0.0545
DAZL	hs37d5	3	16639980	16639980	nonsynonymous_SNV	SNP	A	A	T	patient 3	WXS	Illumina	NM_001190811	exon2	c.T189A	p.F63L	217	0.0369
C3orf38	hs37d5	3	88202388	88202388	nonsynonymous_SNV	SNP	C	C	T	patient 3	WXS	Illumina	NM_173824	exon2	c.C142T	p.H48Y	45	0.2444
ILDR1	hs37d5	3	121712483	121712483	synonymous_SNV	SNP	G	G	A	patient 3	WXS	Illumina	NM_001199799	exon7	c.C1113T	p.H371H	62	0.0968
KALRN	hs37d5	3	123953765	123953765	nonsynonymous_SNV	SNP	G	G	A	patient 3	WXS	Illumina	NM_001024660	exon3	c.G232A	p.V78M	89	0.0674
PIK3CA	hs37d5	3	178936091	178936091	nonsynonymous_SNV	SNP	G	G	A	patient 3	WXS	Illumina	NM_006218	exon10	c.G1633A	p.E545K	136	0.2647
CHRD	hs37d5	3	184100892	184100892	nonsynonymous_SNV	SNP	G	G	T	patient 3	WXS	Illumina	NM_003741	exon10	c.G1154T	p.W385L	54	0.1111
TEC	hs37d5	4	48178149	48178149	nonsynonymous_SNV	SNP	C	C	A	patient 3	WXS	Illumina	NM_003215	exon3	c.G193T	p.V65L	155	0.0645
ERVMER34-1	hs37d5	4	53610357	53610357	nonsynonymous_SNV	SNP	C	C	T	patient 3	WXS	Illumina	NM_001242690	exon3	c.G1331A	p.C444Y	96	0.0833
UGT2A1	hs37d5	4	70455322	70455322	nonsynonymous_SNV	SNP	G	G	A	patient 3	WXS	Illumina	NM_001252274	exon7	c.C1850T	p.P617L	115	0.3739
DCK	hs37d5	4	71892384	71892384	nonsynonymous_SNV	SNP	C	C	A	patient 3	WXS	Illumina	NM_000788	exon6	c.C668A	p.T223N	95	0.0632
CNOT6L	hs37d5	4	78663449	78663449	splicing	SNP	C	C	T	patient 3	WXS	Illumina	0	0	0	0	90	0.5222
CLGN	hs37d5	4	141327237	141327237	splicing	SNP	C	C	T	patient 3	WXS	Illumina	0	0	0	0	51	0.3922
BRD9	hs37d5	5	876251	876251	nonsynonymous_SNV	SNP	C	C	T	patient 3	WXS	Illumina	NM_023924	exon12	c.G1348A	p.G450R	74	0.0811
RXFP3	hs37d5	5	33938135	33938135	synonymous_SNV	SNP	G	G	A	patient 3	WXS	Illumina	NM_016568	exon1	c.G1290A	p.G430G	36	0.5278
PDLIM4	hs37d5	5	131607136	131607136	nonsynonymous_SNV	SNP	G	G	A	patient 3	WXS	Illumina	NM_003687	exon5	c.G647A	p.G216D	100	0.06
HSPA4	hs37d5	5	132424760	132424760	nonsynonymous_SNV	SNP	C	C	T	patient 3	WXS	Illumina	NM_002154	exon10	c.C1151T	p.S384L	144	0.1597
SEC24A	hs37d5	5	134010371	134010395	nonframeshift_deletion	DEL	TACCACCTGGTTATCAGAACACAAC	TACCACCTGGTTATCAGAACACAAC	T	patient 3	WXS	Illumina	NM_021982	exon5	c.885_908del	p.295_303del	148	0.027
PCDHB15	hs37d5	5	140626800	140626800	nonsynonymous_SNV	SNP	C	C	A	patient 3	WXS	Illumina	NM_018935	exon1	c.C1654A	p.L552M	128	0.1406
RELL2	hs37d5	5	141017926	141017926	nonsynonymous_SNV	SNP	G	G	A	patient 3	WXS	Illumina	NM_173828	exon1	c.G134A	p.R45H	84	0.0714
FBXO38	hs37d5	5	147785874	147785874	nonsynonymous_SNV	SNP	G	G	A	patient 3	WXS	Illumina	NM_205836	exon7	c.G785A	p.R262Q	213	0.0376
C5orf54	hs37d5	5	159821792	159821792	nonsynonymous_SNV	SNP	C	C	T	patient 3	WXS	Illumina	NM_022090	exon2	c.G706A	p.V236I	46	0.5217
SCGN	hs37d5	6	25661781	25661781	splicing	SNP	A	A	G	patient 3	WXS	Illumina	0	0	0	0	147	0.0544
PRRC2A	hs37d5	6	31601297	31601297	nonsynonymous_SNV	SNP	A	A	T	patient 3	WXS	Illumina	NM_080686	exon17	c.A4461T	p.Q1487H	114	0.0614
RPF2	hs37d5	6	111329246	111329246	nonsynonymous_SNV	SNP	T	T	A	patient 3	WXS	Illumina	NM_032194	exon7	c.T399A	p.S133R	198	0.1566
ZC3H12D	hs37d5	6	149771839	149771839	nonsynonymous_SNV	SNP	C	C	T	patient 3	WXS	Illumina	NM_207360	exon6	c.G1564A	p.A522T	60	0.2833
TMEM181	hs37d5	6	159001992	159001992	nonsynonymous_SNV	SNP	T	T	G	patient 3	WXS	Illumina	NM_020823	exon3	c.T544G	p.S182A	94	0.0638
IGF2R	hs37d5	6	160461668	160461668	synonymous_SNV	SNP	C	C	T	patient 3	WXS	Illumina	NM_000876	exon11	c.C1392T	p.Y464Y	72	0.0833
MACC1	hs37d5	7	20199734	20199734	nonsynonymous_SNV	SNP	G	G	T	patient 3	WXS	Illumina	NM_182762	exon5	c.C250A	p.L84I	411	0.129
RAPGEF5	hs37d5	7	22306590	22306590	synonymous_SNV	SNP	C	C	T	patient 3	WXS	Illumina	NM_012294	exon7	c.G330A	p.R110R	483	0.0663
AUTS2	hs37d5	7	70231206	70231212	nonframeshift_deletion	DEL	TCAGCAC	TCAGCAC	T	patient 3	WXS	Illumina	NM_015570	exon9	c.1576_1581del	p.526_527del	163	0.0245
RSBN1L	hs37d5	7	77326233	77326233	synonymous_SNV	SNP	C	C	T	patient 3	WXS	Illumina	NM_198467	exon1	c.C447T	p.A149A	243	0.0412
ATP5J2-PTCD1	hs37d5	7	99022798	99022798	nonsynonymous_SNV	SNP	C	C	T	patient 3	WXS	Illumina	NM_001198879	exon7	c.G1504A	p.V502I	94	0.2234
TRIM4	hs37d5	7	99490035	99490035	synonymous_SNV	SNP	C	C	T	patient 3	WXS	Illumina	NM_033017	exon7	c.G1254A	p.A418A	59	0.1017
FBXO24	hs37d5	7	100190541	100190541	nonsynonymous_SNV	SNP	G	G	A	patient 3	WXS	Illumina	NM_012172	exon5	c.G808A	p.G270R	119	0.0588
SLC12A9	hs37d5	7	100463595	100463595	nonsynonymous_SNV	SNP	G	G	C	patient 3	WXS	Illumina	NM_020246	exon14	c.G2113C	p.A705P	89	0.1573
MUC17	hs37d5	7	100682648	100682648	nonsynonymous_SNV	SNP	A	A	G	patient 3	WXS	Illumina	NM_001040105	exon3	c.A7951G	p.S2651G	185	0.0378
PAX4	hs37d5	7	127254550	127254550	nonsynonymous_SNV	SNP	C	C	T	patient 3	WXS	Illumina	NM_006193	exon3	c.G398A	p.R133Q	124	0.0484
RBM28	hs37d5	7	127958097	127958097	synonymous_SNV	SNP	G	G	A	patient 3	WXS	Illumina	NM_018077	exon15	c.C1626T	p.Y542Y	112	0.0536
GIMAP7	hs37d5	7	150217542	150217542	synonymous_SNV	SNP	C	C	T	patient 3	WXS	Illumina	NM_153236	exon2	c.C480T	p.C160C	94	0.0638
PTPRN2	hs37d5	7	157475500	157475500	nonsynonymous_SNV	SNP	G	G	A	patient 3	WXS	Illumina	NM_002847	exon13	c.C1918T	p.R640C	91	0.0659
PTPRN2	hs37d5	7	157997934	157997934	synonymous_SNV	SNP	A	A	G	patient 3	WXS	Illumina	NM_002847	exon4	c.T309C	p.Y103Y	114	0.2368
PCM1	hs37d5	8	17813051	17813069	nonframeshift_deletion	DEL	TCAATGGAGAATCCAATAG	TCAATGGAGAATCCAATAG	T	patient 3	WXS	Illumina	NM_006197	exon10	c.1362_1379del	p.454_460del	212	0.0236
NEFM	hs37d5	8	24775691	24775691	nonsynonymous_SNV	SNP	G	G	A	patient 3	WXS	Illumina	NM_005382	exon3	c.G2323A	p.E775K	154	0.0519
MRPL15	hs37d5	8	55047850	55047850	nonsynonymous_SNV	SNP	G	G	A	patient 3	WXS	Illumina	NM_014175	exon1	c.G7A	p.G3S	275	0.04
PSKH2	hs37d5	8	87081750	87081750	synonymous_SNV	SNP	A	A	T	patient 3	WXS	Illumina	NM_033126	exon1	c.T102A	p.P34P	23	0.3043
NBN	hs37d5	8	90967613	90967613	nonsynonymous_SNV	SNP	G	G	A	patient 3	WXS	Illumina	NM_002485	exon10	c.C1295T	p.S432L	291	0.0447
PKHD1L1	hs37d5	8	110535077	110535077	synonymous_SNV	SNP	G	G	A	patient 3	WXS	Illumina	NM_177531	exon75	c.G12288A	p.Q4096Q	89	0.0674
EPPK1	hs37d5	8	144942805	144942805	synonymous_SNV	SNP	G	G	A	patient 3	WXS	Illumina	NM_031308	exon1	c.C4617T	p.I1539I	34	0.2059
PLEC	hs37d5	8	144993246	144993246	synonymous_SNV	SNP	G	G	A	patient 3	WXS	Illumina	NM_201380	exon32	c.C11154T	p.G3718G	71	0.0845
KIAA0020	hs37d5	9	2824776	2824776	nonsynonymous_SNV	SNP	C	C	T	patient 3	WXS	Illumina	NM_014878	exon11	c.G1075A	p.A359T	71	0.0845
TTC39B	hs37d5	9	15307146	15307146	nonsynonymous_SNV	SNP	C	C	T	patient 3	WXS	Illumina	NM_152574	exon1	c.G176A	p.G59D	129	0.2558
TEK	hs37d5	9	27168554	27168554	synonymous_SNV	SNP	T	T	C	patient 3	WXS	Illumina	NM_000459	exon3	c.T426C	p.S142S	109	0.0642
TEX10	hs37d5	9	103072652	103072652	nonsynonymous_SNV	SNP	G	G	A	patient 3	WXS	Illumina	NM_017746	exon12	c.C2212T	p.H738Y	66	0.0909
DBC1	hs37d5	9	121930198	121930201	nonframeshift_deletion	DEL	GGAA	GGAA	G	patient 3	WXS	Illumina	NM_014618	exon8	c.1447_1449del	p.483_483del	49	0.0816
RC3H2	hs37d5	9	125645534	125645534	synonymous_SNV	SNP	T	T	C	patient 3	WXS	Illumina	NM_001100588	exon5	c.A708G	p.K236K	121	0.3058
ODF2	hs37d5	9	131245121	131245121	synonymous_SNV	SNP	G	G	A	patient 3	WXS	Illumina	NM_153435	exon10	c.G1134A	p.K378K	124	0.0726
NOTCH1	hs37d5	9	139391193	139391193	nonsynonymous_SNV	SNP	C	C	G	patient 3	WXS	Illumina	NM_017617	exon34	c.G6998C	p.G2333A	43	0.1163
FBXO18	hs37d5	10	5948004	5948004	nonsynonymous_SNV	SNP	G	G	T	patient 3	WXS	Illumina	NM_032807	exon4	c.G315T	p.Q105H	72	0.0694
CUBN	hs37d5	10	16911671	16911671	nonsynonymous_SNV	SNP	C	C	T	patient 3	WXS	Illumina	NM_001081	exon59	c.G9418A	p.A3140T	97	0.0722
ANKRD30A	hs37d5	10	37447327	37447327	synonymous_SNV	SNP	T	T	A	patient 3	WXS	Illumina	NM_052997	exon14	c.T1638A	p.P546P	132	0.0606
BMS1	hs37d5	10	43318597	43318597	nonsynonymous_SNV	SNP	A	A	G	patient 3	WXS	Illumina	NM_014753	exon20	c.A3164G	p.K1055R	230	0.0391
BMS1	hs37d5	10	43318606	43318606	nonsynonymous_SNV	SNP	G	G	A	patient 3	WXS	Illumina	NM_014753	exon20	c.G3173A	p.G1058D	206	0.1165
TACR2	hs37d5	10	71174698	71174703	nonframeshift_deletion	DEL	TACAGG	TACAGG	T	patient 3	WXS	Illumina	NM_001057	exon2	c.585_587del	p.195_196del	88	0.0341
GHITM	hs37d5	10	85903787	85903787	nonsynonymous_SNV	SNP	C	C	G	patient 3	WXS	Illumina	NM_014394	exon4	c.C266G	p.A89G	189	0.037
TM9SF3	hs37d5	10	98292941	98292941	nonsynonymous_SNV	SNP	C	C	T	patient 3	WXS	Illumina	NM_020123	exon10	c.G1192A	p.V398I	111	0.0541
HABP2	hs37d5	10	115336949	115336949	synonymous_SNV	SNP	A	A	G	patient 3	WXS	Illumina	NM_004132	exon5	c.A372G	p.Q124Q	113	0.2743
DPYSL4	hs37d5	10	134013862	134013862	nonsynonymous_SNV	SNP	G	G	T	patient 3	WXS	Illumina	NM_006426	exon9	c.G814T	p.V272L	122	0.082
MUC2	hs37d5	11	1092526	1092526	nonsynonymous_SNV	SNP	C	C	T	patient 3	WXS	Illumina	NM_002457	exon30	c.C4345T	p.P1449S	118	0.0678
MUC2	hs37d5	11	1093559	1093559	nonsynonymous_SNV	SNP	T	T	C	patient 3	WXS	Illumina	NM_002457	exon30	c.T5378C	p.I1793T	149	0.047
NUP98	hs37d5	11	3697487	3697487	nonsynonymous_SNV	SNP	G	G	T	patient 3	WXS	Illumina	NM_016320	exon33	c.C5305A	p.H1769N	44	0.1364
STK33	hs37d5	11	8435092	8435092	nonsynonymous_SNV	SNP	C	C	T	patient 3	WXS	Illumina	NM_030906	exon13	c.G1294A	p.G432R	119	0.0588
NAV2	hs37d5	11	20127228	20127228	nonsynonymous_SNV	SNP	C	C	T	patient 3	WXS	Illumina	NM_001244963	exon37	c.C6973T	p.L2325F	62	0.1613
QSER1	hs37d5	11	32956428	32956428	synonymous_SNV	SNP	A	A	G	patient 3	WXS	Illumina	NM_001076786	exon4	c.A3237G	p.K1079K	65	0.0769
ABTB2	hs37d5	11	34174119	34174119	nonsynonymous_SNV	SNP	G	G	A	patient 3	WXS	Illumina	NM_145804	exon17	c.C2893T	p.P965S	18	0.2222
AMBRA1	hs37d5	11	46439468	46439468	synonymous_SNV	SNP	T	T	C	patient 3	WXS	Illumina	NM_017749	exon16	c.A2841G	p.R947R	135	0.0444
LRP4	hs37d5	11	46898883	46898883	synonymous_SNV	SNP	G	G	A	patient 3	WXS	Illumina	NM_002334	exon23	c.C3144T	p.N1048N	89	0.0674
OR4C16	hs37d5	11	55340224	55340224	synonymous_SNV	SNP	G	G	A	patient 3	WXS	Illumina	NM_001004701	exon1	c.G621A	p.V207V	124	0.0565
MS4A5	hs37d5	11	60197232	60197232	nonsynonymous_SNV	SNP	T	T	C	patient 3	WXS	Illumina	NM_023945	exon1	c.T85C	p.S29P	79	0.2278
ATL3	hs37d5	11	63414006	63414006	nonsynonymous_SNV	SNP	C	C	A	patient 3	WXS	Illumina	NM_015459	exon6	c.G591T	p.M197I	238	0.0378
KRTAP5-8	hs37d5	11	71249169	71249169	nonsynonymous_SNV	SNP	G	G	A	patient 3	WXS	Illumina	NM_021046	exon1	c.G68A	p.G23E	130	0.0615
KRTAP5-10	hs37d5	11	71277125	71277125	synonymous_SNV	SNP	T	T	C	patient 3	WXS	Illumina	NM_001012710	exon1	c.T492C	p.N164N	148	0.0541
TYR	hs37d5	11	88911695	88911695	nonsynonymous_SNV	SNP	T	T	G	patient 3	WXS	Illumina	NM_000372	exon1	c.T574G	p.S192A	121	0.0496
NAALAD2	hs37d5	11	89882205	89882205	nonsynonymous_SNV	SNP	C	C	A	patient 3	WXS	Illumina	NM_005467	exon4	c.C413A	p.P138Q	122	0.0984
ENDOD1	hs37d5	11	94862469	94862469	nonsynonymous_SNV	SNP	G	G	A	patient 3	WXS	Illumina	NM_015036	exon2	c.G1229A	p.R410K	53	0.0943
BCO2	hs37d5	11	112085537	112085537	nonsynonymous_SNV	SNP	G	G	T	patient 3	WXS	Illumina	NM_031938	exon10	c.G1385T	p.G462V	93	0.3763
AEBP2	hs37d5	12	19615478	19615478	nonsynonymous_SNV	SNP	A	A	G	patient 3	WXS	Illumina	NM_001114176	exon2	c.A706G	p.I236V	122	0.0492
ACVRL1	hs37d5	12	52309854	52309854	synonymous_SNV	SNP	C	C	T	patient 3	WXS	Illumina	NM_001077401	exon7	c.C1083T	p.Y361Y	41	0.122
KRT76	hs37d5	12	53170690	53170690	nonsynonymous_SNV	SNP	C	C	T	patient 3	WXS	Illumina	NM_015848	exon1	c.G386A	p.G129D	135	0.0519
BAZ2A	hs37d5	12	56999092	56999092	nonsynonymous_SNV	SNP	C	C	T	patient 3	WXS	Illumina	NM_013449	exon14	c.G2519A	p.R840Q	136	0.0515
SH2B3	hs37d5	12	111884756	111884756	nonsynonymous_SNV	SNP	G	G	A	patient 3	WXS	Illumina	NM_005475	exon4	c.G845A	p.R282Q	66	0.0758
C12orf51	hs37d5	12	112721066	112721066	nonsynonymous_SNV	SNP	C	C	T	patient 3	WXS	Illumina	NM_001109662	exon8	c.G944A	p.R315H	131	0.0611
SPPL3	hs37d5	12	121205353	121205353	nonsynonymous_SNV	SNP	C	C	T	patient 3	WXS	Illumina	NM_139015	exon9	c.G817A	p.V273I	70	0.0714
GPR133	hs37d5	12	131471758	131471758	synonymous_SNV	SNP	G	G	A	patient 3	WXS	Illumina	NM_198827	exon6	c.G609A	p.E203E	97	0.0825
GALNT9	hs37d5	12	132685768	132685768	synonymous_SNV	SNP	C	C	T	patient 3	WXS	Illumina	NM_001122636	exon8	c.G1302A	p.L434L	93	0.0645
CENPJ	hs37d5	13	25486152	25486152	nonsynonymous_SNV	SNP	T	T	C	patient 3	WXS	Illumina	NM_018451	exon3	c.A500G	p.Q167R	221	0.2262
PDS5B	hs37d5	13	33284233	33284233	nonsynonymous_SNV	SNP	C	C	T	patient 3	WXS	Illumina	NM_015032	exon19	c.C2113T	p.H705Y	161	0.0497
TBC1D4	hs37d5	13	75887024	75887024	nonsynonymous_SNV	SNP	C	C	T	patient 3	WXS	Illumina	NM_014832	exon13	c.G2233A	p.G745R	123	0.0569
DCT	hs37d5	13	95095815	95095815	nonsynonymous_SNV	SNP	T	T	C	patient 3	WXS	Illumina	NM_001129889	exon9	c.A1355G	p.Q452R	133	0.1504
ANKRD10	hs37d5	13	111532434	111532434	synonymous_SNV	SNP	C	C	T	patient 3	WXS	Illumina	NM_017664	exon6	c.G813A	p.S271S	114	0.0614
TEP1	hs37d5	14	20863690	20863690	nonsynonymous_SNV	SNP	C	C	T	patient 3	WXS	Illumina	NM_007110	exon12	c.G1847A	p.R616H	110	0.1455
HECTD1	hs37d5	14	31575898	31575898	nonsynonymous_SNV	SNP	T	T	C	patient 3	WXS	Illumina	NM_015382	exon38	c.A7180G	p.K2394E	125	0.088
HECTD1	hs37d5	14	31609180	31609180	nonsynonymous_SNV	SNP	C	C	T	patient 3	WXS	Illumina	NM_015382	exon19	c.G2897A	p.R966Q	109	0.055
HECTD1	hs37d5	14	31642762	31642762	nonsynonymous_SNV	SNP	G	G	A	patient 3	WXS	Illumina	NM_015382	exon5	c.C854T	p.T285I	37	0.3243
PCNX	hs37d5	14	71500715	71500715	nonsynonymous_SNV	SNP	G	G	C	patient 3	WXS	Illumina	NM_014982	exon18	c.G3736C	p.V1246L	173	0.0347
C14orf43	hs37d5	14	74205867	74205867	stopgain_SNV	SNP	G	G	T	patient 3	WXS	Illumina	NM_001043318	exon2	c.C845A	p.S282X	75	0.08
ISCA2	hs37d5	14	74961589	74961589	nonsynonymous_SNV	SNP	A	A	C	patient 3	WXS	Illumina	NM_194279	exon4	c.A351C	p.K117N	126	0.0476
TTLL5	hs37d5	14	76149896	76149896	nonsynonymous_SNV	SNP	C	C	T	patient 3	WXS	Illumina	NM_015072	exon5	c.C268T	p.H90Y	92	0.4457
IFT43	hs37d5	14	76549015	76549015	nonsynonymous_SNV	SNP	T	T	C	patient 3	WXS	Illumina	NM_052873	exon6	c.T439C	p.Y147H	48	0.0833
TDP1	hs37d5	14	90429870	90429870	nonsynonymous_SNV	SNP	C	C	A	patient 3	WXS	Illumina	NM_018319	exon3	c.C412A	p.L138I	53	0.1132
HERC2	hs37d5	15	28463794	28463794	synonymous_SNV	SNP	T	T	G	patient 3	WXS	Illumina	NM_004667	exon38	c.C5869C	p.H1957H	121	0.0579
TJP1	hs37d5	15	30058658	30058658	nonsynonymous_SNV	SNP	C	C	T	patient 3	WXS	Illumina	NM_003257	exon5	c.G400A	p.E134K	74	0.1081
RAB27A	hs37d5	15	55497711	55497711	synonymous_SNV	SNP	G	G	A	patient 3	WXS	Illumina	NM_183234	exon7	c.C660T	p.G220G	146	0.0548
VPS13C	hs37d5	15	62257013	62257013	synonymous_SNV	SNP	G	G	A	patient 3	WXS	Illumina	NM_020821	exon31	c.C3099T	p.V1033V	133	0.0526
HCN4	hs37d5	15	73624598	73624598	synonymous_SNV	SNP	C	C	A	patient 3	WXS	Illumina	NM_005477	exon3	c.G1245T	p.V415V	58	0.0862
C15orf59	hs37d5	15	74032325	74032325	stopgain_SNV	SNP	G	G	T	patient 3	WXS	Illumina	NM_001039614	exon2	c.C815A	p.S272X	163	0.0491
PPCDC	hs37d5	15	75335864	75335864	nonsynonymous_SNV	SNP	C	C	T	patient 3	WXS	Illumina	NM_021823	exon3	c.C218T	p.A73V	89	0.2697
WFIKKN1	hs37d5	16	682808	682808	nonsynonymous_SNV	SNP	T	T	C	patient 3	WXS	Illumina	NM_053284	exon2	c.T398C	p.L133P	38	0.1053
PDXDC1	hs37d5	16	15102664	15102664	nonsynonymous_SNV	SNP	G	G	A	patient 3	WXS	Illumina	NM_015027	exon7	c.G608A	p.R203H	307	0.0326
ZNF689	hs37d5	16	30616627	30616627	nonsynonymous_SNV	SNP	C	C	G	patient 3	WXS	Illumina	NM_138447	exon3	c.G461C	p.C154S	115	0.0522
BCL7C	hs37d5	16	30899237	30899237	synonymous_SNV	SNP	C	C	T	patient 3	WXS	Illumina	NM_004765	exon6	c.G603A	p.S201S	156	0.1154
BCL7C	hs37d5	16	30899271	30899271	nonsynonymous_SNV	SNP	A	A	C	patient 3	WXS	Illumina	NM_004765	exon6	c.T569G	p.V190G	129	0.0543
ELMO3	hs37d5	16	67235729	67235729	nonsynonymous_SNV	SNP	C	C	T	patient 3	WXS	Illumina	NM_024712	exon11	c.C1162T	p.R388C	124	0.0565
CDH3	hs37d5	16	68718543	68718543	nonsynonymous_SNV	SNP	G	G	A	patient 3	WXS	Illumina	NM_001793	exon10	c.G1240A	p.A414T	192	0.1302
KIAA0513	hs37d5	16	85105434	85105434	synonymous_SNV	SNP	C	C	T	patient 3	WXS	Illumina	NM_014732	exon3	c.C375T	p.C125C	153	0.0588
PIEZO1	hs37d5	16	88800863	88800863	nonsynonymous_SNV	SNP	C	C	A	patient 3	WXS	Illumina	NM_001142864	exon16	c.G2081T	p.C694F	42	0.119
ITGAE	hs37d5	17	3646828	3646828	synonymous_SNV	SNP	G	G	A	patient 3	WXS	Illumina	NM_002208	exon19	c.C2403T	p.P801P	120	0.1083
CHRNE	hs37d5	17	4802074	4802074	nonsynonymous_SNV	SNP	T	T	C	patient 3	WXS	Illumina	NM_000080	exon12	c.A1439G	p.N480S	66	0.1818
ZNF232	hs37d5	17	5013029	5013029	nonsynonymous_SNV	SNP	C	C	T	patient 3	WXS	Illumina	NM_014519	exon3	c.G158A	p.G53E	106	0.0566
AIPL1	hs37d5	17	6328826	6328826	nonsynonymous_SNV	SNP	G	G	A	patient 3	WXS	Illumina	NM_014336	exon6	c.C1109T	p.P370L	155	0.0645
DRG2	hs37d5	17	18010487	18010487	nonsynonymous_SNV	SNP	G	G	A	patient 3	WXS	Illumina	NM_001388	exon13	c.G1028A	p.S343N	81	0.3704
ALKBH5	hs37d5	17	18110149	18110149	nonsynonymous_SNV	SNP	G	G	A	patient 3	WXS	Illumina	NM_017758	exon3	c.G872A	p.R291Q	69	0.0725
ATAD5	hs37d5	17	29162870	29162870	nonsynonymous_SNV	SNP	C	C	T	patient 3	WXS	Illumina	NM_024857	exon2	c.C1771T	p.P591S	62	0.5
NF1	hs37d5	17	29422330	29422330	nonsynonymous_SNV	SNP	G	G	T	patient 3	WXS	Illumina	NM_001042492	exon1	c.G3T	p.M1I	25	0.96
SUZ12	hs37d5	17	30310070	30310070	stopgain_SNV	SNP	G	G	T	patient 3	WXS	Illumina	NM_015355	exon9	c.G970T	p.E324X	82	0.8171
CCL23	hs37d5	17	34340321	34340321	synonymous_SNV	SNP	G	G	A	patient 3	WXS	Illumina	NM_005064	exon4	c.C330T	p.F110F	82	0.061
KRT24	hs37d5	17	38856324	38856324	nonsynonymous_SNV	SNP	C	C	A	patient 3	WXS	Illumina	NM_019016	exon5	c.G1069T	p.A357S	153	0.0523
KRTAP4-7	hs37d5	17	39240795	39240795	nonsynonymous_SNV	SNP	A	A	T	patient 3	WXS	Illumina	NM_033061	exon1	c.A337T	p.S113C	80	0.0875
KRTAP4-8	hs37d5	17	39254184	39254184	nonsynonymous_SNV	SNP	T	T	A	patient 3	WXS	Illumina	NM_031960	exon1	c.A153T	p.R51S	90	0.0667
KRTAP4-8	hs37d5	17	39254186	39254186	stopgain_SNV	SNP	T	T	A	patient 3	WXS	Illumina	NM_031960	exon1	c.A151T	p.R51X	91	0.0659
KRTAP4-8	hs37d5	17	39254196	39254196	synonymous_SNV	SNP	G	G	A	patient 3	WXS	Illumina	NM_031960	exon1	c.C141T	p.S47S	97	0.0619
KRTAP4-11	hs37d5	17	39274422	39274422	nonsynonymous_SNV	SNP	C	C	T	patient 3	WXS	Illumina	NM_033059	exon1	c.G146A	p.C49Y	164	0.0671
KRTAP9-9	hs37d5	17	39411895	39411895	synonymous_SNV	SNP	T	T	C	patient 3	WXS	Illumina	NM_030975	exon1	c.T258C	p.C86C	108	0.0741
KRT9	hs37d5	17	39727892	39727892	nonsynonymous_SNV	SNP	C	C	T	patient 3	WXS	Illumina	NM_000226	exon1	c.G353A	p.G118D	142	0.0563
MPP2	hs37d5	17	41960327	41960327	nonsynonymous_SNV	SNP	C	C	T	patient 3	WXS	Illumina	NM_005374	exon5	c.G397A	p.V133I	48	0.125
VEZF1	hs37d5	17	56056604	56056604	synonymous_SNV	SNP	T	T	C	patient 3	WXS	Illumina	NM_007146	exon5	c.A1047G	p.Q349Q	155	0.0387
EVPL	hs37d5	17	74004773	74004773	nonsynonymous_SNV	SNP	G	G	T	patient 3	WXS	Illumina	NM_001988	exon22	c.C4513A	p.Q1505K	77	0.0909
EVPL	hs37d5	17	74004976	74004976	nonsynonymous_SNV	SNP	C	C	A	patient 3	WXS	Illumina	NM_001988	exon22	c.G4310T	p.R1437M	73	0.0822
UBE2O	hs37d5	17	74388132	74388132	synonymous_SNV	SNP	C	C	T	patient 3	WXS	Illumina	NM_022066	exon16	c.G3009A	p.L1003L	135	0.0963
SLC26A11	hs37d5	17	78195463	78195463	nonsynonymous_SNV	SNP	C	C	T	patient 3	WXS	Illumina	NM_173626	exon3	c.C104T	p.P35L	89	0.0674
BAIAP2	hs37d5	17	79079920	79079920	nonsynonymous_SNV	SNP	C	C	A	patient 3	WXS	Illumina	NM_017451	exon11	c.C1311A	p.S437R	75	0.0667
ESCO1	hs37d5	18	19116009	19116009	synonymous_SNV	SNP	G	G	T	patient 3	WXS	Illumina	NM_052911	exon10	c.C2181A	p.I727I	140	0.5429
ESCO1	hs37d5	18	19116026	19116026	synonymous_SNV	SNP	G	G	A	patient 3	WXS	Illumina	NM_052911	exon10	c.C2164T	p.L722L	158	0.5127
SS18	hs37d5	18	23637557	23637557	nonsynonymous_SNV	SNP	C	C	T	patient 3	WXS	Illumina	NM_001007559	exon4	c.G381A	p.M127I	91	0.0879
CELF4	hs37d5	18	34833810	34833810	nonsynonymous_SNV	SNP	C	C	G	patient 3	WXS	Illumina	NM_020180	exon12	c.G1425C	p.Q475H	59	0.1017
SLC14A2	hs37d5	18	43219725	43219725	synonymous_SNV	SNP	C	C	A	patient 3	WXS	Illumina	NM_001242692	exon8	c.C858A	p.I286I	87	0.092
CXXC1	hs37d5	18	47811555	47811555	synonymous_SNV	SNP	C	C	T	patient 3	WXS	Illumina	NM_001101654	exon7	c.G729A	p.Q243Q	73	0.0822
SMAD4	hs37d5	18	48575137	48575137	nonsynonymous_SNV	SNP	C	C	T	patient 3	WXS	Illumina	NM_005359	exon3	c.C331T	p.H111Y	120	0.0667
PHLPP1	hs37d5	18	60646125	60646125	nonsynonymous_SNV	SNP	G	G	A	patient 3	WXS	Illumina	NM_194449	exon17	c.G4615A	p.A1539T	79	0.0633
ZNF77	hs37d5	19	2939399	2939399	nonsynonymous_SNV	SNP	C	C	T	patient 3	WXS	Illumina	NM_021217	exon2	c.G10A	p.V4M	117	0.1538
ZNF431	hs37d5	19	21366342	21366342	synonymous_SNV	SNP	C	C	T	patient 3	WXS	Illumina	NM_133473	exon5	c.C1236T	p.S412S	169	0.071
RHPN2	hs37d5	19	33482729	33482729	splicing	SNP	C	C	A	patient 3	WXS	Illumina	0	0	0	0	105	0.0571
ZNF345	hs37d5	19	37367739	37367739	nonsynonymous_SNV	SNP	A	A	G	patient 3	WXS	Illumina	NM_001242472	exon3	c.A7G	p.N3D	93	0.2151
SIPA1L3	hs37d5	19	38632034	38632034	synonymous_SNV	SNP	C	C	T	patient 3	WXS	Illumina	NM_015073	exon11	c.C3354T	p.P1118P	116	0.0517
FBL	hs37d5	19	40329737	40329737	nonsynonymous_SNV	SNP	C	C	T	patient 3	WXS	Illumina	NM_001436	exon5	c.G487A	p.V163I	89	0.2135
SHKBP1	hs37d5	19	41089329	41089329	nonsynonymous_SNV	SNP	G	G	T	patient 3	WXS	Illumina	NM_138392	exon11	c.G986T	p.S329I	138	0.0507
TPRX1	hs37d5	19	48305505	48305505	nonsynonymous_SNV	SNP	T	T	G	patient 3	WXS	Illumina	NM_198479	exon2	c.A763C	p.I255L	107	0.0561
FUT1	hs37d5	19	49254097	49254097	nonsynonymous_SNV	SNP	C	C	T	patient 3	WXS	Illumina	NM_000148	exon4	c.G442A	p.D148N	111	0.0811
PRR12	hs37d5	19	50128174	50128174	nonsynonymous_SNV	SNP	G	G	A	patient 3	WXS	Illumina	NM_020719	exon12	c.G5795A	p.R1932Q	50	0.1
CTU1	hs37d5	19	51607824	51607824	nonsynonymous_SNV	SNP	C	C	A	patient 3	WXS	Illumina	NM_145232	exon2	c.G3T	p.M1I	97	0.0619
ZNF28	hs37d5	19	53304573	53304573	synonymous_SNV	SNP	T	T	C	patient 3	WXS	Illumina	NM_006969	exon4	c.A525G	p.S175S	143	0.3007
CNOT3	hs37d5	19	54649673	54649673	nonsynonymous_SNV	SNP	C	C	A	patient 3	WXS	Illumina	NM_014516	exon9	c.C731A	p.P244H	29	0.2759
PPP1R12C	hs37d5	19	55606910	55606910	nonsynonymous_SNV	SNP	G	G	A	patient 3	WXS	Illumina	NM_017607	exon10	c.C1289T	p.A430V	76	0.0789
DUXA	hs37d5	19	57669820	57669820	nonsynonymous_SNV	SNP	C	C	T	patient 3	WXS	Illumina	NM_001012729	exon4	c.G314A	p.R105H	66	0.0758
ZNF814	hs37d5	19	58385748	58385748	nonsynonymous_SNV	SNP	G	G	A	patient 3	WXS	Illumina	NM_001144989	exon3	c.C1010T	p.A337V	178	0.2416
ZNF814	hs37d5	19	58385762	58385762	synonymous_SNV	SNP	C	C	G	patient 3	WXS	Illumina	NM_001144989	exon3	c.G996C	p.S332S	139	0.1727

NRSN2	hs37d5	20	333899	333899	nonsynonymous_SNV	SNP	G	G	A	patient 3	WXS	Illumina	NM_024958	exon4	c.G235A	p.A79T	106	0.066
PTPRA	hs37d5	20	3001989	3001989	nonsynonymous_SNV	SNP	G	G	A	patient 3	WXS	Illumina	NM_002836	exon18	c.G1076A	p.C359Y	83	0.0723
XRN2	hs37d5	20	21367601	21367601	nonsynonymous_SNV	SNP	C	C	A	patient 3	WXS	Illumina	NM_012255	exon29	c.C2744A	p.P915H	63	0.0794
ACTR5	hs37d5	20	37377255	37377255	nonsynonymous_SNV	SNP	G	G	A	patient 3	WXS	Illumina	NM_024855	exon1	c.G134A	p.G45D	36	0.1389
CDH4	hs37d5	20	60504711	60504711	nonsynonymous_SNV	SNP	G	G	A	patient 3	WXS	Illumina	NM_001794	exon13	c.G2050A	p.G684R	67	0.0746
LAMA5	hs37d5	20	60892554	60892554	splicing	SNP	T	T	C	patient 3	WXS	Illumina	0	0	0	0	55	0.1091
KCNQ2	hs37d5	20	62045501	62045501	nonsynonymous_SNV	SNP	G	G	A	patient 3	WXS	Illumina	NM_172107	exon14	c.C1571T	p.P524L	112	0.0536
MORC3	hs37d5	21	37741629	37741629	nonsynonymous_SNV	SNP	G	G	A	patient 3	WXS	Illumina	NM_015358	exon15	c.G1963A	p.V655I	123	0.065
CLDN14	hs37d5	21	37833792	37833792	stopgain_SNV	SNP	G	G	A	patient 3	WXS	Illumina	NM_001146077	exon3	c.C202T	p.R68X	70	0.0714
ABCG1	hs37d5	21	43645932	43645932	nonsynonymous_SNV	SNP	G	G	A	patient 3	WXS	Illumina	NM_004915	exon2	c.G194A	p.R65H	123	0.0488
COL6A1	hs37d5	21	47418870	47418870	synonymous_SNV	SNP	C	C	T	patient 3	WXS	Illumina	NM_001848	exon25	c.C1671T	p.D557D	160	0.0437
RIMBP3	hs37d5	22	20456608	20456608	nonsynonymous_SNV	SNP	C	C	G	patient 3	WXS	Illumina	NM_015672	exon1	c.G4694C	p.R1565T	12	0.6667
RIMBP3	hs37d5	22	20457808	20457808	nonsynonymous_SNV	SNP	C	C	G	patient 3	WXS	Illumina	NM_015672	exon1	c.G3494C	p.R1165T	115	0.2261
RIMBP3	hs37d5	22	20458425	20458425	nonsynonymous_SNV	SNP	C	C	G	patient 3	WXS	Illumina	NM_015672	exon1	c.G2877C	p.E959D	120	0.2333
BCR	hs37d5	22	23632531	23632531	stopgain_SNV	SNP	G	G	T	patient 3	WXS	Illumina	NM_004327	exon14	c.G2713T	p.E905X	124	0.1371
CABIN1	hs37d5	22	24563078	24563078	nonsynonymous_SNV	SNP	A	A	G	patient 3	WXS	Illumina	NM_012295	exon32	c.A5479G	p.T1827A	11	0.5455
CHEK2	hs37d5	22	29091840	29091840	nonsynonymous_SNV	SNP	T	T	C	patient 3	WXS	Illumina	NM_001005735	exon12	c.A1246G	p.K416E	129	0.0465
PLA2G6	hs37d5	22	38522425	38522425	synonymous_SNV	SNP	G	G	A	patient 3	WXS	Illumina	NM_003560	exon10	c.C1380T	p.A460A	19	0.2105
CDKL5	hs37d5	X	18600039	18600039	nonsynonymous_SNV	SNP	C	C	A	patient 3	WXS	Illumina	NM_003159	exon7	c.C432A	p.S144R	138	0.0507
DMD	hs37d5	X	31165494	31165494	synonymous_SNV	SNP	C	C	T	patient 3	WXS	Illumina	NM_004006	exon75	c.G10695A	p.L3565L	84	0.4405
PRRG1	hs37d5	X	37312579	37312579	nonsynonymous_SNV	SNP	C	C	G	patient 3	WXS	Illumina	NM_001142395	exon4	c.C362G	p.P121R	113	0.0708
IQSEC2	hs37d5	X	53283764	53283764	nonsynonymous_SNV	SNP	G	G	A	patient 3	WXS	Illumina	NM_001111125	exon4	c.C1349T	p.S450F	65	0.1077
IL2RG	hs37d5	X	70327618	70327618	nonsynonymous_SNV	SNP	G	G	T	patient 3	WXS	Illumina	NM_000206	exon8	c.C1078A	p.P360T	109	0.0642
PHKA1	hs37d5	X	71846821	71846821	splicing	SNP	C	C	T	patient 3	WXS	Illumina	0	0	0	0	102	0.0686
ATP7A	hs37d5	X	77286904	77286904	nonsynonymous_SNV	SNP	G	G	A	patient 3	WXS	Illumina	NM_000052	exon16	c.G3118A	p.V1040I	125	0.072
AMOT	hs37d5	X	112024289	112024289	nonsynonymous_SNV	SNP	C	C	G	patient 3	WXS	Illumina	NM_001113490	exon9	c.G2298C	p.Q766H	46	0.087
IGSF1	hs37d5	X	130407836	130407836	synonymous_SNV	SNP	G	G	T	patient 3	WXS	Illumina	NM_001170961	exon20	c.C3960A	p.G1320G	38	0.4211
ACAP3	hs37d5	1	1231203	1231203	nonsynonymous_SNV	SNP	G	G	A	patient 4	WXS	Illumina	NM_030649	exon18	c.C1618T	p.R540W	44	0.1136
PRAMEF1	hs37d5	1	12855758	12855758	synonymous_SNV	SNP	A	A	G	patient 4	WXS	Illumina	NM_023013	exon4	c.A1038G	p.K346K	163	0.0675
PRAMEF4	hs37d5	1	12939495	12939495	nonsynonymous_SNV	SNP	A	A	T	patient 4	WXS	Illumina	NM_001009611	exon4	c.T1307A	p.I436N	118	0.0763
PRAMEF4	hs37d5	1	12939535	12939535	nonsynonymous_SNV	SNP	C	C	T	patient 4	WXS	Illumina	NM_001009611	exon4	c.G1267A	p.G423S	196	0.0918
ST6GALNAC3	hs37d5	1	76779563	76779563	nonsynonymous_SNV	SNP	T	T	C	patient 4	WXS	Illumina	NM_152996	exon2	c.T92C	p.V31A	142	0.2324
GBP7	hs37d5	1	89599037	89599037	synonymous_SNV	SNP	A	A	G	patient 4	WXS	Illumina	NM_207398	exon10	c.T1566C	p.S522S	271	0.0443
PPIAL4G	hs37d5	1	143767628	143767628	nonsynonymous_SNV	SNP	C	C	T	patient 4	WXS	Illumina	NM_001123068	exon1	c.G221A	p.G74D	297	0.0471
NBPF10	hs37d5	1	145293469	145293469	nonsynonymous_SNV	SNP	G	G	C	patient 4	WXS	Illumina	NM_001039703	exon1	c.G64C	p.E22Q	1656	0.0187
NBPF10	hs37d5	1	145365372	145365372	nonsynonymous_SNV	SNP	C	C	G	patient 4	WXS	Illumina	NM_001039703	exon80	c.C9997G	p.L3333V	92	0.1957
NBPF10	hs37d5	1	145368664	145368664	stopgain_SNV	SNP	G	G	T	patient 4	WXS	Illumina	NM_001039703	exon84	c.G10642T	p.G3548X	709	0.0296
NBPF14	hs37d5	1	148009510	148009510	synonymous_SNV	SNP	G	G	A	patient 4	WXS	Illumina	NM_015383	exon16	c.C1797T	p.S599S	343	0.0991
HRNR	hs37d5	1	152187589	152187589	nonsynonymous_SNV	SNP	G	G	T	patient 4	WXS	Illumina	NM_001009931	exon3	c.C6516A	p.H2172Q	375	0.0453
NDUFS2	hs37d5	1	161172245	161172245	nonsynonymous_SNV	SNP	G	G	C	patient 4	WXS	Illumina	NM_004550	exon2	c.G70C	p.V24L	167	0.0719
CNTN2	hs37d5	1	205041245	205041245	synonymous_SNV	SNP	C	C	A	patient 4	WXS	Illumina	NM_005076	exon20	c.C2715A	p.A905A	144	0.2153
CR1	hs37d5	1	207787753	207787753	stopgain_SNV	SNP	C	C	T	patient 4	WXS	Illumina	NM_000651	exon40	c.C6580T	p.R2194X	304	0.0691
TRAF3IP3	hs37d5	1	209948734	209948734	nonsynonymous_SNV	SNP	T	T	C	patient 4	WXS	Illumina	NM_025228	exon10	c.T815C	p.M272T	80	0.2
CDKL4	hs37d5	2	39414801	39414801	synonymous_SNV	SNP	G	G	A	patient 4	WXS	Illumina	NM_001009565	exon6	c.C702T	p.F234F	151	0.2914
SSB	hs37d5	2	170668259	170668259	nonsynonymous_SNV	SNP	A	A	T	patient 4	WXS	Illumina	NM_003142	exon12	c.A1220T	p.D407V	133	0.5113
PRR21	hs37d5	2	240982243	240982243	nonsynonymous_SNV	SNP	G	G	A	patient 4	WXS	Illumina	NM_001080835	exon1	c.C157T	p.R53W	150	0.0667
SMARCC1	hs37d5	3	47770539	47770539	nonsynonymous_SNV	SNP	G	G	T	patient 4	WXS	Illumina	NM_003074	exon6	c.C622A	p.P208T	146	0.2603
OR5AC2	hs37d5	3	97806149	97806149	nonsynonymous_SNV	SNP	C	C	A	patient 4	WXS	Illumina	NM_054106	exon1	c.C133A	p.L45I	271	0.059
CLSTN2	hs37d5	3	140281145	140281145	nonsynonymous_SNV	SNP	T	T	C	patient 4	WXS	Illumina	NM_022131	exon13	c.T2207C	p.I736T	105	0.2762
MME	hs37d5	3	154801997	154801997	nonsynonymous_SNV	SNP	A	A	G	patient 4	WXS	Illumina	NM_000902	exon2	c.A41G	p.N14S	125	0.256
CRIPAK	hs37d5	4	1389037	1389037	nonsynonymous_SNV	SNP	G	G	A	patient 4	WXS	Illumina	NM_175918	exon1	c.G738A	p.M246I	87	0.1264
DRD5	hs37d5	4	9783971	9783971	synonymous_SNV	SNP	C	C	T	patient 4	WXS	Illumina	NM_000798	exon1	c.C318T	p.Y106Y	87	0.0805
UGT2B11	hs37d5	4	70066424	70066424	nonsynonymous_SNV	SNP	T	T	C	patient 4	WXS	Illumina	NM_001073	exon6	c.A1324G	p.I442V	430	0.0512
ANKRD33B	hs37d5	5	10649746	10649746	nonsynonymous_SNV	SNP	G	G	A	patient 4	WXS	Illumina	NM_001164440	exon4	c.G1006A	p.G336R	126	0.0714
DND1	hs37d5	5	140050907	140050907	nonsynonymous_SNV	SNP	C	C	T	patient 4	WXS	Illumina	NM_194249	exon4	c.G1033A	p.A345T	132	0.0682
DND1	hs37d5	5	140050926	140050926	synonymous_SNV	SNP	G	G	A	patient 4	WXS	Illumina	NM_194249	exon4	c.C1014T	p.N338N	142	0.0775
PCDHB13	hs37d5	5	140594442	140594442	synonymous_SNV	SNP	A	A	G	patient 4	WXS	Illumina	NM_018933	exon1	c.A747G	p.R249R	172	0.0407
HLA-DRB5	hs37d5	6	32497913	32497913	nonsynonymous_SNV	SNP	C	C	A	patient 4	WXS	Illumina	NM_002125	exon1	c.G89T	p.G30V	441	0.0522
PRIM2	hs37d5	6	57393112	57393112	splicing	SNP	T	T	C	patient 4	WXS	Illumina	0	0	0	0	319	0.0345
PPIL4	hs37d5	6	149862538	149862538	synonymous_SNV	SNP	A	A	C	patient 4	WXS	Illumina	NM_139126	exon3	c.T168G	p.P56P	250	0.116
AGMO	hs37d5	7	15425158	15425158	synonymous_SNV	SNP	T	T	A	patient 4	WXS	Illumina	NM_001004320	exon10	c.A987T	p.S329S	269	0.1524
STYXL1	hs37d5	7	75643121	75643121	nonsynonymous_SNV	SNP	T	T	C	patient 4	WXS	Illumina	NM_016086	exon5	c.A392G	p.Y131C	68	0.2794
MUC12	hs37d5	7	100637639	100637639	synonymous_SNV	SNP	C	C	T	patient 4	WXS	Illumina	NM_001164462	exon2	c.C3795T	p.L1265L	11	0.3636
MUC12	hs37d5	7	100638809	100638809	synonymous_SNV	SNP	C	C	A	patient 4	WXS	Illumina	NM_001164462	exon2	c.C4965A	p.G1655G	165	0.0727
TFEC	hs37d5	7	115581955	115581955	nonsynonymous_SNV	SNP	G	G	C	patient 4	WXS	Illumina	NM_012252	exon7	c.C655G	p.R219G	185	0.4757
TMEM209	hs37d5	7	129813693	129813693	synonymous_SNV	SNP	C	C	T	patient 4	WXS	Illumina	NM_032842	exon12	c.G1431A	p.Q477Q	231	0.2035
MLL3	hs37d5	7	151962168	151962168	nonsynonymous_SNV	SNP	C	C	A	patient 4	WXS	Illumina	NM_170606	exon8	c.G1139T	p.R380L	744	0.0403
PABPC1	hs37d5	8	101717267	101717267	nonsynonymous_SNV	SNP	G	G	A	patient 4	WXS	Illumina	NM_002568	exon13	c.C1705T	p.L569F	439	0.0615
HAUS6	hs37d5	9	19058888	19058888	nonsynonymous_SNV	SNP	G	G	C	patient 4	WXS	Illumina	NM_017645	exon16	c.C1877G	p.P626R	234	0.0556
FAM75A6	hs37d5	9	43624850	43624850	synonymous_SNV	SNP	C	C	T	patient 4	WXS	Illumina	NM_001145196	exon4	c.G3837A	p.R1279R	38	0.8947
DAB2IP	hs37d5	9	124441035	124441035	nonsynonymous_SNV	SNP	C	C	T	patient 4	WXS	Illumina	NM_032552	exon2	c.C119T	p.S40L	74	0.1216
RC3H2	hs37d5	9	125620216	125620216	nonsynonymous_SNV	SNP	C	C	A	patient 4	WXS	Illumina	NM_001100588	exon13	c.G2440T	p.D814Y	115	0.0609
TTC16	hs37d5	9	130482613	130482613	nonsynonymous_SNV	SNP	A	A	T	patient 4	WXS	Illumina	NM_144965	exon6	c.A596T	p.Q199L	40	0.2
TUBB8	hs37d5	10	93978	93978	synonymous_SNV	SNP	G	G	A	patient 4	WXS	Illumina	NM_177987	exon4	c.C354T	p.D118D	170	0.1
FRMD4A	hs37d5	10	13712422	13712422	nonsynonymous_SNV	SNP	A	A	C	patient 4	WXS	Illumina	NM_018027	exon17	c.T1358G	p.I453S	75	0.24
DNHD1	hs37d5	11	6580254	6580254	nonsynonymous_SNV	SNP	C	C	A	patient 4	WXS	Illumina	NM_144666	exon27	c.C9334A	p.P3112T	73	0.3288
CCDC73	hs37d5	11	32635293	32635293	nonsynonymous_SNV	SNP	C	C	A	patient 4	WXS	Illumina	NM_001008391	exon16	c.G2571T	p.M857I	64	0.2656
OR8H3	hs37d5	11	55889972	55889972	nonsynonymous_SNV	SNP	G	G	A	patient 4	WXS	Illumina	NM_001005201	exon1	c.G124A	p.G42R	230	0.0348
RCOR2	hs37d5	11	63683079	63683079	nonsynonymous_SNV	SNP	G	G	T	patient 4	WXS	Illumina	NM_173587	exon2	c.C132A	p.S44R	77	0.0909
DLG2	hs37d5	11	84996344	84996344	nonsynonymous_SNV	SNP	C	C	T	patient 4	WXS	Illumina	NM_001142699	exon4	c.G106A	p.E36K	187	0.2941
VWA5A	hs37d5	11	123989688	123989691	nonframeshift_deletion	DEL	CTGG	CTGG	C	patient 4	WXS	Illumina	NM_014622	exon6	c.653_655del	p.218_219del	84	0.369
KRT8	hs37d5	12	53291239	53291239	synonymous_SNV	SNP	A	A	G	patient 4	WXS	Illumina	NM_001256282	exon9	c.T1509C	p.S503S	152	0.1776
KRT8	hs37d5	12	53291266	53291266	synonymous_SNV	SNP	G	G	A	patient 4	WXS	Illumina	NM_001256282	exon9	c.C1482T	p.I494I	168	0.1786
C12orf12	hs37d5	12	91347621	91347621	nonsynonymous_SNV	SNP	G	G	C	patient 4	WXS	Illumina	NM_152638	exon1	c.C899G	p.A300G	93	0.0645
MPHOSPH8	hs37d5	13	20244394	20244394	nonsynonymous_SNV	SNP	T	T	A	patient 4	WXS	Illumina	NM_017520	exon12	c.T2348A	p.F783Y	127	0.2441
MPHOSPH8	hs37d5	13	20244395	20244395	nonsynonymous_SNV	SNP	T	T	A	patient 4	WXS	Illumina	NM_017520	exon12	c.T2349A	p.F783L	128	0.25
SYNE2	hs37d5	14	64641815	64641815	stopgain_SNV	SNP	C	C	T	patient 4	WXS	Illumina	NM_182914	exon95	c.C17389T	p.Q5797X	136	0.4485
AHNAK2	hs37d5	14	105418260	105418260	synonymous_SNV	SNP	T	T	A	patient 4	WXS	Illumina	NM_138420	exon7	c.A3528T	p.S1176S	147	0.0748
POTEB	hs37d5	15	21051201	21051201	nonsynonymous_SNV	SNP	T	T	C	patient 4	WXS	Illumina	NM_207355	exon9	c.A1259G	p.N420S	522	0.0345
JMJD7-PLA2G4B	hs37d5	15	42126939	42126939	splicing	SNP	G	G	T	patient 4	WXS	Illumina	0	0	0	0	67	0.8358
CCDC154	hs37d5	16	1485971	1485971	splicing	SNP	G	G	A	patient 4	WXS	Illumina	0	0	0	0	69	0.1449
NPIP	hs37d5	16	15045766	15045766	nonsynonymous_SNV	SNP	C	C	T	patient 4	WXS	Illumina	NM_006985	exon8	c.C937T	p.L313F	121	0.1736
ATP2A1	hs37d5	16	28913508	28913508	synonymous_SNV	SNP	C	C	T	patient 4	WXS	Illumina	NM_173201	exon17	c.C2325T	p.I775I	57	0.3684
IST1	hs37d5	16	71956511	71956517	nonframeshift_deletion	DEL	AATGCCC	AATGCCC	A	patient 4	WXS	Illumina	NM_014761	exon7	c.688_693del	p.230_231del	306	0.1307
KSR1	hs37d5	17	25909867	25909867	nonsynonymous_SNV	SNP	C	C	A	patient 4	WXS	Illumina	NM_014238	exon5	c.C305A	p.P102H	95	0.2947
NF1	hs37d5	17	29541542	29541542	nonsynonymous_SNV	SNP	A	A	G	patient 4	WXS	Illumina	NM_001042492	exon13	c.A1466G	p.Y489C	384	0.526
TBX4	hs37d5	17	59544924	59544924	nonsynonymous_SNV	SNP	C	C	T	patient 4	WXS	Illumina	NM_018488	exon4	c.C455T	p.P152L	163	0.2515
POTEC	hs37d5	18	14533105	14533105	nonsynonymous_SNV	SNP	G	G	C	patient 4	WXS	Illumina	NM_001137671	exon5	c.C1010G	p.S337C	299	0.087
PTBP1	hs37d5	19	808433	808433	synonymous_SNV	SNP	C	C	T	patient 4	WXS	Illumina	NM_002819	exon12	c.C1227T	p.D409D	112	0.3125
PLK5	hs37d5	19	1535205	1535205	nonsynonymous_SNV	SNP	G	G	C	patient 4	WXS	Illumina	NM_001243079	exon14	c.G967C	p.G323R	66	0.1212
PLIN5	hs37d5	19	4525822	4525828	nonframeshift_deletion	DEL	TTCAGCC	TTCAGCC	T	patient 4	WXS	Illumina	NM_001013706	exon6	c.537_542del	p.179_181del	146	0.1575
C19orf38	hs37d5	19	10979921	10979921	nonsynonymous_SNV	SNP	A	A	G	patient 4	WXS	Illumina	NM_001136482	exon7	c.A571G	p.T191A	200	0.065
ZNF443	hs37d5	19	12541700	12541700	nonsynonymous_SNV	SNP	G	G	T	patient 4	WXS	Illumina	NM_005815	exon4	c.C1286A	p.A429D	347	0.049
CHERP	hs37d5	19	16636131	16636131	nonsynonymous_SNV	SNP	C	C	T	patient 4	WXS	Illumina	NM_006387	exon10	c.G1663A	p.D555N	72	0.1389
CHERP	hs37d5	19	16643478	16643478	nonsynonymous_SNV	SNP	G	G	A	patient 4	WXS	Illumina	NM_006387	exon5	c.C605T	p.T202M	114	0.0526
DYRK1B	hs37d5	19	40319133	40319133	nonsynonymous_SNV	SNP	G	G	A	patient 4	WXS	Illumina	NM_004714	exon6	c.C611T	p.T204I	45	0.4
MYBPC2	hs37d5	19	50954555	50954555	nonsynonymous_SNV	SNP	T	T	A	patient 4	WXS	Illumina	NM_004533	exon15	c.T1598A	p.V533D	55	0.1091
SSC5D	hs37d5	19	56029713	56029713	nonsynonymous_SNV	SNP	C	C	G	patient 4	WXS	Illumina	NM_001144950	exon14	c.C4070G	p.T1357R	76	0.0658
SRSF6	hs37d5	20	42089538	42089538	nonsynonymous_SNV	SNP	G	G	C	patient 4	WXS	Illumina	NM_006275	exon6	c.G870C	p.K290N	229	0.2009
GAB4	hs37d5	22	17445724	17445724	nonsynonymous_SNV	SNP	G	G	T	patient 4	WXS	Illumina	NM_001037814	exon8	c.C1408A	p.H470N	88	0.0682
BAIAP2L2	hs37d5	22	38483226	38483226	synonymous_SNV	SNP	C	C	T	patient 4	WXS	Illumina	NM_025045	exon11	c.G1164A	p.G388G	79	0.0759
CELSR1	hs37d5	22	46762947	46762947	synonymous_SNV	SNP	G	G	A	patient 4	WXS	Illumina	NM_014246	exon29	c.C8148T	p.L2716L	67	0.0896
USP51	hs37d5	X	55515116	55515116	stopgain_SNV	SNP	G	G	T	patient 4	WXS	Illumina	NM_201286	exon2	c.C257A	p.S86X	77	0.2208
BRWD3	hs37d5	X	79971707	79971707	nonsynonymous_SNV	SNP	G	G	C	patient 4	WXS	Illumina	NM_153252	exon20	c.C2274G	p.I758M	87	0.4253
PLCH2	hs37d5	1	2435787	2435787	nonsynonymous_SNV	SNP	C	C	T	patient 5	WXS	Illumina	NM_014638	exon22	c.C3386T	p.P1129L	49	0.102
PRAMEF1	hs37d5	1	12854068	12854068	nonsynonymous_SNV	SNP	T	T	C	patient 5	WXS	Illumina	NM_023013	exon3	c.T292C	p.W98R	216	0.088
PRAMEF1	hs37d5	1	12855691	12855691	nonsynonymous_SNV	SNP	A	A	T	patient 5	WXS	Illumina	NM_023013	exon4	c.A971T	p.H324L	132	0.0833
PRAMEF1	hs37d5	1	12855898	12855898	nonsynonymous_SNV	SNP	C	C	T	patient 5	WXS	Illumina	NM_023013	exon4	c.C1178T	p.A393V	120	0.0833
PRAMEF1	hs37d5	1	12855962	12855962	synonymous_SNV	SNP	C	C	T	patient 5	WXS	Illumina	NM_023013	exon4	c.C1242T	p.A414A	239	0.0795
PRAMEF1	hs37d5	1	12856079	12856079	synonymous_SNV	SNP	C	C	T	patient 5	WXS	Illumina	NM_023013	exon4	c.C1359T	p.G453G	510	0.0333
PRAMEF1	hs37d5	1	12856106	12856106	synonymous_SNV	SNP	A	A	G	patient 5	WXS	Illumina	NM_023013	exon4	c.A1386G	p.S462S	417	0.048
PRAMEF11	hs37d5	1	12885059	12885059	nonsynonymous_SNV	SNP	C	C	G	patient 5	WXS	Illumina	NM_001146344	exon4	c.G1052C	p.C351S	111	0.0721
HNRNPCL1	hs37d5	1	12907507	12907507	synonymous_SNV	SNP	C	C	T	patient 5	WXS	Illumina	NM_001013631	exon2	c.G636A	p.E212E	98	0.0714
HNRNPCL1	hs37d5	1	12907508	12907508	nonsynonymous_SNV	SNP	T	T	C	patient 5	WXS	Illumina	NM_001013631	exon2	c.A635G	p.E212G	97	0.0722
PRAMEF4	hs37d5	1	12939495	12939495	nonsynonymous_SNV	SNP	A	A	T	patient 5	WXS	Illumina	NM_001009611	exon4	c.T1307A	p.I436N	178	0.1067
PRAMEF4	hs37d5	1	12939535	12939535	nonsynonymous_SNV	SNP	C	C	T	patient 5	WXS	Illumina	NM_001009611	exon4	c.G1267A	p.G423S	234	0.0812
PRAMEF10	hs37d5	1	12954490	12954490	nonsynonymous_SNV	SNP	A	A	G	patient 5	WXS	Illumina	NM_001039361	exon3	c.T793C	p.C265R	324	0.0494
PRAMEF10	hs37d5	1	12954890	12954890	synonymous_SNV	SNP	C	C	T	patient 5	WXS	Illumina	NM_001039361	exon3	c.G393A	p.K131K	160	0.0625
PRAMEF7	hs37d5	1	12979845	12979845	nonsynonymous_SNV	SNP	T	T	C	patient 5	WXS	Illumina	NM_001012277	exon3	c.T1037C	p.V346A	130	0.1308
PRAMEF22	hs37d5	1	13036654	13036654	synonymous_SNV	SNP	C	C	T	patient 5	WXS	Illumina	NM_001100631	exon2	c.C726T	p.S242S	127	0.2047
PRAMEF19	hs37d5	1	13474974	13474974	synonymous_SNV	SNP	G	G	A	patient 5	WXS	Illumina	NM_001099790	exon3	c.C1155T	p.H385H	75	0.2
RAP1GAP	hs37d5	1	21938223	21938223	synonymous_SNV	SNP	C	C	G	patient 5	WXS	Illumina	NM_001145658	exon12	c.G891C	p.L297L	84	0.0714
ZMYM6	hs37d5	1	35485136	35485136	synonymous_SNV	SNP	T	T	G	patient 5	WXS	Illumina	NM_007167	exon4	c.A246C	p.S82S	107	0.1215
ORC1	hs37d5	1	52861831	52861831	nonsynonymous_SNV	SNP	G	G	A	patient 5	WXS	Illumina	NM_001190818	exon5	c.C608T	p.T203I	120	0.0917
AGL	hs37d5	1	100366280	100366280	nonsynonymous_SNV	SNP	C	C	T	patient 5	WXS	Illumina	NM_000644	exon26	c.C3451T	p.R1151W	136	0.1103
GSTM4	hs37d5	1	110201684	110201684	nonsynonymous_SNV	SNP	C	C	G	patient 5	WXS	Illumina	NM_000850	exon7	c.C519G	p.N173K	191	0.0733
RBM15	hs37d5	1	110882903	110882903	synonymous_SNV	SNP	A	A	C	patient 5	WXS	Illumina	NM_022768	exon1	c.A876C	p.S292S	80	0.1
FAM72B	hs37d5	1	120846060	120846060	nonsynonymous_SNV	SNP	G	G	A	patient 5	WXS	Illumina	NM_001100910	exon3	c.G296A	p.G99E	261	0.0728
PDE4DIP	hs37d5	1	144952207	144952207	nonsynonymous_SNV	SNP	C	C	T	patient 5	WXS	Illumina	NM_001198834	exon4	c.G512A	p.R171K	381	0.1024
NOTCH2NL	hs37d5	1	145273431	145273431	synonymous_SNV	SNP	A	A	G	patient 5	WXS	Illumina	NM_203458	exon3	c.A285G	p.Q95Q	339	0.0324
NBPF10	hs37d5	1	145293420	145293420	synonymous_SNV	SNP	C	C	T	patient 5	WXS	Illumina	NM_001039703	exon1	c.C15T	p.A5A	915	0.0317
NBPF10	hs37d5	1	145293469	145293469	nonsynonymous_SNV	SNP	G	G	C	patient 5	WXS	Illumina	NM_001039703	exon1	c.G64C	p.E22Q	902	0.0333
NBPF10	hs37d5	1	145293515	145293515	nonsynonymous_SNV	SNP	A	A	G	patient 5	WXS	Illumina	NM_001039703	exon1	c.A110G	p.N37S	810	0.0914
NBPF10	hs37d5	1	145299758	145299758	synonymous_SNV	SNP	C	C	T	patient 5	WXS	Illumina	NM_001039703	exon6	c.C807T	p.N269N	184	0.1087
NBPF10	hs37d5	1	145368461	145368461	nonsynonymous_SNV	SNP	A	A	C	patient 5	WXS	Illumina	NM_001039703	exon84	c.A10439C	p.E3480A	552	0.1304
POLR3GL	hs37d5	1	145460120	145460120	synonymous_SNV	SNP	G	G	A	patient 5	WXS	Illumina	NM_032305	exon2	c.C103T	p.L35L	81	0.2099
NBPF24	hs37d5	1	146057292	146057292	nonsynonymous_SNV	SNP	T	T	C	patient 5	WXS	Illumina	NM_001101663	exon2	c.A158G	p.N53S	150	0.0733
NBPF14	hs37d5	1	148009510	148009510	synonymous_SNV	SNP	G	G	A	patient 5	WXS	Illumina	NM_015383	exon16	c.C1797T	p.S599S	115	0.3304
NBPF15	hs37d5	1	148589005	148589005	nonsynonymous_SNV	SNP	G	G	A	patient 5	WXS	Illumina	NM_173638	exon12	c.G1000A	p.D334N	139	0.1295
PPIAL4E	hs37d5	1	148644408	148644408	nonsynonymous_SNV	SNP	A	A	G	patient 5	WXS	Illumina	NM_001144032	exon1	c.A323G	p.N108S	128	0.2266
ZNF687	hs37d5	1	151262986	151262986	nonsynonymous_SNV	SNP	G	G	T	patient 5	WXS	Illumina	NM_020832	exon8	c.G3156T	p.Q1052H	21	0.2857
HRNR	hs37d5	1	152187606	152187606	nonsynonymous_SNV	SNP	G	G	A	patient 5	WXS	Illumina	NM_001009931	exon3	c.C6499T	p.R2167C	305	0.1607
FLG	hs37d5	1	152280614	152280614	nonsynonymous_SNV	SNP	C	C	G	patient 5	WXS	Illumina	NM_002016	exon3	c.G6748C	p.E2250Q	135	0.1185
FLG	hs37d5	1	152280907	152280907	nonsynonymous_SNV	SNP	G	G	T	patient 5	WXS	Illumina	NM_002016	exon3	c.C6455A	p.S2152Y	135	0.0667
FLG	hs37d5	1	152281172	152281172	nonsynonymous_SNV	SNP	T	T	G	patient 5	WXS	Illumina	NM_002016	exon3	c.A6190C	p.K2064Q	185	0.0649
FLG	hs37d5	1	152281466	152281466	synonymous_SNV	SNP	C	C	T	patient 5	WXS	Illumina	NM_002016	exon3	c.G5896A	p.G1966S	173	0.0694
NUP210L	hs37d5	1	154112399	154112399	nonsynonymous_SNV	SNP	G	G	A	patient 5	WXS	Illumina	NM_207308	exon5	c.C596T	p.A199V	148	0.0473
APOA1BP	hs37d5	1	156562212	156562212	nonsynonymous_SNV	SNP	G	G	T	patient 5	WXS	Illumina	NM_144772	exon3	c.G347T	p.G116V	120	0.0667
PTGS2	hs37d5	1	186648280	186648280	nonsynonymous_SNV	SNP	G	G	T	patient 5	WXS	Illumina	NM_000963	exon3	c.C223A	p.H75N	179	0.1061
CFH	hs37d5	1	196712598	196712598	synonymous_SNV	SNP	T	T	C	patient 5	WXS	Illumina	NM_000186	exon20	c.T3150C	p.N1050N	164	0.0427
C1orf106	hs37d5	1	200880861	200880861	nonsynonymous_SNV	SNP	G	G	C	patient 5	WXS	Illumina	NM_018265	exon9	c.G1537C	p.G513R	41	0.1463
CNIH3	hs37d5	1	224922394	224922394	synonymous_SNV	SNP	C	C	T	patient 5	WXS	Illumina	NM_152495	exon5	c.C441T	p.F147F	79	0.1392
OBSCN	hs37d5	1	228466586	228466586	synonymous_SNV	SNP	C	C	T	patient 5	WXS	Illumina	NM_001098623	exon26	c.C7056T	p.H2352H	30	0.2
FH	hs37d5	1	241663824	241663824	nonsynonymous_SNV	SNP	C	C	T	patient 5	WXS	Illumina	NM_000143	exon9	c.G1303A	p.V435M	98	0.0612
AHCTF1	hs37d5	1	247054344	247054344	nonsynonymous_SNV	SNP	T	T	C	patient 5	WXS	Illumina	NM_015446	exon16	c.A1991G	p.N664S	86	0.1512
OR2M5	hs37d5	1	248308934	248308934	nonsynonymous_SNV	SNP	C	C	T	patient 5	WXS	Illumina	NM_001004690	exon1	c.C485T	p.A162V	173	0.0867
OR2T4	hs37d5	1	248525135	248525135	nonsynonymous_SNV	SNP	G	G	A	patient 5	WXS	Illumina	NM_001004696	exon1	c.G253A	p.A85T	156	0.1346
OR2T4	hs37d5	1	248525138	248525138	nonsynonymous_SNV	SNP	C	C	T	patient 5	WXS	Illumina	NM_001004696	exon1	c.C256T	p.H86Y	158	0.0506
OR2T3	hs37d5	1	248637480	248637480	nonsynonymous_SNV	SNP	A	A	G	patient 5	WXS	Illumina	NM_001005495	exon1	c.A829G	p.M277V	202	0.0594
OR2T29	hs37d5	1	248722777	248722777	nonsynonymous_SNV	SNP	T	T	A	patient 5	WXS	Illumina	NM_001004694	exon1	c.A16T	p.R6W	87	0.1954
OTOF	hs37d5	2	26689681	26689681	synonymous_SNV	SNP	G	G	A	patient 5	WXS	Illumina	NM_194248	exon36	c.C4401T	p.D1467D	38	0.2368
ASB3	hs37d5	2	53977997	53977997	nonsynonymous_SNV	SNP	T	T	A	patient 5	WXS	Illumina	NM_016115	exon3	c.A278T	p.K93I	104	0.1635
SMEK2	hs37d5	2	55791601	55791601	nonsynonymous_SNV	SNP	C	C	T	patient 5	WXS	Illumina	NM_001122964	exon15	c.G2108A	p.R703H	131	0.1527
USP34	hs37d5	2	61415431	61415431	synonymous_SNV	SNP	A	A	G	patient 5	WXS	Illumina	NM_014709	exon80	c.T10447C	p.L3483L	123	0.0732
KDM3A	hs37d5	2	86683579	86683579	nonsynonymous_SNV	SNP	G	G	C	patient 5	WXS	Illumina	NM_001146688	exon6	c.G571C	p.V191L	122	0.082
FAHD2B	hs37d5	2	97757249	97757249	synonymous_SNV	SNP	C	C	T	patient 5	WXS	Illumina	NM_199336	exon2	c.G195A	p.T65T	133	0.0677
LIMS1	hs37d5	2	109276099	109276099	nonsynonymous_SNV	SNP	G	G	A	patient 5	WXS	Illumina	NM_001193485	exon2	c.G221A	p.R74H	93	0.1398
POTEF	hs37d5	2	130833081	130833081	nonsynonymous_SNV	SNP	A	A	G	patient 5	WXS	Illumina	NM_001099771	exon17	c.T1964C	p.I655T	307	0.1303
ARHGEF4	hs37d5	2	131801941	131801941	synonymous_SNV	SNP	C	C	T	patient 5	WXS	Illumina	NM_015320	exon12	c.C1669T	p.L557L	46	0.1087
SPC25	hs37d5	2	169745791	169745791	synonymous_SNV	SNP	A	A	G	patient 5	WXS	Illumina	NM_020675	exon3	c.T144C	p.S48S	117	0.1368
MPP4	hs37d5	2	202509944	202509944	nonsynonymous_SNV	SNP	C	C	G	patient 5	WXS	Illumina	NM_033066	exon22	c.G1903C	p.E635Q	45	0.1556
ALS2	hs37d5	2	202590117	202590117	synonymous_SNV	SNP	A	A	G	patient 5	WXS	Illumina	NM_020919	exon20	c.T3309C	p.H1103H	193	0.0829
EEF1B2	hs37d5	2	207026788	207026788	nonsynonymous_SNV	SNP	G	G	T	patient 5	WXS	Illumina	NM_001037663	exon5	c.G357T	p.R119S	193	0.0674
EEF1B2	hs37d5	2	207026789	207026789	nonsynonymous_SNV	SNP	G	G	A	patient 5	WXS	Illumina	NM_001037663	exon5	c.G358A	p.E120K	196	0.0663
STK11IP	hs37d5	2	220478620	220478620	nonsynonymous_SNV	SNP	G	G	C	patient 5	WXS	Illumina	NM_052902	exon21	c.G2717C	p.R906T	53	0.1887
ANO7	hs37d5	2	242157228	242157228	synonymous_SNV	SNP	G	G	T	patient 5	WXS	Illumina	NM_001001891	exon20	c.G2262T	p.P754P	34	0.1765
APEH	hs37d5	3	49712658	49712658	nonsynonymous_SNV	SNP	T	T	G	patient 5	WXS	Illumina	NM_001640	exon3	c.T188G	p.F63C	45	0.4444
PDE12	hs37d5	3	57542211	57542211	nonsynonymous_SNV	SNP	G	G	C	patient 5	WXS	Illumina	NM_177966	exon1	c.G105C	p.M35I	55	0.1455
PDZRN3	hs37d5	3	73434922	73434922	nonsynonymous_SNV	SNP	C	C	G	patient 5	WXS	Illumina	NM_015009	exon9	c.G1533C	p.W511C	82	0.0976
CD96	hs37d5	3	111317054	111317054	stopgain_SNV	SNP	G	G	T	patient 5	WXS	Illumina	NM_198196	exon7	c.G943T	p.E315X	107	0.1776
FAM162A	hs37d5	3	122121717	122121717	nonsynonymous_SNV	SNP	G	G	A	patient 5	WXS	Illumina	NM_014367	exon2	c.G145A	p.G49R	118	0.1186
HSPBAP1	hs37d5	3	122471489	122471489	synonymous_SNV	SNP	G	G	A	patient 5	WXS	Illumina	NM_024610	exon6	c.C774T	p.Y258Y	150	0.1333
HEG1	hs37d5	3	124731806	124731806	nonsynonymous_SNV	SNP	C	C	T	patient 5	WXS	Illumina	NM_020733	exon6	c.G2617A	p.V873I	60	0.1
SLC12A8	hs37d5	3	124854556	124854556	synonymous_SNV	SNP	T	T	C	patient 5	WXS	Illumina	NM_001195483	exon5	c.A693G	p.E231E	96	0.1771
MCF2L2	hs37d5	3	182910812	182910812	synonymous_SNV	SNP	C	C	T	patient 5	WXS	Illumina	NM_015078	exon27	c.G2991A	p.P997P	85	0.0706
MUC20	hs37d5	3	195452005	195452005	synonymous_SNV	SNP	C	C	T	patient 5	WXS	Illumina	NM_152673	exon2	c.C531T	p.S177S	38	0.2368
MUC20	hs37d5	3	195452987	195452987	nonsynonymous_SNV	SNP	G	G	A	patient 5	WXS	Illumina	NM_152673	exon3	c.G1000A	p.A334T	77	0.1169
MUC20	hs37d5	3	195452991	195452991	nonsynonymous_SNV	SNP	C	C	T	patient 5	WXS	Illumina	NM_152673	exon3	c.C1004T	p.T335I	79	0.1139
LOC650293	hs37d5	4	8951506	8951506	synonymous_SNV	SNP	C	C	G	patient 5	WXS	Illumina	NM_001040071	exon1	c.C30G	p.L10L	114	0.1053
CABS1	hs37d5	4	71201691	71201691	nonsynonymous_SNV	SNP	T	T	C	patient 5	WXS	Illumina	NM_033122	exon1	c.T935C	p.V312A	123	0.0976
COX18	hs37d5	4	73923872	73923872	nonsynonymous_SNV	SNP	T	T	C	patient 5	WXS	Illumina	NM_173827	exon6	c.A961G	p.I321V	134	0.1567
CDS1	hs37d5	4	85530630	85530630	synonymous_SNV	SNP	G	G	A	patient 5	WXS	Illumina	NM_001263	exon3	c.G294A	p.S98S	140	0.1286
WDFY3	hs37d5	4	85687467	85687467	nonsynonymous_SNV	SNP	T	T	A	patient 5	WXS	Illumina	NM_014991	exon31	c.A4908T	p.R1636S	101	0.604

DSPP	hs37d5	4	88536550	88536550	synonymous_SNV	SNP	T	T	C	patient 5	WXS	Illumina	NM_014208	exon5	c.T2736C	p.S912S	167	0.0659
UNC5C	hs37d5	4	96140271	96140271	synonymous_SNV	SNP	C	C	T	patient 5	WXS	Illumina	NM_003728	exon9	c.G1494A	p.T498T	42	0.1905
ABCE1	hs37d5	4	146041139	146041139	synonymous_SNV	SNP	T	T	C	patient 5	WXS	Illumina	NM_001040876	exon11	c.T978C	p.D326D	135	0.0815
PET112	hs37d5	4	152679941	152679941	nonsynonymous_SNV	SNP	G	G	C	patient 5	WXS	Illumina	NM_004564	exon2	c.C310G	p.L104V	164	0.0488
GLRB	hs37d5	4	157999183	157999183	nonsynonymous_SNV	SNP	T	T	G	patient 5	WXS	Illumina	NM_001166060	exon2	c.T7G	p.F3V	138	0.1014
ZDHHC11	hs37d5	5	848742	848742	nonsynonymous_SNV	SNP	C	C	T	patient 5	WXS	Illumina	NM_024786	exon2	c.G256A	p.V86I	61	0.1148
C5orf42	hs37d5	5	37198881	37198881	nonsynonymous_SNV	SNP	G	G	A	patient 5	WXS	Illumina	NM_023073	exon20	c.C3595T	p.R1199W	141	0.1135
C5orf42	hs37d5	5	37243166	37243166	nonsynonymous_SNV	SNP	T	T	C	patient 5	WXS	Illumina	NM_023073	exon6	c.A626G	p.K209R	159	0.0881
UTP15	hs37d5	5	72863212	72863212	nonsynonymous_SNV	SNP	C	C	G	patient 5	WXS	Illumina	NM_032175	exon2	c.C43G	p.L15V	104	0.1058
PRDM6	hs37d5	5	122515853	122515853	synonymous_SNV	SNP	C	C	T	patient 5	WXS	Illumina	NM_001136239	exon7	c.C1509T	p.C503C	93	0.129
CEP120	hs37d5	5	122714043	122714043	nonsynonymous_SNV	SNP	C	C	T	patient 5	WXS	Illumina	NM_153223	exon16	c.G2183A	p.S728N	152	0.0592
PCDHB3	hs37d5	5	140481913	140481913	synonymous_SNV	SNP	C	C	T	patient 5	WXS	Illumina	NM_018937	exon1	c.C1680T	p.F560F	71	0.1268
PCDHB4	hs37d5	5	140503154	140503154	nonsynonymous_SNV	SNP	C	C	A	patient 5	WXS	Illumina	NM_018938	exon1	c.C1574A	p.A525E	84	0.131
TCERG1	hs37d5	5	145838635	145838653	nonframeshift_deletion	DEL	TCAGGCCCAGGCCCAGGCC	TCAGGCCCAGGCCCAGGCC	T	patient 5	WXS	Illumina	NM_006706	exon4	c.628_645del	p.210_215del	73	0.1507
DDX41	hs37d5	5	176938795	176938795	synonymous_SNV	SNP	G	G	A	patient 5	WXS	Illumina	NM_016222	exon17	c.C1866T	p.F622F	29	0.1724
C5orf60	hs37d5	5	179071958	179071958	nonsynonymous_SNV	SNP	C	C	G	patient 5	WXS	Illumina	NM_001142306	exon1	c.G64C	p.D22H	110	0.0727
LOC554223	hs37d5	6	29764971	29764971	splicing	SNP	A	A	G	patient 5	WXS	Illumina	0	0	0	0	144	0.1042
HLA-A	hs37d5	6	29910336	29910336	synonymous_SNV	SNP	C	C	G	patient 5	WXS	Illumina	NM_002116	exon1	c.C6G	p.A2A	185	0.0703
HLA-A	hs37d5	6	29910340	29910340	nonsynonymous_SNV	SNP	A	A	G	patient 5	WXS	Illumina	NM_002116	exon1	c.A10G	p.M4V	186	0.0699
HLA-A	hs37d5	6	29912087	29912087	nonsynonymous_SNV	SNP	G	G	T	patient 5	WXS	Illumina	NM_002116	exon4	c.G808T	p.A270S	86	0.1977
HSPA1A	hs37d5	6	31785453	31785453	synonymous_SNV	SNP	A	A	G	patient 5	WXS	Illumina	NM_005345	exon1	c.A1920G	p.V640V	101	0.0792
EHMT2	hs37d5	6	31860326	31860326	nonsynonymous_SNV	SNP	C	C	T	patient 5	WXS	Illumina	NM_006709	exon7	c.G722A	p.G241D	81	0.1481
HLA-DRB5	hs37d5	6	32487172	32487172	synonymous_SNV	SNP	C	C	T	patient 5	WXS	Illumina	NM_002125	exon3	c.G627A	p.V209V	208	0.0625
HLA-DRB5	hs37d5	6	32487174	32487174	nonsynonymous_SNV	SNP	C	C	G	patient 5	WXS	Illumina	NM_002125	exon3	c.G625C	p.V209L	208	0.0865
HLA-DRB5	hs37d5	6	32487268	32487268	synonymous_SNV	SNP	A	A	G	patient 5	WXS	Illumina	NM_002125	exon3	c.T531C	p.I177I	183	0.1093
HLA-DRB1	hs37d5	6	32548632	32548632	splicing	SNP	T	T	A	patient 5	WXS	Illumina	0	0	0	0	287	0.101
HLA-DQB1	hs37d5	6	32629137	32629137	nonsynonymous_SNV	SNP	T	T	G	patient 5	WXS	Illumina	NM_001243961	exon4	c.A759C	p.Q253H	99	0.101
HLA-DQB1	hs37d5	6	32629935	32629935	nonsynonymous_SNV	SNP	C	C	G	patient 5	WXS	Illumina	NM_001243961	exon3	c.G470C	p.G157A	40	0.225
HLA-DQB1	hs37d5	6	32632694	32632694	nonsynonymous_SNV	SNP	C	C	G	patient 5	WXS	Illumina	NM_001243961	exon2	c.G260C	p.R87P	61	0.082
HLA-DQA2	hs37d5	6	32713788	32713788	nonsynonymous_SNV	SNP	T	T	G	patient 5	WXS	Illumina	NM_020056	exon3	c.T552G	p.D184E	179	0.0559
HLA-DQA2	hs37d5	6	32713793	32713793	nonsynonymous_SNV	SNP	T	T	G	patient 5	WXS	Illumina	NM_020056	exon3	c.T557G	p.I186S	185	0.0595
HLA-DQA2	hs37d5	6	32713828	32713828	nonsynonymous_SNV	SNP	G	G	A	patient 5	WXS	Illumina	NM_020056	exon3	c.G592A	p.E198K	189	0.1005
PGC	hs37d5	6	41711064	41711064	nonsynonymous_SNV	SNP	G	G	A	patient 5	WXS	Illumina	NM_002630	exon4	c.C392T	p.S131F	67	0.0896
CD2AP	hs37d5	6	47576904	47576904	nonsynonymous_SNV	SNP	A	A	G	patient 5	WXS	Illumina	NM_012120	exon16	c.A1678G	p.T560A	176	0.0795
KIAA1009	hs37d5	6	84884494	84884494	synonymous_SNV	SNP	T	T	C	patient 5	WXS	Illumina	NM_014895	exon15	c.A1977G	p.E659E	119	0.1092
FUCA2	hs37d5	6	143832565	143832565	synonymous_SNV	SNP	G	G	A	patient 5	WXS	Illumina	NM_032020	exon1	c.C207T	p.F69F	77	0.1299
MTHFD1L	hs37d5	6	151355696	151355696	nonsynonymous_SNV	SNP	C	C	G	patient 5	WXS	Illumina	NM_001242767	exon25	c.C2657G	p.S886C	317	0.0599
TCP10L2	hs37d5	6	167591954	167591954	nonsynonymous_SNV	SNP	G	G	A	patient 5	WXS	Illumina	NM_001145121	exon5	c.G581A	p.R194H	99	0.0808
FAM120B	hs37d5	6	170632250	170632250	synonymous_SNV	SNP	C	C	A	patient 5	WXS	Illumina	NM_032448	exon3	c.C1818A	p.T606T	87	0.1034
PMS2	hs37d5	7	6026532	6026532	nonsynonymous_SNV	SNP	T	T	A	patient 5	WXS	Illumina	NM_000535	exon11	c.A1864T	p.M622L	182	0.0769
FKBP9	hs37d5	7	33015974	33015974	nonsynonymous_SNV	SNP	G	G	A	patient 5	WXS	Illumina	NM_007270	exon4	c.G566A	p.R189H	179	0.1006
AMPH	hs37d5	7	38505819	38505819	nonsynonymous_SNV	SNP	T	T	A	patient 5	WXS	Illumina	NM_001635	exon8	c.A620T	p.K207I	186	0.0699
POLM	hs37d5	7	44119479	44119479	nonsynonymous_SNV	SNP	T	T	A	patient 5	WXS	Illumina	NM_013284	exon3	c.A452T	p.H151L	73	0.1507
GTF2I	hs37d5	7	74167490	74167490	synonymous_SNV	SNP	C	C	T	patient 5	WXS	Illumina	NM_032999	exon29	c.C2622T	p.D874D	104	0.1731
SPDYE5	hs37d5	7	75124581	75124581	synonymous_SNV	SNP	G	G	A	patient 5	WXS	Illumina	NM_001099435	exon1	c.G147A	p.E49E	151	0.0795
CYP3A7	hs37d5	7	99305512	99305512	nonsynonymous_SNV	SNP	A	A	T	patient 5	WXS	Illumina	NM_000765	exon12	c.T1339A	p.F447I	184	0.0652
AZGP1	hs37d5	7	99573585	99573585	nonsynonymous_SNV	SNP	G	G	A	patient 5	WXS	Illumina	NM_001185	exon1	c.C59T	p.P20L	145	0.0483
MUC12	hs37d5	7	100637193	100637193	nonsynonymous_SNV	SNP	C	C	A	patient 5	WXS	Illumina	NM_001164462	exon2	c.C3349A	p.P1117T	149	0.1141
MUC12	hs37d5	7	100643675	100643675	synonymous_SNV	SNP	G	G	A	patient 5	WXS	Illumina	NM_001164462	exon2	c.G9831A	p.P3277P	638	0.0298
MUC12	hs37d5	7	100645825	100645825	nonsynonymous_SNV	SNP	C	C	A	patient 5	WXS	Illumina	NM_001164462	exon2	c.C11981A	p.T3994K	763	0.0433
MUC12	hs37d5	7	100646824	100646824	nonsynonymous_SNV	SNP	C	C	T	patient 5	WXS	Illumina	NM_001164462	exon2	c.C12980T	p.T4327I	212	0.0849
MUC17	hs37d5	7	100677333	100677333	nonsynonymous_SNV	SNP	C	C	G	patient 5	WXS	Illumina	NM_001040105	exon3	c.C2636G	p.T879S	183	0.071
MUC17	hs37d5	7	100677378	100677378	nonsynonymous_SNV	SNP	C	C	A	patient 5	WXS	Illumina	NM_001040105	exon3	c.C2681A	p.T894K	230	0.0478
MUC17	hs37d5	7	100677405	100677405	nonsynonymous_SNV	SNP	G	G	A	patient 5	WXS	Illumina	NM_001040105	exon3	c.G2708A	p.R903H	253	0.0553
MUC17	hs37d5	7	100677409	100677409	synonymous_SNV	SNP	G	G	A	patient 5	WXS	Illumina	NM_001040105	exon3	c.G2712A	p.S904S	252	0.0635
MUC17	hs37d5	7	100677415	100677415	synonymous_SNV	SNP	T	T	C	patient 5	WXS	Illumina	NM_001040105	exon3	c.T2718C	p.P906P	260	0.0962
MUC17	hs37d5	7	100677455	100677455	nonsynonymous_SNV	SNP	G	G	A	patient 5	WXS	Illumina	NM_001040105	exon3	c.G2758A	p.G920R	248	0.1573
MUC17	hs37d5	7	100677457	100677457	synonymous_SNV	SNP	G	G	T	patient 5	WXS	Illumina	NM_001040105	exon3	c.G2760T	p.G920G	240	0.075
MUC17	hs37d5	7	100677572	100677572	nonsynonymous_SNV	SNP	A	A	G	patient 5	WXS	Illumina	NM_001040105	exon3	c.A2875G	p.T959A	237	0.0591
MUC17	hs37d5	7	100677586	100677586	synonymous_SNV	SNP	A	A	G	patient 5	WXS	Illumina	NM_001040105	exon3	c.A2889G	p.S963S	237	0.0886
MUC17	hs37d5	7	100677625	100677625	synonymous_SNV	SNP	G	G	A	patient 5	WXS	Illumina	NM_001040105	exon3	c.G2928A	p.S976S	213	0.0751
MUC17	hs37d5	7	100677630	100677630	nonsynonymous_SNV	SNP	C	C	T	patient 5	WXS	Illumina	NM_001040105	exon3	c.C2933T	p.P978L	215	0.1628
MUC17	hs37d5	7	100677645	100677645	nonsynonymous_SNV	SNP	C	C	A	patient 5	WXS	Illumina	NM_001040105	exon3	c.C2948A	p.T983N	200	0.085
MUC17	hs37d5	7	100677704	100677704	nonsynonymous_SNV	SNP	C	C	T	patient 5	WXS	Illumina	NM_001040105	exon3	c.C3007T	p.L1003F	186	0.1129
MUC17	hs37d5	7	100677714	100677714	nonsynonymous_SNV	SNP	C	C	T	patient 5	WXS	Illumina	NM_001040105	exon3	c.C3017T	p.T1006I	192	0.1198
MUC17	hs37d5	7	100677944	100677944	nonsynonymous_SNV	SNP	T	T	C	patient 5	WXS	Illumina	NM_001040105	exon3	c.T3247C	p.S1083P	238	0.2479
MUC17	hs37d5	7	100677974	100677974	nonsynonymous_SNV	SNP	A	A	G	patient 5	WXS	Illumina	NM_001040105	exon3	c.A3277G	p.T1093A	219	0.0959
MUC17	hs37d5	7	100677988	100677988	nonsynonymous_SNV	SNP	T	T	A	patient 5	WXS	Illumina	NM_001040105	exon3	c.T3291A	p.S1097R	214	0.0374
MUC17	hs37d5	7	100677995	100677995	nonsynonymous_SNV	SNP	A	A	G	patient 5	WXS	Illumina	NM_001040105	exon3	c.A3298G	p.S1100G	199	0.0955
MUC17	hs37d5	7	100678013	100678013	nonsynonymous_SNV	SNP	G	G	A	patient 5	WXS	Illumina	NM_001040105	exon3	c.G3316A	p.V1106M	207	0.1111
MUC17	hs37d5	7	100678029	100678029	nonsynonymous_SNV	SNP	G	G	C	patient 5	WXS	Illumina	NM_001040105	exon3	c.G3332C	p.R1111T	193	0.057
MUC17	hs37d5	7	100678075	100678075	synonymous_SNV	SNP	C	C	T	patient 5	WXS	Illumina	NM_001040105	exon3	c.C3378T	p.V1126V	198	0.0657
MUC17	hs37d5	7	100678481	100678481	nonsynonymous_SNV	SNP	A	A	T	patient 5	WXS	Illumina	NM_001040105	exon3	c.A3784T	p.T1262S	168	0.1012
MUC17	hs37d5	7	100678483	100678483	synonymous_SNV	SNP	C	C	T	patient 5	WXS	Illumina	NM_001040105	exon3	c.C3786T	p.T1262T	162	0.1049
MUC17	hs37d5	7	100678560	100678560	nonsynonymous_SNV	SNP	C	C	A	patient 5	WXS	Illumina	NM_001040105	exon3	c.C3863A	p.T1288K	160	0.1437
MUC17	hs37d5	7	100678568	100678568	nonsynonymous_SNV	SNP	A	A	T	patient 5	WXS	Illumina	NM_001040105	exon3	c.A3871T	p.T1291S	163	0.1472
MUC17	hs37d5	7	100678610	100678610	nonsynonymous_SNV	SNP	A	A	T	patient 5	WXS	Illumina	NM_001040105	exon3	c.A3913T	p.T1305S	190	0.1316
MUC17	hs37d5	7	100678616	100678616	nonsynonymous_SNV	SNP	G	G	A	patient 5	WXS	Illumina	NM_001040105	exon3	c.G3919A	p.G1307S	195	0.1231
MUC17	hs37d5	7	100678693	100678693	synonymous_SNV	SNP	T	T	C	patient 5	WXS	Illumina	NM_001040105	exon3	c.T3996C	p.Y1332Y	156	0.0641
MUC17	hs37d5	7	100678988	100678988	nonsynonymous_SNV	SNP	G	G	A	patient 5	WXS	Illumina	NM_001040105	exon3	c.G4291A	p.A1431T	152	0.1776
MUC17	hs37d5	7	100679316	100679316	nonsynonymous_SNV	SNP	T	T	A	patient 5	WXS	Illumina	NM_001040105	exon3	c.T4619A	p.V1540D	169	0.1243
MUC17	hs37d5	7	100679318	100679318	nonsynonymous_SNV	SNP	A	A	C	patient 5	WXS	Illumina	NM_001040105	exon3	c.A4621C	p.T1541P	169	0.1006
MUC17	hs37d5	7	100679752	100679752	synonymous_SNV	SNP	T	T	C	patient 5	WXS	Illumina	NM_001040105	exon3	c.T5055C	p.Y1685Y	135	0.0889
MUC17	hs37d5	7	100679754	100679754	nonsynonymous_SNV	SNP	C	C	G	patient 5	WXS	Illumina	NM_001040105	exon3	c.C5057G	p.T1686S	131	0.1069
MUC17	hs37d5	7	100681266	100681266	nonsynonymous_SNV	SNP	C	C	A	patient 5	WXS	Illumina	NM_001040105	exon3	c.C6569A	p.T2190K	190	0.0789
MUC17	hs37d5	7	100683036	100683036	nonsynonymous_SNV	SNP	T	T	C	patient 5	WXS	Illumina	NM_001040105	exon3	c.T8339C	p.I2780T	183	0.0656
MUC17	hs37d5	7	100683053	100683053	nonsynonymous_SNV	SNP	A	A	G	patient 5	WXS	Illumina	NM_001040105	exon3	c.A8356G	p.T2786A	192	0.0365
TRIM56	hs37d5	7	100731873	100731873	nonsynonymous_SNV	SNP	C	C	T	patient 5	WXS	Illumina	NM_030961	exon3	c.C1280T	p.T427I	50	0.12
SPDYE6	hs37d5	7	101988983	101988983	nonsynonymous_SNV	SNP	G	G	A	patient 5	WXS	Illumina	NM_001146210	exon6	c.C890T	p.P297L	619	0.0323
LOC100289561	hs37d5	7	102016278	102016278	synonymous_SNV	SNP	G	G	A	patient 5	WXS	Illumina	NM_001242713	exon3	c.G24A	p.V8V	244	0.0574
POLR2J3	hs37d5	7	102212939	102212939	nonsynonymous_SNV	SNP	G	G	C	patient 5	WXS	Illumina	NM_001097615	exon1	c.C30G	p.F10L	295	0.0441
RASA4	hs37d5	7	102235769	102235769	nonsynonymous_SNV	SNP	T	T	C	patient 5	WXS	Illumina	NM_006989	exon11	c.A1054G	p.M352V	62	0.2742
RASA4	hs37d5	7	102246341	102246341	nonsynonymous_SNV	SNP	C	C	T	patient 5	WXS	Illumina	NM_006989	exon5	c.G392A	p.R131Q	59	0.2203
RASA4	hs37d5	7	102246349	102246349	synonymous_SNV	SNP	T	T	G	patient 5	WXS	Illumina	NM_006989	exon5	c.A384C	p.P128P	61	0.1311
WASL	hs37d5	7	123344708	123344708	nonsynonymous_SNV	SNP	T	T	C	patient 5	WXS	Illumina	NM_003941	exon5	c.A440G	p.K147R	163	0.5706
GIMAP8	hs37d5	7	150174688	150174688	synonymous_SNV	SNP	A	A	T	patient 5	WXS	Illumina	NM_175571	exon5	c.A1818T	p.T606T	156	0.1154
RP1L1	hs37d5	8	10467680	10467686	nonframeshift_deletion	DEL	TTCCTTC	TTCCTTC	T	patient 5	WXS	Illumina	NM_178857	exon4	c.3922_3927del	p.1308_1309del	171	0.0468
ZNF705D	hs37d5	8	11970652	11970652	synonymous_SNV	SNP	C	C	T	patient 5	WXS	Illumina	NM_001039615	exon7	c.C888T	p.S296S	176	0.1136
NEFL	hs37d5	8	24811072	24811072	synonymous_SNV	SNP	C	C	T	patient 5	WXS	Illumina	NM_006158	exon3	c.G1407A	p.E469E	242	0.062
KAT6A	hs37d5	8	41792177	41792177	synonymous_SNV	SNP	G	G	A	patient 5	WXS	Illumina	NM_001099412	exon18	c.C3561T	p.C1187C	189	0.0741
EFR3A	hs37d5	8	132980614	132980614	nonsynonymous_SNV	SNP	G	G	T	patient 5	WXS	Illumina	NM_015137	exon9	c.G928T	p.V310F	190	0.0789
NAPRT1	hs37d5	8	144657830	144657830	nonsynonymous_SNV	SNP	T	T	C	patient 5	WXS	Illumina	NM_145201	exon9	c.A1150G	p.T384A	112	0.0714
EPPK1	hs37d5	8	144940331	144940331	nonsynonymous_SNV	SNP	C	C	T	patient 5	WXS	Illumina	NM_031308	exon1	c.G7091A	p.R2364Q	276	0.0399
WASH1	hs37d5	9	14816	14816	nonsynonymous_SNV	SNP	C	C	G	patient 5	WXS	Illumina	NM_182905	exon11	c.G1389C	p.W463C	55	0.2909
C9orf123	hs37d5	9	7799509	7799509	stopgain_SNV	SNP	C	C	A	patient 5	WXS	Illumina	NM_033428	exon1	c.G226T	p.G76X	100	0.17
C9orf123	hs37d5	9	7799516	7799516	nonsynonymous_SNV	SNP	C	C	T	patient 5	WXS	Illumina	NM_033428	exon1	c.G219A	p.M73I	105	0.1714
ACER2	hs37d5	9	19424725	19424725	nonsynonymous_SNV	SNP	C	C	T	patient 5	WXS	Illumina	NM_001010887	exon3	c.C251T	p.T84I	92	0.1522
DMRTA1	hs37d5	9	22451843	22451843	nonsynonymous_SNV	SNP	A	A	G	patient 5	WXS	Illumina	NM_022160	exon2	c.A1448G	p.Y483C	67	0.0746
FAM205A	hs37d5	9	34725742	34725742	nonsynonymous_SNV	SNP	T	T	C	patient 5	WXS	Illumina	NM_001141917	exon4	c.A1495G	p.M499V	78	0.0897
FAM75A3	hs37d5	9	40702846	40702846	nonsynonymous_SNV	SNP	C	C	T	patient 5	WXS	Illumina	NM_001083124	exon4	c.C503T	p.T168I	99	0.1212
ANKRD20A2	hs37d5	9	42368549	42368549	synonymous_SNV	SNP	C	C	T	patient 5	WXS	Illumina	NM_001012421	exon1	c.C135T	p.D45D	124	0.0645
NOL8	hs37d5	9	95068059	95068059	nonsynonymous_SNV	SNP	T	T	C	patient 5	WXS	Illumina	NM_017948	exon12	c.A2879G	p.K960R	120	0.0833
PHF2	hs37d5	9	96439235	96439235	synonymous_SNV	SNP	C	C	T	patient 5	WXS	Illumina	NM_005392	exon21	c.C3192T	p.T1064T	91	0.0989
FAM22G	hs37d5	9	99694501	99694501	nonsynonymous_SNV	SNP	C	C	T	patient 5	WXS	Illumina	NM_001170741	exon2	c.C514T	p.P172S	224	0.0491
EDF1	hs37d5	9	139756784	139756784	synonymous_SNV	SNP	C	C	T	patient 5	WXS	Illumina	NM_003792	exon5	c.G399A	p.R133R	56	0.0893
CACNA1B	hs37d5	9	140777299	140777299	nonsynonymous_SNV	SNP	G	G	T	patient 5	WXS	Illumina	NM_000718	exon3	c.G494T	p.G165V	136	0.0809
TUBB8	hs37d5	10	93843	93843	synonymous_SNV	SNP	G	G	A	patient 5	WXS	Illumina	NM_177987	exon4	c.C489T	p.I163I	234	0.0427
PFKP	hs37d5	10	3162102	3162102	nonsynonymous_SNV	SNP	G	G	A	patient 5	WXS	Illumina	NM_002627	exon16	c.G1549A	p.E517K	69	0.2174
AKR1C3	hs37d5	10	5147824	5147824	nonsynonymous_SNV	SNP	C	C	A	patient 5	WXS	Illumina	NM_001253908	exon8	c.C884A	p.A295D	190	0.0579
USP6NL	hs37d5	10	11505627	11505627	nonsynonymous_SNV	SNP	T	T	G	patient 5	WXS	Illumina	NM_001080491	exon14	c.A1351C	p.K451Q	84	0.0833
BEND7	hs37d5	10	13541788	13541788	synonymous_SNV	SNP	C	C	T	patient 5	WXS	Illumina	NM_152751	exon3	c.G282A	p.T94T	108	0.1759
PTER	hs37d5	10	16547025	16547025	nonsynonymous_SNV	SNP	T	T	G	patient 5	WXS	Illumina	NM_030664	exon5	c.T705G	p.I235M	190	0.1053
FRMPD2	hs37d5	10	49383976	49383976	nonsynonymous_SNV	SNP	T	T	C	patient 5	WXS	Illumina	NM_001018071	exon23	c.A2902G	p.I968V	153	0.085
AGAP6	hs37d5	10	51769579	51769579	nonsynonymous_SNV	SNP	G	G	A	patient 5	WXS	Illumina	NM_001077665	exon8	c.G1694A	p.R565Q	85	0.0824
TSPAN15	hs37d5	10	71243452	71243452	nonsynonymous_SNV	SNP	T	T	G	patient 5	WXS	Illumina	NM_012339	exon2	c.T102G	p.I34M	68	0.2059
EIF5AL1	hs37d5	10	81272814	81272814	nonsynonymous_SNV	SNP	G	G	C	patient 5	WXS	Illumina	NM_001099692	exon1	c.G409C	p.V137L	123	0.065
LIPJ	hs37d5	10	90353896	90353896	synonymous_SNV	SNP	T	T	C	patient 5	WXS	Illumina	NM_001010939	exon5	c.T324C	p.A108A	82	0.122
KIF11	hs37d5	10	94409648	94409648	nonsynonymous_SNV	SNP	C	C	G	patient 5	WXS	Illumina	NM_004523	exon20	c.C2827G	p.P943A	168	0.0655
DNMBP	hs37d5	10	101716772	101716772	synonymous_SNV	SNP	T	T	C	patient 5	WXS	Illumina	NM_015221	exon4	c.A459G	p.L153L	69	0.1014
SUFU	hs37d5	10	104375096	104375096	nonsynonymous_SNV	SNP	A	A	T	patient 5	WXS	Illumina	NM_016169	exon9	c.A1094T	p.Q365L	44	0.1364
PNLIPRP2	hs37d5	10	118386509	118386509	splicing	SNP	G	G	C	patient 5	WXS	Illumina	0	0	0	0	78	0.1795
FRG2B	hs37d5	10	135440203	135440203	nonsynonymous_SNV	SNP	A	A	T	patient 5	WXS	Illumina	NM_001080998	exon1	c.T44A	p.I15N	157	0.1401
MUC2	hs37d5	11	1093600	1093600	nonsynonymous_SNV	SNP	G	G	A	patient 5	WXS	Illumina	NM_002457	exon30	c.G5419A	p.G1807S	96	0.1562
MUC5B	hs37d5	11	1268661	1268661	synonymous_SNV	SNP	G	G	A	patient 5	WXS	Illumina	NM_002458	exon31	c.G10551A	p.R3517R	80	0.1375
STK33	hs37d5	11	8414090	8414090	synonymous_SNV	SNP	G	G	A	patient 5	WXS	Illumina	NM_030906	exon14	c.C1512T	p.S504S	170	0.0941
SAA1	hs37d5	11	18291356	18291356	nonsynonymous_SNV	SNP	A	A	G	patient 5	WXS	Illumina	NM_000331	exon4	c.A323G	p.K108R	78	0.141
LDHC	hs37d5	11	18460083	18460083	nonsynonymous_SNV	SNP	T	T	C	patient 5	WXS	Illumina	NM_002301	exon6	c.T601C	p.W201R	121	0.0744
PSMC3	hs37d5	11	47446681	47446681	synonymous_SNV	SNP	G	G	A	patient 5	WXS	Illumina	NM_002804	exon3	c.C276T	p.N92N	91	0.1868
SPRYD5	hs37d5	11	55653128	55653128	nonsynonymous_SNV	SNP	T	T	C	patient 5	WXS	Illumina	NM_032681	exon2	c.T224C	p.F75S	170	0.0471
SPRYD5	hs37d5	11	55658747	55658747	nonsynonymous_SNV	SNP	C	C	T	patient 5	WXS	Illumina	NM_032681	exon7	c.C998T	p.A333V	236	0.072
TMEM223	hs37d5	11	62558368	62558368	synonymous_SNV	SNP	A	A	G	patient 5	WXS	Illumina	NM_001080501	exon2	c.T336C	p.A112A	46	0.1304
SUV420H1	hs37d5	11	67957408	67957408	nonsynonymous_SNV	SNP	C	C	T	patient 5	WXS	Illumina	NM_017635	exon2	c.G136A	p.A46T	115	0.0696
ARHGEF17	hs37d5	11	73020731	73020731	nonsynonymous_SNV	SNP	C	C	T	patient 5	WXS	Illumina	NM_014786	exon1	c.C1048T	p.P350S	61	0.1148
EED	hs37d5	11	85979505	85979506	frameshift_deletion	DEL	AT	AT	A	patient 5	WXS	Illumina	NM_003797	exon9	c.869delT	p.I290fs	145	0.4276
JRKL	hs37d5	11	96124811	96124811	nonsynonymous_SNV	SNP	C	C	T	patient 5	WXS	Illumina	NM_003772	exon1	c.C998T	p.T333M	74	0.1892
FAM90A1	hs37d5	12	8375171	8375171	synonymous_SNV	SNP	T	T	G	patient 5	WXS	Illumina	NM_018088	exon7	c.A642C	p.A214A	151	0.053
CLEC12B	hs37d5	12	10170977	10170977	nonsynonymous_SNV	SNP	C	C	T	patient 5	WXS	Illumina	NM_001129998	exon6	c.C728T	p.A243V	174	0.046
FAM113B	hs37d5	12	47629845	47629845	synonymous_SNV	SNP	C	C	T	patient 5	WXS	Illumina	NM_138371	exon2	c.C999T	p.F333F	38	0.2105
MLL2	hs37d5	12	49420993	49420993	nonsynonymous_SNV	SNP	G	G	A	patient 5	WXS	Illumina	NM_003482	exon48	c.C14756T	p.S4919F	68	0.1176
MLL2	hs37d5	12	49424111	49424111	nonsynonymous_SNV	SNP	G	G	A	patient 5	WXS	Illumina	NM_003482	exon42	c.C13951T	p.H4651Y	42	0.1905
FAIM2	hs37d5	12	50290860	50290860	nonsynonymous_SNV	SNP	C	C	T	patient 5	WXS	Illumina	NM_012306	exon5	c.G388A	p.V130I	60	0.1
KRT86	hs37d5	12	52698808	52698808	nonsynonymous_SNV	SNP	G	G	C	patient 5	WXS	Illumina	NM_002284	exon4	c.G732C	p.E244D	53	0.1321
KRT6B	hs37d5	12	52845435	52845435	nonsynonymous_SNV	SNP	C	C	T	patient 5	WXS	Illumina	NM_005555	exon1	c.G428A	p.S143N	160	0.0688
KRT6A	hs37d5	12	52886917	52886917	nonsynonymous_SNV	SNP	C	C	T	patient 5	WXS	Illumina	NM_005554	exon1	c.G56A	p.S19N	66	0.1061
KRT8	hs37d5	12	53298675	53298675	nonsynonymous_SNV	SNP	A	A	C	patient 5	WXS	Illumina	NM_001256282	exon2	c.T175G	p.S59A	56	0.125
KRT8	hs37d5	12	53298699	53298699	nonsynonymous_SNV	SNP	G	G	A	patient 5	WXS	Illumina	NM_001256282	exon2	c.C151T	p.R51C	56	0.1607
TPH2	hs37d5	12	72366306	72366306	nonsynonymous_SNV	SNP	C	C	T	patient 5	WXS	Illumina	NM_173353	exon6	c.C616T	p.P206S	165	0.0788
C12orf42	hs37d5	12	103695950	103695950	nonsynonymous_SNV	SNP	C	C	T	patient 5	WXS	Illumina	NM_001099336	exon6	c.G1019A	p.R340Q	118	0.1017
PRDM4	hs37d5	12	108145763	108145763	synonymous_SNV	SNP	C	C	T	patient 5	WXS	Illumina	NM_012406	exon5	c.G555A	p.E185E	118	0.1017
ACACB	hs37d5	12	109614023	109614023	nonsynonymous_SNV	SNP	C	C	G	patient 5	WXS	Illumina	NM_001093	exon8	c.C1392G	p.I464M	122	0.0656
ATP2A2	hs37d5	12	110771846	110771846	nonsynonymous_SNV	SNP	A	A	T	patient 5	WXS	Illumina	NM_170665	exon11	c.A1317T	p.E439D	154	0.0649
CCDC168	hs37d5	13	103410900	103410900	synonymous_SNV	SNP	C	C	T	patient 5	WXS	Illumina	NM_001146197	exon2	c.G258A	p.R86R	128	0.0859
CARKD	hs37d5	13	111289549	111289549	synonymous_SNV	SNP	C	C	T	patient 5	WXS	Illumina	NM_018210	exon9	c.C846T	p.G282G	38	0.1579
OR11H12	hs37d5	14	19378312	19378312	nonsynonymous_SNV	SNP	T	T	G	patient 5	WXS	Illumina	NM_001013354	exon1	c.T719G	p.V240G	127	0.1181
POTEG	hs37d5	14	19566036	19566036	synonymous_SNV	SNP	C	C	T	patient 5	WXS	Illumina	NM_001005356	exon6	c.C1080T	p.Y360Y	769	0.1261
OR11H2	hs37d5	14	20181502	20181502	nonsynonymous_SNV	SNP	C	C	T	patient 5	WXS	Illumina	NM_001197287	exon2	c.G574A	p.V192I	155	0.0645
NIN	hs37d5	14	51245512	51245512	nonsynonymous_SNV	SNP	G	G	A	patient 5	WXS	Illumina	NM_020921	exon6	c.C446T	p.T149M	95	0.1368
C14orf1	hs37d5	14	76117924	76117924	nonsynonymous_SNV	SNP	C	C	G	patient 5	WXS	Illumina	NM_007176	exon5	c.G397C	p.V133L	91	0.1099
CPSF2	hs37d5	14	92604652	92604652	nonsynonymous_SNV	SNP	G	G	T	patient 5	WXS	Illumina	NM_017437	exon7	c.G622T	p.V208L	148	0.0811
AK7	hs37d5	14	96912892	96912892	nonsynonymous_SNV	SNP	A	A	G	patient 5	WXS	Illumina	NM_152327	exon8	c.A818G	p.H273R	124	0.0887
AHNAK2	hs37d5	14	105408832	105408832	nonsynonymous_SNV	SNP	G	G	A	patient 5	WXS	Illumina	NM_138420	exon7	c.C12956T	p.A4319V	96	0.0729
AHNAK2	hs37d5	14	105412005	105412005	synonymous_SNV	SNP	A	A	G	patient 5	WXS	Illumina	NM_138420	exon7	c.T9783C	p.D3261D	76	0.1447
AHNAK2	hs37d5	14	105412009	105412009	nonsynonymous_SNV	SNP	A	A	G	patient 5	WXS	Illumina	NM_138420	exon7	c.T9779C	p.M3260T	77	0.1688
GOLGA6L6	hs37d5	15	20740195	20740216	nonframeshift_deletion	DEL	TCTTCTCCTCCTGCCTCCACAC	TCTTCTCCTCCTGCCTCCACAC	T	patient 5	WXS	Illumina	NM_001145004	exon8	c.1534_1554del	p.512_518del	259	0.0232
OR4N4	hs37d5	15	22382867	22382867	nonsynonymous_SNV	SNP	G	G	A	patient 5	WXS	Illumina	NM_001005241	exon1	c.G395A	p.C132Y	168	0.0714
GOLGA6L1	hs37d5	15	22743252	22743252	nonsynonymous_SNV	SNP	T	T	C	patient 5	WXS	Illumina	NM_001001413	exon8	c.T1637C	p.M546T	264	0.0758
HERC2	hs37d5	15	28419587	28419587	synonymous_SNV	SNP	G	G	A	patient 5	WXS	Illumina	NM_004667	exon65	c.C10011T	p.V3337V	145	0.331
HERC2	hs37d5	15	28443631	28443631	nonsynonymous_SNV	SNP	G	G	C	patient 5	WXS	Illumina	NM_004667	exon50	c.C7904G	p.S2635C	233	0.1202
HERC2	hs37d5	15	28474849	28474849	nonsynonymous_SNV	SNP	C	C	G	patient 5	WXS	Illumina	NM_004667	exon32	c.A4954C	p.K1652Q	225	0.1244
HERC2	hs37d5	15	28475625	28475625	nonsynonymous_SNV	SNP	G	G	A	patient 5	WXS	Illumina	NM_004667	exon31	c.A4697T	p.D1566V	145	0.0828
GOLGA6B	hs37d5	15	72953642	72953642	stopgain_SNV	SNP	T	T	A	patient 5	WXS	Illumina	NM_018652	exon8	c.T602A	p.L201X	219	0.274
ARID3B	hs37d5	15	74836616	74836616	synonymous_SNV	SNP	A	A	G	patient 5	WXS	Illumina	NM_006465	exon2	c.A339G	p.K113K	128	0.0859
KIF7	hs37d5	15	90172692	90172692	nonsynonymous_SNV	SNP	C	C	A	patient 5	WXS	Illumina	NM_198525	exon17	c.G3431T	p.R1144L	62	0.129
CASKIN1	hs37d5	16	2234995	2234995	synonymous_SNV	SNP	G	G	A	patient 5	WXS	Illumina	NM_020764	exon13	c.C1281T	p.S427S	75	0.08
RRN3	hs37d5	16	15178499	15178499	splicing	SNP	G	G	A	patient 5	WXS	Illumina	0	0	0	0	176	0.1307
KIAA0430	hs37d5	16	15706553	15706553	nonsynonymous_SNV	SNP	T	T	C	patient 5	WXS	Illumina	NM_001184998	exon17	c.A3335G	p.K1112R	92	0.0978
XYLT1	hs37d5	16	17252728	17252728	nonsynonymous_SNV	SNP	C	C	A	patient 5	WXS	Illumina	NM_022166	exon6	c.G1328T	p.R443L	58	0.3793
ERN2	hs37d5	16	23712018	23712018	nonsynonymous_SNV	SNP	G	G	A	patient 5	WXS	Illumina	NM_033266	exon13	c.C1511T	p.P504L	73	0.1507
RBBP6	hs37d5	16	24574580	24574580	synonymous_SNV	SNP	C	C	T	patient 5	WXS	Illumina	NM_006910	exon11	c.C1350T	p.T450T	179	0.1117
GDPD3	hs37d5	16	30122719	30122719	nonsynonymous_SNV	SNP	T	T	C	patient 5	WXS	Illumina	NM_024307	exon7	c.A697G	p.I233V	74	0.0946
MYLPF	hs37d5	16	30389120	30389120	nonsynonymous_SNV	SNP	A	A	G	patient 5	WXS	Illumina	NM_013292	exon7	c.A409G	p.K137E	53	0.1321
CDH5	hs37d5	16	66432424	66432424	synonymous_SNV	SNP	C	C	T	patient 5	WXS	Illumina	NM_001795	exon10	c.C1551T	p.I517I	61	0.2951
CES3	hs37d5	16	67006379	67006379	nonsynonymous_SNV	SNP	C	C	T	patient 5	WXS	Illumina	NM_024922	exon11	c.C1412T	p.P471L	54	0.0926
ZFHX3	hs37d5	16	72830895	72830895	nonsynonymous_SNV	SNP	C	C	T	patient 5	WXS	Illumina	NM_006885	exon9	c.G5686A	p.A1896T	40	0.125
SPATA2L	hs37d5	16	89764300	89764300	synonymous_SNV	SNP	G	G	A	patient 5	WXS	Illumina	NM_152339	exon3	c.C717T	p.D239D	66	0.1515
KIAA0664	hs37d5	17	2598309	2598309	synonymous_SNV	SNP	C	C	A	patient 5	WXS	Illumina	NM_015229	exon16	c.G2577T	p.L859L	96	0.1562
ZZEF1	hs37d5	17	3916875	3916875	nonsynonymous_SNV	SNP	T	T	C	patient 5	WXS	Illumina	NM_015113	exon52	c.A8447G	p.E2816G	84	0.0833
ALOX15	hs37d5	17	4536747	4536747	nonsynonymous_SNV	SNP	T	T	C	patient 5	WXS	Illumina	NM_001140	exon9	c.A1210G	p.R404G	78	0.1282
SLC25A11	hs37d5	17	4842216	4842216	synonymous_SNV	SNP	G	G	A	patient 5	WXS	Illumina	NM_003562	exon3	c.C303T	p.I101I	37	0.2432
SLC25A11	hs37d5	17	4842217	4842217	nonsynonymous_SNV	SNP	A	A	T	patient 5	WXS	Illumina	NM_003562	exon3	c.T302A	p.I101N	37	0.2432
MYH4	hs37d5	17	10356212	10356212	nonsynonymous_SNV	SNP	A	A	G	patient 5	WXS	Illumina	NM_017533	exon25	c.T3149C	p.M1050T	168	0.0595
LRRC37B	hs37d5	17	30349122	30349122	synonymous_SNV	SNP	A	A	T	patient 5	WXS	Illumina	NM_052888	exon1	c.A957T	p.P319P	120	0.1083
SLFN13	hs37d5	17	33769038	33769038	nonsynonymous_SNV	SNP	C	C	A	patient 5	WXS	Illumina	NM_144682	exon5	c.G1466T	p.R489L	146	0.0616

TBC1D3F	hs37d5	17	36288278	36288278	nonsynonymous_SNV	SNP	T	T	A	patient 5	WXS	Illumina	NM_032258	exon6	c.T364A	p.L122M	376	0.0319
KRTAP4-9	hs37d5	17	39261759	39261759	nonsynonymous_SNV	SNP	G	G	A	patient 5	WXS	Illumina	NM_001146041	exon1	c.G119A	p.C40Y	131	0.0611
KRTAP4-9	hs37d5	17	39261778	39261778	synonymous_SNV	SNP	A	A	G	patient 5	WXS	Illumina	NM_001146041	exon1	c.A138G	p.V46V	117	0.0769
KRTAP4-9	hs37d5	17	39262083	39262083	nonsynonymous_SNV	SNP	A	A	C	patient 5	WXS	Illumina	NM_001146041	exon1	c.A443C	p.N148T	117	0.0684
SNF8	hs37d5	17	47014485	47014485	splicing	SNP	A	A	T	patient 5	WXS	Illumina	0	0	0	0	80	0.075
MMD	hs37d5	17	53499046	53499046	nonsynonymous_SNV	SNP	T	T	C	patient 5	WXS	Illumina	NM_012329	exon1	c.A11G	p.K4R	77	0.1039
COG1	hs37d5	17	71193529	71193529	nonsynonymous_SNV	SNP	C	C	T	patient 5	WXS	Illumina	NM_018714	exon4	c.C907T	p.P303S	64	0.2031
OTOP2	hs37d5	17	72929572	72929572	nonsynonymous_SNV	SNP	C	C	G	patient 5	WXS	Illumina	NM_178160	exon7	c.C1621G	p.L541V	36	0.1944
DNAH17	hs37d5	17	76570828	76570828	synonymous_SNV	SNP	T	T	C	patient 5	WXS	Illumina	NM_173628	exon2	c.A312G	p.T104T	57	0.1228
DNAH17	hs37d5	17	76570890	76570890	nonsynonymous_SNV	SNP	A	A	G	patient 5	WXS	Illumina	NM_173628	exon2	c.T250C	p.S84P	88	0.0795
NDUFV2	hs37d5	18	9122591	9122591	synonymous_SNV	SNP	T	T	C	patient 5	WXS	Illumina	NM_021074	exon5	c.T381C	p.V127V	248	0.0363
NDUFV2	hs37d5	18	9122611	9122611	nonsynonymous_SNV	SNP	T	T	C	patient 5	WXS	Illumina	NM_021074	exon5	c.T401C	p.V134A	241	0.0373
STARD6	hs37d5	18	51863516	51863516	synonymous_SNV	SNP	A	A	G	patient 5	WXS	Illumina	NM_139171	exon3	c.T246C	p.N82N	253	0.0316
ATP9B	hs37d5	18	77134019	77134019	synonymous_SNV	SNP	G	G	A	patient 5	WXS	Illumina	NM_198531	exon28	c.G3192A	p.T1064T	76	0.0789
ABCA7	hs37d5	19	1046239	1046239	nonsynonymous_SNV	SNP	C	C	G	patient 5	WXS	Illumina	NM_019112	exon13	c.C1456G	p.P486A	83	0.0964
PLK5	hs37d5	19	1535205	1535205	nonsynonymous_SNV	SNP	G	G	C	patient 5	WXS	Illumina	NM_001243079	exon14	c.G967C	p.G323R	63	0.127
PLIN4	hs37d5	19	4511326	4511326	synonymous_SNV	SNP	C	C	G	patient 5	WXS	Illumina	NM_001080400	exon3	c.G2604C	p.G868G	82	0.0854
MBD3L3	hs37d5	19	7056590	7056590	nonsynonymous_SNV	SNP	C	C	T	patient 5	WXS	Illumina	NM_001164425	exon2	c.G370A	p.G124S	334	0.0509
ZNF812	hs37d5	19	9801466	9801466	nonsynonymous_SNV	SNP	G	G	T	patient 5	WXS	Illumina	NM_001199814	exon6	c.C713A	p.A238E	168	0.0536
COMP	hs37d5	19	18897449	18897449	stopgain_SNV	SNP	G	G	A	patient 5	WXS	Illumina	NM_000095	exon11	c.C1147T	p.Q383X	92	0.1196
ZNF208	hs37d5	19	22170097	22170097	synonymous_SNV	SNP	C	C	T	patient 5	WXS	Illumina	NM_007153	exon3	c.G147A	p.K49K	252	0.0635
CAPN12	hs37d5	19	39227199	39227199	nonsynonymous_SNV	SNP	G	G	A	patient 5	WXS	Illumina	NM_144691	exon11	c.C1367T	p.P456L	115	0.0609
FCGBP	hs37d5	19	40384099	40384099	nonsynonymous_SNV	SNP	G	G	A	patient 5	WXS	Illumina	NM_003890	exon21	c.C9511T	p.P3171S	271	0.0369
PSG1	hs37d5	19	43372959	43372959	synonymous_SNV	SNP	G	G	T	patient 5	WXS	Illumina	NM_006905	exon4	c.C937A	p.R313R	204	0.049
ZNF227	hs37d5	19	44732619	44732619	synonymous_SNV	SNP	T	T	C	patient 5	WXS	Illumina	NM_182490	exon4	c.T81C	p.D27D	265	0.0642
CEACAM19	hs37d5	19	45182203	45182203	nonsynonymous_SNV	SNP	C	C	A	patient 5	WXS	Illumina	NM_020219	exon4	c.C654A	p.S218R	126	0.5
FOXA3	hs37d5	19	46376091	46376091	synonymous_SNV	SNP	C	C	T	patient 5	WXS	Illumina	NM_004497	exon2	c.C828T	p.P276P	124	0.0887
CNOT3	hs37d5	19	54647434	54647434	synonymous_SNV	SNP	C	C	T	patient 5	WXS	Illumina	NM_014516	exon5	c.C207T	p.N69N	65	0.1692
LILRA6	hs37d5	19	54744209	54744209	nonsynonymous_SNV	SNP	T	T	A	patient 5	WXS	Illumina	NM_024318	exon6	c.A1199T	p.Y400F	181	0.0829
LILRA6	hs37d5	19	54744794	54744794	nonsynonymous_SNV	SNP	G	G	A	patient 5	WXS	Illumina	NM_024318	exon5	c.C868T	p.H290Y	270	0.0333
LILRB1	hs37d5	19	55145452	55145452	nonsynonymous_SNV	SNP	C	C	G	patient 5	WXS	Illumina	NM_001081637	exon9	c.C1343G	p.T448S	173	0.0462
LILRB1	hs37d5	19	55145453	55145453	synonymous_SNV	SNP	C	C	T	patient 5	WXS	Illumina	NM_001081637	exon9	c.C1344T	p.T448T	172	0.0581
ZNF773	hs37d5	19	58017753	58017753	nonsynonymous_SNV	SNP	C	C	A	patient 5	WXS	Illumina	NM_198542	exon4	c.C290A	p.A97E	63	0.1905
DZANK1	hs37d5	20	18396056	18396056	nonsynonymous_SNV	SNP	G	G	C	patient 5	WXS	Illumina	NM_001099407	exon11	c.C992G	p.T331S	72	0.6806
CST1	hs37d5	20	23729763	23729763	nonsynonymous_SNV	SNP	C	C	T	patient 5	WXS	Illumina	NM_001898	exon2	c.G232A	p.V78I	108	0.1019
CDK5RAP1	hs37d5	20	31954682	31954682	nonsynonymous_SNV	SNP	A	A	C	patient 5	WXS	Illumina	NM_016408	exon12	c.T1525G	p.L509V	113	0.0531
B4GALT5	hs37d5	20	48252961	48252961	nonsynonymous_SNV	SNP	C	C	T	patient 5	WXS	Illumina	NM_004776	exon9	c.G1055A	p.G352E	97	0.1031
SLC9A8	hs37d5	20	48456108	48456108	nonsynonymous_SNV	SNP	T	T	C	patient 5	WXS	Illumina	NM_015266	exon4	c.T320C	p.I107T	132	0.1667
CABLES2	hs37d5	20	60967496	60967496	nonsynonymous_SNV	SNP	G	G	T	patient 5	WXS	Illumina	NM_031215	exon8	c.C1040A	p.T347N	65	0.1692
TPTE	hs37d5	21	10910340	10910340	synonymous_SNV	SNP	C	C	G	patient 5	WXS	Illumina	NM_199261	exon22	c.G1416C	p.L472L	352	0.0312
TPTE	hs37d5	21	10933906	10933906	nonsynonymous_SNV	SNP	A	A	T	patient 5	WXS	Illumina	NM_199261	exon17	c.T973A	p.W325R	843	0.0285
TPTE	hs37d5	21	10942923	10942923	nonsynonymous_SNV	SNP	G	G	A	patient 5	WXS	Illumina	NM_199261	exon12	c.C664T	p.R222W	403	0.0298
GABPA	hs37d5	21	27117542	27117542	synonymous_SNV	SNP	G	G	A	patient 5	WXS	Illumina	NM_001197297	exon3	c.G99A	p.A33A	97	0.0722
DOPEY2	hs37d5	21	37665854	37665854	synonymous_SNV	SNP	A	A	G	patient 5	WXS	Illumina	NM_005128	exon37	c.A6882G	p.E2294E	92	0.0978
COL6A1	hs37d5	21	47417658	47417658	synonymous_SNV	SNP	G	G	C	patient 5	WXS	Illumina	NM_001848	exon22	c.G1506C	p.P502P	59	0.1864
YBEY	hs37d5	21	47717486	47717486	nonsynonymous_SNV	SNP	C	C	G	patient 5	WXS	Illumina	NM_058181	exon5	c.C442G	p.L148V	64	0.125
POTEH	hs37d5	22	16277852	16277852	synonymous_SNV	SNP	C	C	T	patient 5	WXS	Illumina	NM_001136213	exon5	c.G1062A	p.S354S	180	0.1722
POTEH	hs37d5	22	16287661	16287661	synonymous_SNV	SNP	G	G	A	patient 5	WXS	Illumina	NM_001136213	exon1	c.C225T	p.S75S	76	0.0921
IGLL5	hs37d5	22	23237792	23237792	nonsynonymous_SNV	SNP	A	A	G	patient 5	WXS	Illumina	NM_001178126	exon3	c.A563G	p.K188R	97	0.0515
MYO18B	hs37d5	22	26176100	26176100	nonsynonymous_SNV	SNP	C	C	T	patient 5	WXS	Illumina	NM_032608	exon9	c.C2146T	p.R716W	35	0.2571
TMPRSS6	hs37d5	22	37494484	37494484	nonsynonymous_SNV	SNP	C	C	A	patient 5	WXS	Illumina	NM_153609	exon3	c.G335T	p.R112L	92	0.0761
TRIOBP	hs37d5	22	38120668	38120668	nonsynonymous_SNV	SNP	A	A	G	patient 5	WXS	Illumina	NM_001039141	exon7	c.A2105G	p.Q702R	176	0.0455
CYP2D6	hs37d5	22	42524795	42524795	synonymous_SNV	SNP	A	A	G	patient 5	WXS	Illumina	NM_000106	exon4	c.T657C	p.F219F	45	0.1556
PKDREJ	hs37d5	22	46654180	46654180	synonymous_SNV	SNP	G	G	T	patient 5	WXS	Illumina	NM_006071	exon1	c.C5040A	p.I1680I	64	0.6406
LMF2	hs37d5	22	50944487	50944487	nonsynonymous_SNV	SNP	G	G	A	patient 5	WXS	Illumina	NM_033200	exon5	c.C751T	p.R251C	37	0.2432
LUZP4	hs37d5	X	114540824	114540824	nonsynonymous_SNV	SNP	G	G	C	patient 5	WXS	Illumina	NM_016383	exon4	c.G397C	p.E133Q	80	0.075
SPANXC	hs37d5	X	140335742	140335742	nonsynonymous_SNV	SNP	G	G	C	patient 5	WXS	Illumina	NM_022661	exon2	c.C202G	p.L68V	193	0.0674
SPANXD	hs37d5	X	140785714	140785714	nonsynonymous_SNV	SNP	C	C	G	patient 5	WXS	Illumina	NM_032417	exon2	c.G202C	p.V68L	275	0.0691
SPANXD	hs37d5	X	140785739	140785739	nonsynonymous_SNV	SNP	A	A	C	patient 5	WXS	Illumina	NM_032417	exon2	c.T177G	p.F59L	306	0.0392
MAGEC1	hs37d5	X	140993875	140993875	nonsynonymous_SNV	SNP	G	G	C	patient 5	WXS	Illumina	NM_005462	exon4	c.G685C	p.A229P	291	0.0344
MAGEC1	hs37d5	X	140994087	140994087	synonymous_SNV	SNP	C	C	A	patient 5	WXS	Illumina	NM_005462	exon4	c.C897A	p.P299P	190	0.0474

Table S2: mutational details of recurrently mutated genes in study cohort

Sample ID:	Tumor Type	*NF1*	*SUZ12*	*EED*	*TP53*	*CDKN2A*
Patient1_1	MPNST	I1910fs*; het loss	E610X; het loss	het loss	het loss	
Patient1_2	Atypical Neurofibroma	I1910fs*; splice site				
Patient2	MPNST	L847P*; het loss			het loss	
Patient3	MPNST	M1L; het loss*	E324X; het loss*			het loss
Patient4	MPNST	Y489C; het loss	het loss			hom loss
Patient5	MPNST	clinical NF1		I290fs		
TCGA-QQ-A8VG	MPNST	clinical NF1; R1362X				het loss
TCGA-QQ-A8VH	MPNST	clinical NF1; splice site			Y163N	
TCGA-RN-AAAQ	MPNST	clinical NF1				hom loss
TCGA-SI-A71O	MPNST			het loss	hom loss	
TCGA-SI-A71P	MPNST	clinical NF1; P504fs				het loss
TCGA-SI-A71Q	MPNST	clinical NF1; het loss	het loss	het loss	het loss	het loss
TCGA-SI-AA8C	MPNST	clinical NF1; het loss	L574fs; het loss		het loss	hom loss

*=germline finding

het loss= predicted copy number of 1 from copy number variation analysis; hom loss = predicted copy number of 0 from copy number analysis

Brohl AS, et al. The genomic landscape of malignant peripheral nerve sheath tumors: diverse drivers of Ras pathway activation

Table S3: significant copy number variations as detected from whole exome sequencing analysis

Sample:	Chromosome	Start	End	CN predicted	ProbCall	Relevant Gene(s)
patient 1_1	4	52709318	57976527	9	1	*KIT, PDGFRA*
patient 1_1	11	73101730	134605931	1	0.962366951	*EED*
patient 1_1	17	2138984	18110326	1	0.990206224	*TP53*
patient 1_1	17	26898435	36104885	1	0.989892387	*NF1, SUZ12*
patient 2	17	2568645	8020315	1	0.969737767	*TP53*
patient 2	17	28990577	29764530	1	0.603010314	*NF1*
patient 3	9	21802701	23762254	1	0.998832018	*CDKN2A*
patient 3*	17	28999025	30380375	1	0.904650463	*NF1, SUZ12*
patient 4	9	21930980	22452207	0	0.999996462	*CDKN2A*
patient 4	17	29508703	34341463	1	0.944552336	*NF1, SUZ12*

*germline finding

Brohl AS, et al. The genomic landscape of malignant peripheral nerve sheath tumors: diverse drivers of Ras pathway activation
